# Meroterpenoids from the Fungus *Ganoderma sinensis* and First Absolute Configuration Clarification of Zizhine H

**DOI:** 10.3390/molecules25010158

**Published:** 2019-12-31

**Authors:** Yan-Jiao Yin, Dan-Ling Huang, Bin Qiu, Dan Cai, Jiao-Jiao Zhang, Shao-Xiang Wang, Da-Peng Qin, Yong-Xian Cheng

**Affiliations:** 1 School of Pharmaceutical Sciences, Shenzhen University Health Science Center, Shenzhen 518060, China; m15288241220@163.com (Y.-J.Y.); leonchemistry@szu.edu.cn (D.-L.H.); caidan_edu@163.com (D.C.); zhangjiaojiao@szu.edu.cn (J.-J.Z.); wsx@szu.edu.cn (S.-X.W.); tqindp@szu.edu.cn (D.-P.Q.); 2 College of Pharmaceutical Sciences, Yunnan University of Traditional Chinese Medicine, Kunming 650500, China; yyqiubin@aliyun.com

**Keywords:** *Ganoderma sinensis*, fungus, meroterpenoids, zizhines P-S and U, cytotoxic activity

## Abstract

Five new meroterpenoids, zizhines P-S and U (**1**−**4**,**7**), together with two known meroterpenoids (**5** and **6**) were isolated from *Ganoderma sinensis*. Their structures including absolute configurations were assigned by using spectroscopic, computational, and chemical methods. Racemics zizhines P and Q were purified by HPLC on chiral phase. Biological evaluation found that **4**, **5** and **6** are cytotoxic toward human cancer cells (A549, BGC-823, Kyse30) with IC_50_ values in the range of 63.43–80.83 μM towards A549, 59.2 ± 2.73 μM and 64.25 ± 0.37 μM towards BGC-823, 76.28 ± 1.93 μM and 85.42 ± 2.82 μM towards Kyse30.

## 1. Introduction

**Figure 1:**
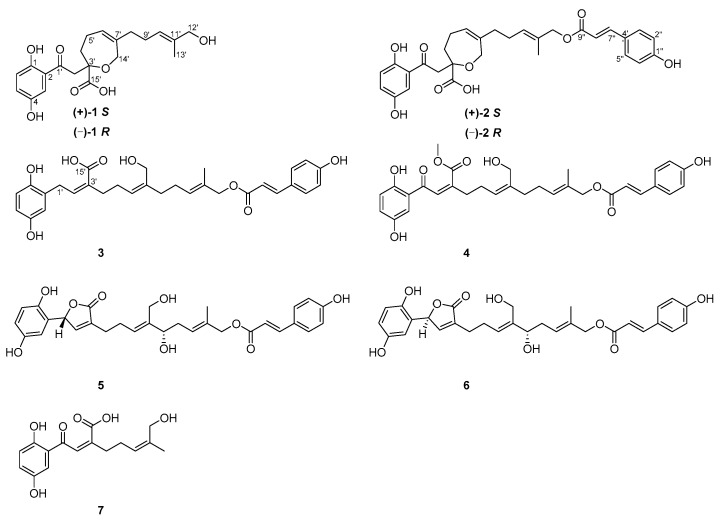
The structures of compounds **1**–**7**.

*Ganoderma* fungi, worldwide known mushrooms, are mainly distributed in the tropical and subtropics regions [1]. Due to the medicinal significance of this genus, plenty of studies have been conducted in the last decades which reveal the presence of triterpenoids, polysaccharides, alkaloids, fatty acids, nucleotides, proteins, peptides, trace elements and sterols thereof [2,3,4,5]. A recent search by SciFinder according to “*Ganoderma*” found 25,917 related papers, indicating the importance of *Ganoderma* in the scientific community. *Ganoderma* is known in China as a mythic name “immortal herbs” [6], which has been used for the treatment of a wide range of diseases such as trachitis, chronic hepatitis, neurasthenia, dyspepsia, hypertension, and tumor [7]. Despite that *Ganoderma* embraces more than 30 fungal species, so far only *G. lucidum* and *G. sinensis* are recorded in *Pharmacopoeia of People’s Republic of China* (2015 edition). We have conducted an extensive and oriented study on meroterpenoids from *G. lucidum* and found structurally and biologically intriguing meroterpenoids. [8,9,10] Inspired by these previous findings, we have embarked an investigation on *G. sinensis*, leading to the isolation of (±)-sinensilactam A and biologically important meroterpenoids with a para-hydroxycinnamyl group in the structure [11,12,13]. As a continuous study on *G. sinensis*, the current investigation resulted in the characterization of five new meroterpenoids, zizhines P−S and U (**1**−**4**,**7**) along with two previously reported meroterpenoids (**5** and **6**) (Figure 1). To reveal their biological importance, all the isolates were evaluated for their cytotoxic properties against several human cancer cells.

## 2. Results and Discussion

### 2.1. Structure Elucidation of the Compounds

**Figure 2:**
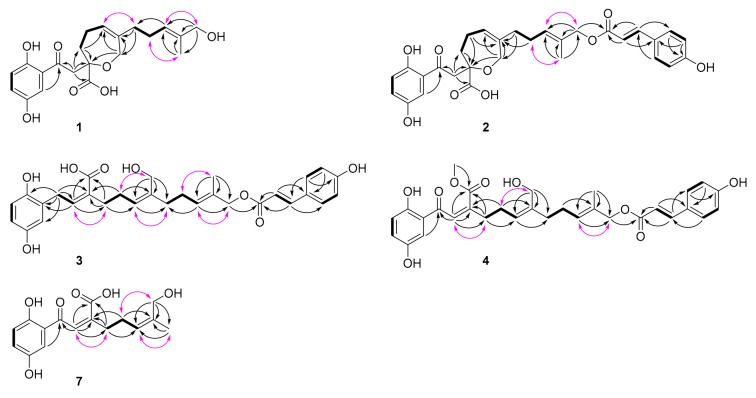
The ^1^H−^1^H COSY, Key HMBC and ROESY correlations for **1**–**4** and **7**.

Compound **1** was isolated as a yellowish gum. Its molecular formula was deduced as C_21_H_26_O_7_ by analysis of its positive HRESIMS, ^13^C-NMR, and DEPT spectra. The ^1^H-NMR spectrum of **1** (Table 1) gives a typical ABX spin system [δ_H_ 7.26 (1H, d, *J* = 3.0 Hz, H-3), 7.01 (1H, dd, *J* = 8.9, 3.0 Hz, H-5), 6.78 (1H, d, *J* = 8.9 Hz, H-6)]. The ^13^C-NMR and DEPT spectra (Table 1) show one methyl, five sp^3^ methylenes, two sp^3^ oxygenated methylenes, five sp^2^ methines, eight nonprotonated carbons (including a ketone group at δ_C_ 204.1 and a carboxyl group at δ_C_ 177.4). Within the context of meroterpenoids isolated from *Ganoderma* species, these data prompted us to associate **1** with meroterpenoid. Inspection of 2D NMR data of **1** reveals ^1^H−^1^H COSY correlations (Figure 2) of H_2_-4′/H_2_-5′/H-6′ (δ_H_ 5.47) and H_2_-8′/H_2_-9′/H-10′ (δ_H_ 5.38) and HMBC correlations (Figure 2) of H_2_-12′ (δ_H_ 3.91), H_3_-13′/C-10′, C-11′ (δ_C_ 136.4), H_2_-12′/C-13′, H_2_-14′ (δ_H_ 4.26, 4.21), H_2_-8′/C-6′, C-7′, H_2_-14′/C-8′, and Ha-5′/C-7′, indicating the presence of two isoprenyl moieties in the side chain of **1.** Besides, the observation of HMBC correlations of H_2_-2′/C-1′ (δ_C_ 204.1), C-4′, C-15′ (δ_C_ 177.4), Hb-4′/C-3′ (δ_C_ 82.3), C-15′, H_2_-14′/C-3′ in **1** suggest the presence of another isoprenyl residue and a seven-membered ring via the formation of C-formed by C-3′-O-C-14′. The terpenoidal group is connected with the, benzene ring via C-2-C-1′ based on the HMBC observation of H-3, H_2_-2′/C-1′. As a result, the planar structure of **1** was assigned (Figure 1).

**Figure 3:**
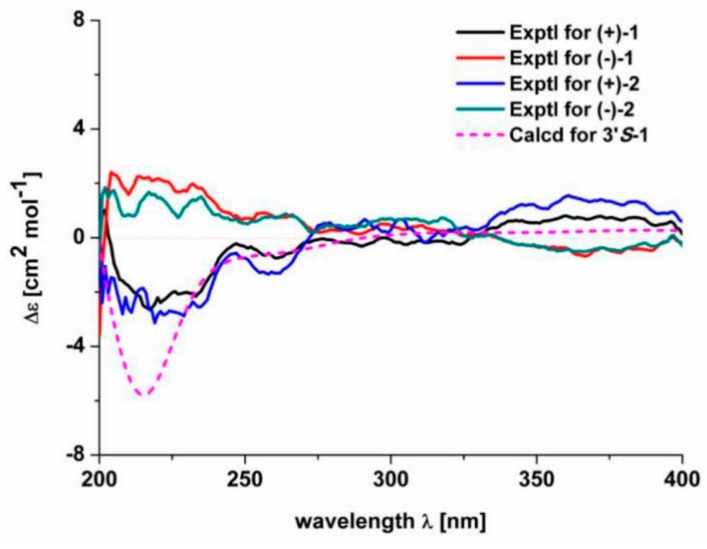
The calculated and experimental ECD spectra of **1** and **2**.

As for the geometry of **1**, significant ROESY correlation (Figure 2) of H-10′/H_2_-12′ shows that *∆*^10′(11′)^ double bond is *E* configuration. It was noted that **1** was isolated as a racemic mixture indicated by its chiral HPLC analysis. Racemic **1** was further separated by HPLC on chiral phase to afford (+)-**1** and (−)-**1**, respectively. There is only one chiral center in the structure of **1**. To clarify the absolute configurations of (+)-**1** and (−)-**1**, ECD calculations were carried out at B3LYP/6-311+g(2d,p) level. It was found that the experiment ECD spectrum of (+)-**1** matches well with the calculated ECD spectrum of (*S*)-**1** (Figure 3). Thus, the absolute configurations of (−)-**1** and (+)-**1** were respectively assigned as 3′*R* and 3′*S*. Finally, the (−)-**1** and (+)-**1** was determined to be (*R*,*E*)-2-(2-(2,5-dihydroxyphenyl)-2-oxoethyl)-6-(5-hydroxy-4-methylpent-3-en-1-yl)-2,3,4,7-tetrahydrooxepine-2-carboxylic acid and (*S*,*E*)-2-(2-(2,5-dihydroxyphenyl)-2-oxoethyl)-6-(5-hydroxy-4-methylpent-3-en-1-yl)-2,3,4,7-tetrahydrooxepine-2-carboxylic acid respectively. Hence, the structure of **1**, named zizhine P, was deduced.

Compound **2** has a molecular formula of C_30_H_32_O_9_ deduced from its positive HRESIMS, ^13^C-NMR, and DEPT spectra. After careful analysis of the data of **1** and **2** (Table 1), it was found that the only difference between **2** and **1** is that a 4-hydroxycinnamic acid group is connected to C-12′ via an oxygen atom, this conclusion is supported by the HMBC correlation (Figure 2) of H_2_-12′ (δ_H_ 4.56)/C-9′′ (δ_C_ 169.1). The stereochemistry of 2 was assigned using ROSEY evidences. The ROESY correlation (Figure 2) of H_2_-9′ (δ_H_ 2.14)/H_3_-13′ (δ_H_ 1.68) indicate that the configuration of *Δ*^10′(11′)^ double bond is *E-*form. The large coupling constants (nearly 16.0 Hz) of the one pair of olefinic protons suggest the *trans* form for the *Δ*^7′’(8′′)^ double bond. Compound 2 was also isolated as a racemic mixture. Further separation by chiral phase HPLC afforded (−)-**2** and (+)-**2**. To clarify their absolute configurations, ECD curve comparison with **1** was used. It is obvious that the experiment CD spectrum of (+)-**2** agrees well with the experiment CD spectrum of (+)-**1** (Figure 3). The absolute configurations of (−)-**2** and (+)-**2** were thus assigned as 3′*R* and 3′*S*, respectively. Ultimately, the (−)-**2** and (+)-**2** was determined to be (*R*)-2-(2-(2,5-dihydroxyphenyl)-2-oxoethyl)-6-((*E*)-5-(((*E*)-3-(4-hydroxyphenyl)acryloyl)oxy)-4-methylpent-3-en-1-yl)-2,3,4,7-tetrahydrooxepine-2-carboxylic acid and (*S*)-2-(2-(2,5-dihydroxyphenyl)-2-oxoethyl)-6-((*E*)-5-(((*E*)-3-(4-hydroxyphenyl)acryloyl)oxy)-4-methylpent-3-en-1-yl)-2,3,4,7-tetrahydrooxepine-2-carboxylic acid respectively. In this way, the structure of **2** was deduced and named zizhine Q.

The molecular formula of compound **3** was was determined to be C_30_H_34_O_8_ by analysis of its HRESIMS, ^13^C-NMR, and DEPT spectra. The ^1^H-NMR spectrum of **3** (Table 2) shows five aromatic signals including an ABX spin system at δ_H_ 6.66 (2H, H-3 and H-6), 6.54 (1H, dd, *J* = 8.5, 3.0 Hz, H-5), and an AA′BB′ system [δ_H_ 6.89 (2H, d, *J* = 8.6 Hz, H-2′′ and H-6′′), δ_H_ 7.55 (2H, d, *J* = 8.6 Hz, H-3′′ and H-5′′)]. The ^13^C-NMR and DEPT spectra (Table 2) display one methyl (δ_C_ 13.3), seven sp^3^ methylenes (including two oxygenated), twelve sp^2^ methines, and ten nonprotonated carbons (three sp^2^ oxygenated, one carbonyl at δ_C_ 169.5 and one ester carbonyl at δ_C_ 166.5). These data are similar to those of zizhine K [13]. The only difference is that the ketone group in zizhine K is reduced to a sp^3^ methylene in **3**. This alteration is supported by the HMBC correlations (Figure 2) of H_2_-1′ (δ_H_ 3.70)/C-1, C-2, C-3, C-2′, C-3′, and C-15′ (δ_C_ 169.5). As for the stereochemistry of **3**, ROESY correlations (Figure 2) of H-2′ (δ_H_ 6.03)/H_2_-4′ (δ_H_ 2.35), H_2_-5′ (δ_H_ 2.25)/H_2_-14′ (δ_H_ 4.10), H-10′ (δ_H_ 5.51)/H_2_-12′ (δ_H_ 4.54), and the coupling constant of H-7′′ (δ_H_ 7.62, 1H, d, *J* = 16.0 Hz) indicate the*Δ*^2′(^^3′)^ and *Δ*^6′(^^7′)^ double bonds are *Z* form, and the *Δ*^10′(^^11′)^ and *Δ*^7′′(^^8′′)^ double bonds are *E* form. As a result, compound **3** was identified as (2*Z*,5*Z*,9*E*)-2-(2-(2,5-dihydroxyphenyl)ethylidene)-6-(hydroxymethyl)-11-(((*E*)-3-(4-hydroxyphenyl)acryloyl)oxy)-10-methylundeca-5,9-dienoic acid and named zizhine R.

Compound **4** has a molecular formula of C_31_H_34_O_9_ deduced from its HRESIMS, ^13^C-NMR, and DEPT spectra. It bears the same carbon skeleton and geometry as those of zizhine K [13] by inspection of their NMR spectra (Table 2). The only difference between them is that an oxygenated methyl (δ_C_ 52.7) in **4** instead of a hydroxyl in zizhine K connected with C-15′ (δ_C_ 170.1) is observed, which is supported by the obvious HMBC correlation (Figure 2) of -OCH_3_ (δ_H_ 3.66)/C-15′. Moreover, the ROESY correlations (Figure 3) of H-2′ (δ_H_ 6.89)/H_2_-4′ (δ_H_ 2.52), H_2_-5′ (δ_H_ 2.39)/H_2_-14′ (δ_H_ 4.13), H-10′ (δ_H_ 5.52)/H_2_-12′ (δ_H_ 4.54), and the coupling constant of H-7′′ (δ_H_ 7.60, 1H, d, *J* = 15.9 Hz) indicate that the*Δ*^2′(^^3′)^ and *Δ*^6′(^^7′)^ double bonds are *Z* forms, and the *Δ*^10′(^^11′)^ and *Δ*^7′′(^^8′′)^ double bonds are *E* forms. As a result, compound **4** was identified as (2*Z*,5*Z*,9*E*)-methyl 2-(2-(2,5-dihydroxyphenyl)-2-oxoethylidene)-6-(hydroxymethyl)-11-(((*E*)-3-(4-hydroxyphenyl)acryloyl)oxy)-10-methylundeca-5,9-dienoate and named zizhine S.

**Figure 4:**
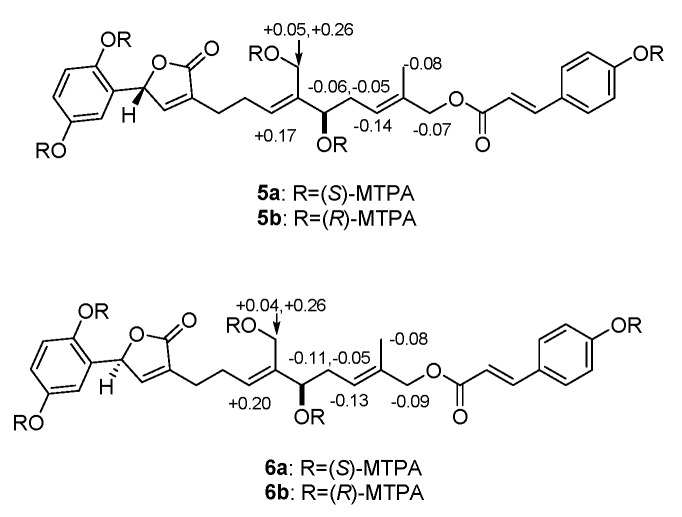
Δδ(*S*-*R*) values for the the Mosher’s esters of **5** and **6** in pyridine-*d*_5_.

**Figure 5:**
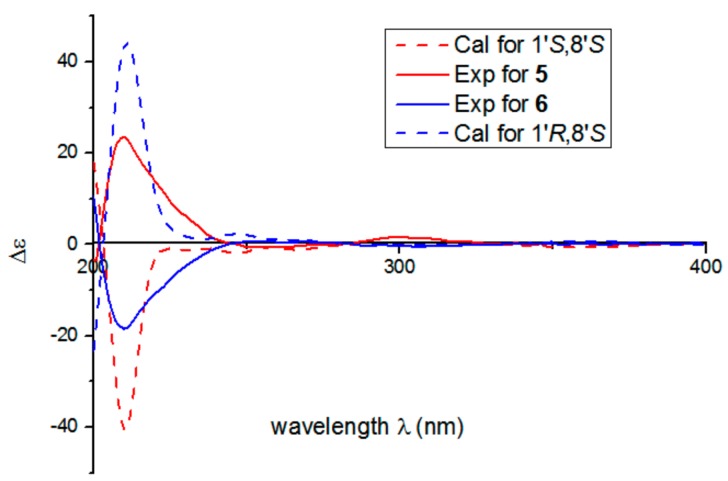
The calculated and the experimental ECD spectra of **5** and **6**.

Compounds **5** and **6** was isolated by chiral HPLC. Their planar structures were identified as that of zizhine H. There are two chiral centers in **5** and **6**, the absolute configuration at C-8′ of **5** and **6** were both assigned as *S* form according to the Mosher’s method. Briefly, treatment of **5** with (*R*)- or (*S*)-a-methoxy-atrifluoromethyl phenylacetic acyl chloride (MTPA-Cl) in deuterated pyridine was carried out to acquire the (*S*)-MTPA ester (**5a**) and (*R*)-MTPA ester (**5b**) (Figure 4), respectively. Analysis of the ^1^H-NMR signals of **5a** and **5b** indicates a 8′*S* configuration judged from the*Δ*δ_H_ values of **5a** and **5b**. In the same manner as that of **5**, the absolute configuration of C-8′ of **6** was identified as *S*. To clarify the stereochemistry at C-1′ in 5 and **6**, ECD calculations of two model compounds **5c** and **6c** (Figure S62) were performed. It was found that the configuration at C-8′ has no influence on CD curves of **5** or **6** which means that CD comparison of **5** or **6** with similar compounds makes sense to assign the absolute configuration at C-1′. With this idea, ECD comparisons between **5** or **6** with those of (+)-ganocapenoid A [14] and (−)-ganocapenoid A [14] were conducted. The results show that the CD spectra of **5** and **6** agree well with those of (+)-ganocapenoid A and (−)-ganocapenoid A, respectively, indicating the absolute configurations of **5** and **6** are 1′*R,*8′*S* and 1′*S,*8′*S*, respectively. In fact, this conclusion is also identical with that of ECD calculations of **5c** and **6c** (Figure 5).

It was noted that zizhine H has been incorrectly reported by us [13] as enantiomers. Our present results show that **5** and **6** are epimers rather than enantiomers. In this case, a careful analysis of the NMR spectra of zizhine H by Luo found that pairs of peaks are present, supporting our current conclusion. After the revision and clarification of the structures **5** and **6**, we renamed them as zizhine T for **5** ((*E*)-(*S*,2*E*,6*E*)-9-((*R*)-5-(2,5-dihydroxyphenyl)-2-oxo-2,5-dihydrofuran-3-yl)-5-hydroxy-6-(hydroxymethyl)-2-methylnona-2,6-dien-1-yl 3-(4-hydroxyphenyl)acrylate) and 1′-epimer of zizhine T for **6** ((*E*)-(*S*,2*E*,6*E*)-9-((*S*)-5-(2,5-dihydroxyphenyl)-2-oxo-2,5-dihydrofuran-3-yl)-5-hydroxy-6-(hydroxymethyl)-2-methylnona-2,6-dien-1-yl 3-(4-hydroxyphenyl)acrylate).

Compound **7** has the molecular formula of C_16_H_18_O_6_ (eight degrees of unsaturation) on the basis of its HRESIMS, ^13^C-NMR, and DEPT spectra. The ^1^H-NMR spectrum of **7** (Table 3) shows an ABX spin system at δ_H_ 7.15 (1H, d, *J* = 3.0 Hz, H-3), 7.04 (1H, dd, *J* = 9.0, 3.0 Hz, H-5), 6.84 (1H, d, *J* = 9.0 Hz, H-6). The ^13^C-NMR and DEPT spectra (Table 3) display one methyl (δ_C_ 21.3), two sp^3^ methylenes, one sp^3^ oxygenated methylene, five sp^2^ methines, and seven quaternary carbons (one carbonyl at δ_C_ 199.0 and one carboxyl at δ_C_ 171.5). These data resemble those of fornicin D [15]. The only difference between them is that one more hydroxyl group attached to the terminal methyl in **7** is observed, gainning support by the obvious HMBC correlaations (Figure 3) of H_2_-8′ (δ_H_ 4.03)/C-6′ (δ_C_ 127.4), C-7′ (δ_C_ 137.2), C-9′ (δ_C_ 21.3). For the stereochemistry of **9**, the ROESY correlations (Figure 3) of H-2′ (δ_H_ 7.63)/H_2_-4′ (δ_H_ 2.66), H_2_-5′ (δ_H_ 2.28)/H_2_-8′ (δ_H_ 4.03), H-6′ (δ_H_ 5.22)/H_3_-9′ (δ_H_ 1.62), indicate that the double bonds *Δ*^2′(^^3′)^ and *Δ*^6′(^^7′)^ are both *Z* forms. Thus, the compound **7** was deduced as (2*Z*,5*Z*)-2-(2-(2,5-dihydroxyphenyl)-2-oxoethylidene)-7-hydroxy-6-methylhept-5-enoic acid and named zizhine U.

### 2.2. Biological Evaluation

*Ganoderma* fungi have been reported to be beneficial for cancer patients [16]. The responsible compounds for cancer might be triterpenoids [17,18], polysacchride [19,20,21]. Meroterpenoids are widely present in the genus *Ganoderma* [22]. However, such compounds were largely ignored before 2013. Thereafter, increasing numbers of meroterpenoids were characterized by us. Our previous study disclosed that meroterpenoids are also in vitro active toward cancer cells [23,24,25]. Whether meroterpenoids resulting from the present study are also potent against cancer cells needs examination. Herein, all the isolated compounds were evaluated for their cytotoxic activity toward three human cancer cell lines and HDF (normal human embryonic lung fibroblasts). It was found that only BGC-823 and KYSE30 cells are sensitive to compounds **5** and **6**, and A549 cells are sensitive to compound **4**, all the other compounds are not active (Table 4). Although the cytotoxic potential of the active compounds are not so strong indicated by their larger IC_50_ values, they appear to be not harmful to human normal cells (HDF) with IC_50_ values larger than 160 μM. Cytotoxicity assay is just one approach to evaluate the role of the compounds against cancer, the derivatives of such meroterpenoids was found to be active toward Protein Tyrosine Phosphatase 1B (PTP1B) [26], indicating the usefulness of such meroterpenoids in cancer from an alternative aspect. Therefore, it is necessary to explore new screening methods to gain a deep insight into the biological role of the isolated meroterpenoids.

## 3. Experimental Section

### 3.1. General Procedures

Optical rotations were measured on a Bellingham + Stanley ADP 440 + digital 9 polarimeter (Bellingham & Stanley, Kent, UK). UV spectra were obtained on a Shimadzu UV-2600 spectrometer (Shimadzu Corporation, Tokyo, Japan). CD spectra were measured on a Chirascan instrument (Agilent Technologies, Santa Clara, CA, USA). NMR spectra were recorded on a Bruker AV-500 and AV-600 spectrometer (Bruker, Karlsruhe, Germany) with TMS as an internal standard. HRESIMS of was collected by a Shimazu LC-20AD AB SCIEX triple TOF 5600+ MS spectrometer (Shimadzu Corporation, Tokyo, Japan) or a Waters Xevo G2-XS QTOF. MCI gel CHP 20P (75–150 μm, Tokyo, Japan), C-18 silica gel (40–60 μm; Daiso Co., Tokyo, Japan), YMC gel ODS-A-HG (40–60 μm; YMC Co., Tokyo, Japan), silica gel (200–300 mesh; Qingdao Marine Chemical Inc., Qingdao, China), silica gel GF254 (80–100 mesh, Qingdao Marine Chemical Inc., China) and Sephadex LH-20 (Amersham Biosciences, Uppsala, Sweden) were used for column chromatography (CC). Semi-preparative HPLC was taken on a saipuruisi chromatograph with a Phenomenex Kinetex (250 mm × 10 mm, i.d., 5 μm) or a YMC-Pack ODS-A column (250 mm × 10 mm, i.d., 5 μm). Preparative HPLC was taken on a Chuangxin-Tongheng chromatograph equipped with a Thermo Hypersil GOLD-C_18_ column (250 × 21.2 mm, i.d., 5 μm). Racemic compounds and epimers were purified by chiral HPLC on a Daicel Chiralpak column (IC, 250 mm × 10 mm, i.d., 5 μm) and a Daicel Chiralpak column (IC, 250 mm × 4.6 mm, i.d., 5 μm).

### 3.2. Fungal Material

The fruiting bodies of *G*. *sinensis* were purchased from Tongkang Pharmaceutical Co. Ltd. in Guangdong Province, China, in September 2018. The material was authenticated by Prof. Xiang-Hua Wang at Kunming Institute of Botany, Chinese Academy of Sciences, China, and a voucher specimen (CHYX-0621) is deposited at School of Pharmaceutical Sciences, Shenzhen University Health Science Center, China.

### 3.3. Extraction and Isolation

The powdered fruiting bodies of *G*. *sinensis* (93.0 kg) were extracted by reflux with 80% EtOH (3 × 300 L × 3 h) to give a crude extract. The extract was suspended in water and partitioned with EtOAc to obtain an EtOAc-soluble extract (1.6 kg). The EtOAc extract was divided into ten parts (Fr.1-Fr.10) by using a MCI gel CHP 20P column eluted with aqueous MeOH (40–100%).

Fr.7 (40.0 g) was subjected to Sephadex LH-20 CC (MeOH) to obtain six parts (Fr.7.1–Fr.7.6). Among them, Fr.7.3 (14.0 g) was cut by a C-18 column (MeOH/H_2_O, 30–100%) to afford eight portions (Fr.7.3.1–Fr.7.3.8). Of which, Fr.7.3.5 (4.7 g) was divided by Sephadex LH-20 (MeOH) to provide Fr.7.3.5.1 and Fr.7.3.5.2. The first part (2.6 g) was further divided by silica gel CC eluted with increasing MeOH in CH_2_Cl_2_ (200:1–1:1) to afford Fr.7.3.5.1.1–Fr.7.3.5.1.8. Fr.7.3.5.1.6 (731.0 mg) was cut into three parts (Fr.7.3.5.1.6.1–Fr.7.3.5.1.6.3) by preparative thin layer chromatography (PTLC) (EtOAc:EtOH:H_2_O = 15:2:1). One portion from PTLC (220.0 mg) (Rf = 0.5) was first submitted to Sephadex LH-20 (MeOH) to get two parts (Fr.7.3.5.1.6.3.1 and Fr.7.3.5.1.6.3.2). Fr.7.3.5.1.6.3.2 (121.0 mg) was separated by preparative HPLC [MeOH/H_2_O (0.05% TFA), 35%-100%] to obtain five portions (Fr.7.3.5.1.6.3.2.1–Fr.7.3.5.1.6.3.2.5). Fr.7.3.5.1.6.3.2.3 (21.0 mg) was purified by semi-preparative HPLC (acetonitrile/H_2_O containing 0.05% TFA, 40%, flow rate: 3 mL/min) to afford compound **9** (1.1 mg, t_R_ = 32.7 min). Fr.7.3.6 (4.5 g) was further divided by silica gel CC eluted with gradient CH_2_Cl_2_/MeOH (100:1–1:1) to obtain Fr.7.3.6.1–Fr.7.3.6.8. Among them, Fr.7.3.6.5 (251.0 mg) was submitted to preparative HPLC [MeOH/H_2_O (0.05% TFA), 30%-100%] to provide four portions (Fr.7.3.6.5.1–Fr.7.3.6.5.4). Fr.7.3.6.5.1 (51.0 mg) was first submitted to semi-preparative HPLC [MeOH/H_2_O (0.05% TFA), 57%, flow rate: 3 mL/min] to obtain 3.0 mg, and then purified by semi-preparative HPLC (acetonitrile/H_2_O containing 0.05% TFA, 31%, flow rate: 3 mL/min) to yeild compound **1** (1.8 mg, t_R_ = 26.0 min). Fr.7.3.6.6 (782.0 mg) was separated by preparative HPLC [MeOH/H_2_O (0.05% TFA), 35–100%] to obtain five portions (Fr.7.3.6.6.1–Fr.7.3.6.6.5). Among them, Fr.7.3.6.6.2 (166.0 mg) was cut by preparative HPLC [MeOH/H_2_O (0.05% TFA), 35%-100%] to obtain eight parts (Fr.7.3.6.6.2.1–Fr.7.3.6.6.2.8). Of which, Fr.7.3.6.6.2.5 (70.0 mg) was fractionated by semi-preparative HPLC (acetonitrile/H_2_O containing 0.05% TFA, 40%, flow rate: 3 mL/min) to obtain four parts (Fr.7.3.6.6.2.5.1–Fr.7.3.6.6.2.5.4). Fr.7.3.6.6.2.5.3 (43.0 mg) was further purified by chiral HPLC (n-hexane/ethanol, 82:18, flow rate: 3 mL/min) to yeild compounds **5** (12.4 mg, t_R_ = 18.2 min) and **6** (12.2 mg, t_R_ = 22.3 min). 

Fr.8 (68.0 g) was gel filtrated over Sephadex LH-20 (MeOH) to provide six parts (Fr.8.1–Fr.8.6). Of which, Fr.8.3 (34.0 g) was separated by a C-18 silica gel column eluted with aqueous MeOH (40–100%) to afford eight portions (Fr.8.3.1–Fr.8.3.8). Among them, Fr.8.3.4 (28.2 g) was divided by silica gel CC eluted with CH_2_Cl_2_:MeOH (50:1–1:1) to afford five parts (Fr.8.3.4.1–Fr.8.3.4.5). 100.0 mg was taken out of Fr.8.3.4.5 (13.0 g) and purified by semi-preparative HPLC (acetonitrile/H_2_O containing 0.05% TFA, 38%, flow rate: 3 mL/min) to yeild compound **3** (31.3 mg, t_R_ = 33.1 min). Fr.8.3.6 (1.2 g) was submitted to Sephadex LH-20 (MeOH) to afford four parts (Fr.8.3.6.1–Fr.8.3.6.4). Among them, Fr.8.3.6.2 (369.0 mg) was separated by a C-18 silica gel column eluted with aqueous MeOH (40–100%) to provide Fr.8.3.6.2.1–Fr.8.3.6.2.11. Of which, Fr.8.3.6.2.6 (145.0 mg) was cut into eight portions (Fr.8.3.6.2.6.1–Fr.8.3.6.2.6.8) by PTLC (CH_2_Cl_2_:MeOH = 8:1). Fr.8.3.6.2.6.3 (33.0 mg) (Rf = 0.8) was gel filtrated over Sephadex LH-20 (MeOH) to afford 13.0 mg followed by purification by semi-preparative HPLC (acetonitrile/H_2_O containing 0.05% TFA, 50%, flow rate: 3 mL/min) to afford compound **4** (5.88 mg, t_R_ = 29.0 min). Fr.8.3.6.3 (377.0 mg) was separated by PTLC (CH_2_Cl_2_:MeOH = 8:1) to obtain five portions (Fr.8.3.6.3.1–Fr.8.3.6.3.5). Among them, Fr.8.3.6.3.4 (55.0 mg) (Rf = 0.3) was gel filtrated over Sephadex LH-20 (MeOH) to obtain 38.0 mg, then was purified by semi-preparative HPLC (acetonitrile/H_2_O containing 0.05% TFA, 45%, flow rate: 3 mL/min) to yeild compound **2** (10.2 mg, t_R_ = 20.1 min). 

### 3.4. Compound Characterization Data

(±)-Compound **1**: yellowish gum; UV (MeOH) *λ*_max_ (log*ε*) 386 (3.56), 259 (3.87), 227 (4.16) nm; {[α]20 *D* −8.2 (*c* 0.06, MeOH); CD (MeOH) *Δε*_214_ +1.33, *Δε*_2__49_ +0.39, *Δε*_2__56_ +0.54, *Δε*_276_ +0.11, *Δε*_292_ +0.14, *Δε*_361_ −0.24; (−)-**1**}; {[α]_D_^2^^0^ +8.0 (*c* 0.05, MeOH); CD (MeOH) *Δε*_21__7_ −1.38, *Δε*_2__47_ −0.12, *Δε*_2__57_ −0.35, *Δε*_2__77_ −0.02, *Δε*_2__90_ −0.17, *Δε*_3__60_ +0.42; (+)-**1**}; HRESIMS *m*/*z* 391.1762 [M + H]^+^ (calcd for C_21_H_2__7_O_7_, 391.1757); ^1^H and ^13^C-NMR data see Table 1.

(±)-Compound **2**: yellow gum; UV (MeOH) *λ*_max_ (log*ε*) 363 (3.40), 314 (4.24), 263 (3.88), 228 (4.28) nm; {{[α]20 *D* −10.6 (*c* 0.39, MeOH); CD (MeOH) *Δε*_2__09_ +0.57, *Δε*_2__17_ +1.21, *Δε*_2__27_ +0.53, *Δε*_235_ +1.09, *Δε*_251_ +0.37, *Δε*_264_ +0.64, *Δε*_273_ +0.14, *Δε*_309_ +0.50, *Δε*_364_ −0.35; (−)-**2**}; {[α]_D_^2^^0^ +14.0 (*c* 0.28, MeOH); CD (MeOH) *Δε*_2__08_ −1.82, *Δε*_2__19_ −2.04, *Δε*_2__29_ −1.88, *Δε*_2__34_ −1.66, *Δε*_2__50_ −0.48, *Δε*_262_ −0.85, *Δε*_275_ +0.29, *Δε*_312_ −0.12, *Δε*_361_ +1.01; (+)-**2**}; HRESIMS *m*/*z* 559.1947 [M + Na]^+^ (calcd for C_30_H_32_O_9_Na, 559.1944); ^1^H and ^13^C-NMR data see Table 1.

Compound **3**: yellow gum; UV (MeOH) *λ*_max_ (log*ε*) 309 (4.17), 202 (4.46) nm; HRESIMS *m*/*z* 523.2324 [M + H]^+^ (calcd for C_30_H_35_O_8_, 523.2332); ^1^H and ^13^C-NMR data see Table 2.

Compound **4**: yellow gum; UV (MeOH) *λ*_max_ (log*ε*) 312 (3.83), 202 (4.08) nm; HRESIMS *m*/*z* 573.2101 [M + Na]^+^ (calcd for C_31_H_34_O_9_Na_,_ 573.2101); ^1^H and ^13^C-NMR data see Table 2.

Compound **5**: yellow gum; UV (MeOH) *λ*_max_ (log*ε*) 310 (4.20), 220 (4.20) nm; {[α]20 *D* +42.2 (*c* 0.32, MeOH); CD (MeOH) *Δε*_21__0_ +16.74, *Δε*_2__55_ −0.42, *Δε*_3__02_ +1.14; (+)-**5**; HRESIMS *m*/*z* 559.1945 [M + Na]^+^ (calcd for C_30_H_32_O_9_Na, 559.1944); ^1^H and ^13^C-NMR data see Table 3.

Compound **6**: yellow gum; UV (MeOH) *λ*_max_ (log*ε*) 310 (4.19), 220 (4.19) nm; {[α]20 *D* −32.4 (*c* 0.28, MeOH); CD (MeOH) *Δε*_21__0_ −13.58, *Δε*_2__56_ +0.54, *Δε*_3__06_ −0.35; (−)-**6**; HRESIMS *m*/*z* 554.2371 [M + NH_4_]^+^; (calcd for C_30_H_36_O_9_N, 554.2385); ^1^H and ^13^C-NMR data see Table 3.

Compound **7**: yellow gum; UV (MeOH) *λ*_max_ (log*ε*) 377 (3.17), 260 (3.63), 221 (3.79), 203 (3.94) nm; HRESIMS *m*/*z* 307.1188 [M + H]^+^ (calcd for C_16_H_19_O_6_, 307.1182); ^1^H and ^13^C-NMR data see Table 4.

### 3.5. MTPA Esterification of **5** and **6**

The absolute stereostructure of **5** and **6** was confirmed by Mosher’s method [27]. Compound **5** (1.0 mg) was dissolved in 1 mL of anhydrous deuteration pyridine, which was divided into two equal portions in NMR sample tube. To each portion was added 2 μL of either *R*-MTPA-Cl or *S*-MTPA-Cl to give *S*-MTPA ester (**5a**) or *R*-MTPA ester (**5b**) derivatives, and then the mixtures was kept at room temperature for 2 h. Finally, without purification, the ^1^H-NMR of the mixtures was tested. Preparation of the MTPA derivatives of **6** is same as that of **5**.

### 3.6. Cell Viability Assay

All cell lines were purchased from the Cell Bank of China Science Academy (Shanghai, China) and maintained in Dulbecco’s Modified Eagle Medium (DMEM) supplemented with 10% fetal bovine serum and 100 U/mL penicillin-streptomycin, and incubated at 37 °C in an atmosphere of 5% CO_2_. Cell viability was evaluated by the CCK8 assay kit (Dojindo Laboratories, Tokyo, Japan) according to the manufacturer’s instructions. Exponentially growing cells were seeded at 2–8 × 10^3^ cells per well in 96-well culture plates for 24 h. Cells were exposed to increasing concentrations (0–80 μM) of **4**, **5**, **6**, or 5-FU for 48 h. The equal volume of DMSO was used as the solvent control. CCK8 solution (10 μL) was added to each well and incubated for another 1–4 h. Light absorbance of the solution was measured at 450 nm (Epoch 2; BioTek Instruments, Inc. Winooski, VT, USA). The IC_50_ values were calculated using the GraphPad prism 7 and analyzed by fitting a curve using nonlinear regression [28,29].

## 4. Conclusions

To conclude, the present investigation on *G. sinensis* led to the characterization of five new meroterpenoids. With the aid of ECD calculations and chemical methods, the ambiguous structure of zizhine H was firstly clarified as a pair of epimers. Biological evaluation of these meroterpenoids disclosed that such type of compounds might be beneficial for the preventive or treatment of cancer. The current study will add new facets for chemical profiling of *Ganoderma* fungal species.

## Figures and Tables

**Table 1:** ^1^H and ^13^C-NMR data of **1** and **2**.

No.	1 *^a^*		No.	2 *^b^*	
	δ_H_ (*J* in Hz)	δ_C_		δ_H_ (*J* in Hz)	δ_C_
1		156.7	1		156.7
2		121.0	2		121.0
3	7.26, d (3.0)	115.9	3	7.26, d (2.9)	115.9
4		150.6	4		150.6
5	7.01, dd (8.9, 3.0)	126.1*^c^*	5	7.01, dd (8.9, 2.9)	126.1
6	6.78, d (8.9)	119.7	6	6.78, d (8.9)	119.7
1′		204.1	1′		204.0
2′	3.78, d (16.7)	45.4	2′	3.79, d (17.3)	45.4
	3.38, d (16.7)			3.34, d (17.3)	
3′		82.3	3′		82.2
4′	2.35, overlap	36.2	4′	2.33, overlap	36.2
	2.21, m			2.20, m	
5′	2.35, overlap	24.6	5′	2.33, overlap	24.6
	2.26, m			2.25, m	
6′	5.47, br s	125.8*^d^*	6′	5.47, overlap	126.1
7′		141.1	7′		140.8
8′	1.97, m	36.7	8′	1.99, m	36.5
9′	2.11, m	27.5	9′	2.14, m	27.5
10′	5.38, t (7.1)	126.0 *^c^*^,*d*^	10′	5.47, overlap	129.5
11′		136.4	11′		132.0
12′	3.91, s	68.9	12′	4.56, s	70.8
13′	1.63, s	13.7	13′	1.68, s	14.1
14′	4.26, d (16.1)	66.9	14′	4.27, d (16.1)	66.8
	4.21, d (16.1)			4.18, d (16.1)	
15′		177.4	15′		177.2
			1′′		161.3
			2′′, 6′′	6.80, d (8.6)	116.8
			3′′, 5′′	7.45, d (8.6)	131.2
			4′′		127.1
			7′′	7.61, d (15.9)	146.5
			8′′	6.33, d (15.9)	115.2
			9′′		169.1

*^a^* Recorded at 500 MHz for ^1^H and 150 MHz for ^13^C-NMR in methanol-*d*_4_. *^b^* Recorded at 500 MHz for ^1^H and 125 MHz for ^13^C-NMR in methanol-*d*_4_. *^c,d^* Signals with the same symbol might be interchangeable.

**Table 2:** ^1^H- and ^13^C-NMR data of **3** and **4.**

No.	3 *^a^*		No.	4 *^b^*	
	δ_H_ (*J* in Hz)	δ_C_		δ_H_ (*J* in Hz)	δ_C_
1		148.1	1		157.0
2		126.1 *^c^*	2		120.6
3	6.66, overlap	116.7	3	7.11, d (2.9)	116.2
4		150.4	4		150.7
5	6.54, dd (8.5, 3.0)	113.8	5	7.01, dd (8.9, 2.9)	126.2
6	6.66, overlap	114.7	6	6.81, d (8.9)	119.7
1′	3.70, d (7.9)	30.5	1′		197.6
2′	6.03, t (7.9)	139.9	2′	6.89, s	130.4
3′		131.5	3′		146.0
4′	2.35, m	34.8	4′	2.52, t (7.3)	35.2
5′	2.25, m	27.0	5′	2.39, q (7.3)	26.9
6′	5.26, t (7.4)	125.8	6′	5.35, t (7.3)	127.3
7′		139.7	7′		140.9
8′	2.16, overlap	34.2	8′	2.24, overlap	35.4
9′	2.16, overlap	26.4	9′	2.24, overlap	27.4
10′	5.51, t (6.1)	129.0	10′	5.52, br s	130.1
11′		130.4	11′		131.8
12′	4.54, s	69.4	12′	4.54, s	71.0
13′	1.67, s	13.3	13′	1.67, s	14.1
14′	4.10, s	58.9	14′	4.13, s	60.0
15′		169.5	15′		170.1
1′′		159.7	1′′		161.3
2′′, 6′′	6.89, d (8.6)	116.7	2′′, 6′′	6.78, d (8.6)	116.8
3′′, 5′′	7.55, d (8.6)	130.1	3′′, 5′′	7.45, d (8.7)	131.2
4′′		126.2 *^c^*	4′′		127.1
7′′	7.62, d (16.0)	144.5	7′′	7.60, d (15.9)	146.6
8′′	6.38, d (16.0)	114.7	8′′	6.32, d (15.9)	115.2
9′′		166.5	9′′		169.1
			-OCH_3_	3.66, s	52.7

*^a^* Recorded at 500 MHz for ^1^H and 125 MHz for ^13^C-NMR in acetone-*d*_6_. *^b^* Recorded at 500 MHz for ^1^H and 125 MHz for^13^C-NMR in methanol-*d*_4_. *^c^* Signals with the same symbol might be interchangeable.

**Table 3:** ^1^H- and ^13^C-NMR data of **5**–**7**.

No.	5 *^a^*		No.	6 *^a^*		No.	7 *^b^*	
	δ_H_ (*J* in Hz)	δ_C_		δ_H_ (*J* in Hz)	δ_C_		δ_H_ (*J* in Hz)	δ_C_
1		149.0	1		149.0	1		157.2
2		123.3	2		123.3	2		121.4
3	6.46, d (2.9)	113.4	3	6.47, d (2.9)	113.4	3	7.15, d (3.0)	115.8
4		151.4	4		151.4	4		150.8
5	6.61, dd (8.7, 2.9)	117.3	5	6.61, dd (8.7, 2.9)	117.3	5	7.04, dd (9.0, 3.0)	126.4
6	6.68, d (8.7)	117.3	6	6.67, d (8.7)	117.3	6	6.84, d (9.0)	119.8
1′	6.23, d (1.5)	79.8	1′	6.23, d (1.5)	79.9	1′		199.0
2′	7.37, d (1.5)	151.4	2′	7.37, d (1.5)	151.4	2′	7.63, s	131.3
3′		132.8	3′		132.9 *^c^*	3′		148.5
4′	2.41, m	26.1	4′	2.39, m	26.2 *^d^*	4′	2.66, m	29.8
5′	2.46, m	26.4	5′	2.45, m	26.4 *^d^*	5′	2.28, m	28.2
6′	5.58, t (6.9)	129.2	6′	5.58, t (7.0)	129.3	6′	5.22, t (7.6)	127.4
7′		142.6	7′		142.5	7′		137.2
8′	4.18, t (6.5)	75.2	8′	4.18, t (6.5)	75.3	8′	4.03, s	61.2
9′	2.32, m	35.4	9′	2.33, m	35.4	9′	1.62, s	21.3
10′	5.53, t (6.5)	126.6	10′	5.54, t (6.5)	126.6	10′		171.5
11′		133.1	11′		133.1*^c^*			
12′	4.56, s	70.8	12′	4.56, s	70.8			
13′	1.69, s	14.4	13′	1.69, s	14.4			
14′	4.19, d (12.2)	58.1	14′	4.19, d (12.2)	58.1			
	4.11, d (12.2)			4.13, d (12.2)				
15′		176.7	15′		176.7			
1′′		161.2	1′′		161.3			
2′′, 6′′	6.80, d (8.6)	116.8	2′′, 6′′	6.80, d (8.6)	116.8			
3′′, 5′′	7.45, d (8.6)	131.2	3′′, 5′′	7.45, d (8.6)	131.2			
4′′		127.1	4′′		127.1			
7′′	7.61, d (15.9)	146.6	7′′	7.61, d (15.9)	146.6			
8′′	6.33, d (15.9)	115.2	8′′	6.32, d (15.9)	115.2			
9′′		169.1	9′′		169.1			

*^a^* Recorded at 500 MHz for ^1^H and 125 MHz for ^13^C-NMR in methanol-*d*_4_. *^b^* Recorded at 500 MHz for ^1^H and 150 MHz for ^13^C-NMR in methanol-*d*_4_. *^c^*^,^*^d^* Signals with the same symbol might be interchangeable.

**Table 4:** IC_50_ values for **1**–**7** and 5-FU on different cell lines.

Compound	Cell Lines (IC_50_, μM)			
	BGC-823	KYSE30	A549	HDF
(−)-1	>160	>160	>160	>160
(+)-1	>160	>160	>160	>160
(−)-2	>160	>160	>160	>160
(+)-2	>160	>160	>160	>160
3	>160	>160	>160	>160
4	>160	>160	72.13 ± 8.7 *	>160
5	59.2 ± 2.73 *	76.28 ± 1.93 *	>160	>160
6	64.25 ± 0.37 *	85.42 ± 2.82 *	>160	>160
7	>160	>160	>160	>160
5-FU	37.72 ± 1.87	8.43 ± 0.19	35.02 ± 2.42	51.07 ± 2.43

BGC-823: human gastric cancer cells. KYSE30: human esophageal cancer cells. A549: human lung cancer cells. HDF: normal human embryonic lung fibroblasts. * *p* < 0.05 (vs. 5-FU group).

## Supplementary Materials

The following are available online. Figures S1–S7: NMR spectra and HRESIMS of **1**, Figures S8–S14: NMR spectra and HRESIMS of **2**, Figures S15–S21: NMR spectra and HRESIMS of **3**, Figures S22–S28: NMR spectra and HRESIMS of **4**, Figures S29–S32: NMR spectra of Fr.7.3.6.6.2.5.3, Figures S33–S40: NMR and ECD spectra and HRESIMS of **5**, Figures S41–S48: NMR and ECD spectra and HRESIMS of **6**, Figures S49–S50: NMR spectra of **5a** and **5b**, Figures S51–S52: NMR spectra of **6a** and **6b**, Figures S53–S59: NMR spectra and HRESIMS of **7**, Figure S60: The lowest energy conformers of **1**, Figure S61: The calculated and experimental ECD spectra of **1** and **2**, Figure S62: The calculated model compounds **5c** and **6c**, Figure S63: The calculated and the experimental ECD spectra of **5** and **6**, Figure S64: Cytotoxic effects of **4**, **5**, **6** and 5-FU on human cancer cells and normal cells Cells, Table S1: Extracted heats and weighting factors of the optimized conformers of **1**, Table S2: The Cartesian coordinates of the lowest energy conformers for **1**, Table S3: Standard orientation of **5c** in MeOH, Table S4: Standard orientation of **6c** in MeOH.
