# Supplementary material for: Meroterpenoids from the Fungus Ganoderma sinensis and First Absolute Configuration Clarification of Zizhine H
Source: Molecules. 2019 Dec 31;25(1):158. doi: 10.3390/molecules25010158 (PMC6983046; doi:10.3390/molecules25010158)
Supplement: Supplementary file 1 [file molecules-25-00158-s001.pdf]

Supplementary Materials: Cytotoxicity Meroterpenoids from the Fungus *Ganoderma sinensis* and First Absolute Configuration Clarification of Zizhine H

Yan-Jiao Yin, Dan-Ling Huang, Bin Qiu, Dan Cai, Jiao-Jiao Zhang, Shao-Xiang Wang, Da-Peng Qin, and Yong-Xian Cheng

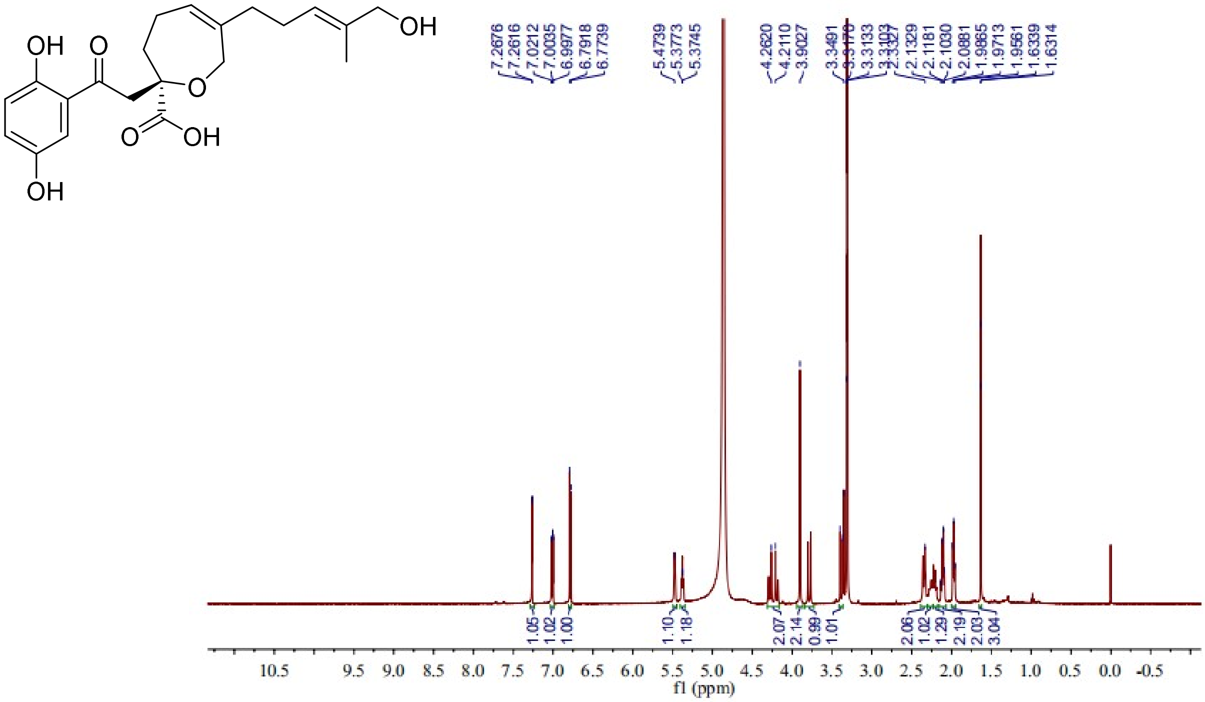

Figure S1. ¹H NMR spectrum of **1** in methanol-d_4.

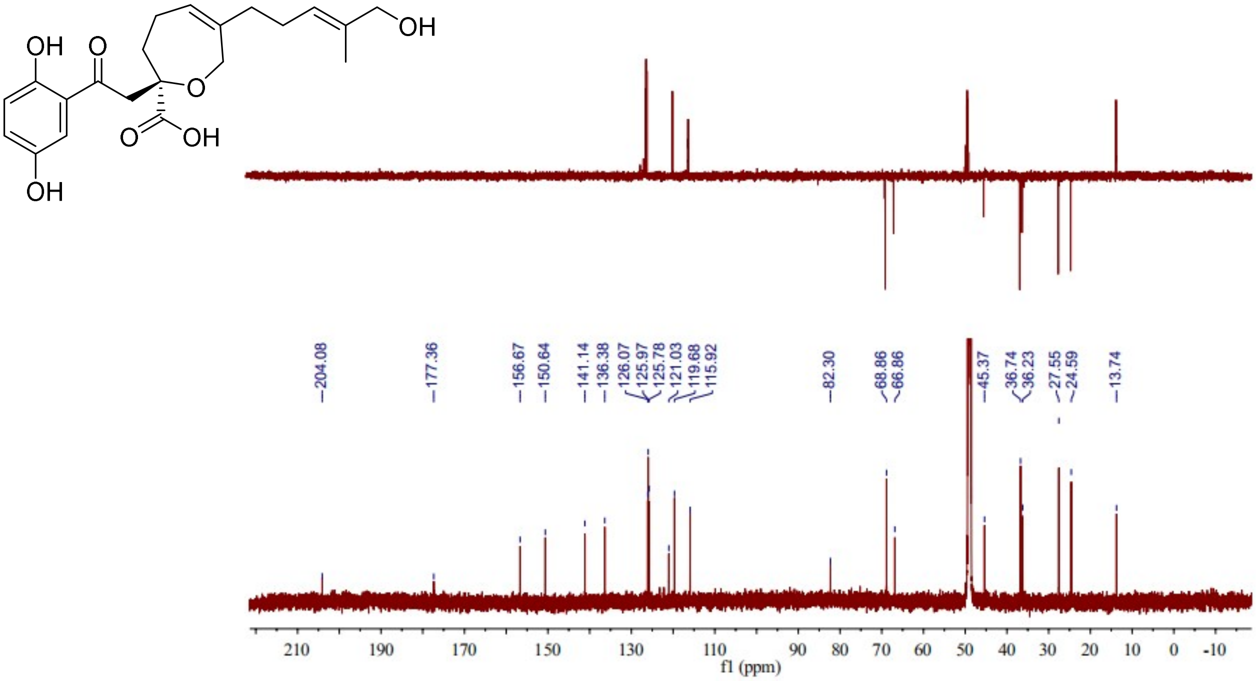

Figure S2. ^{13}C NMR and DEPT spectra of **1** in methanol-d_4.

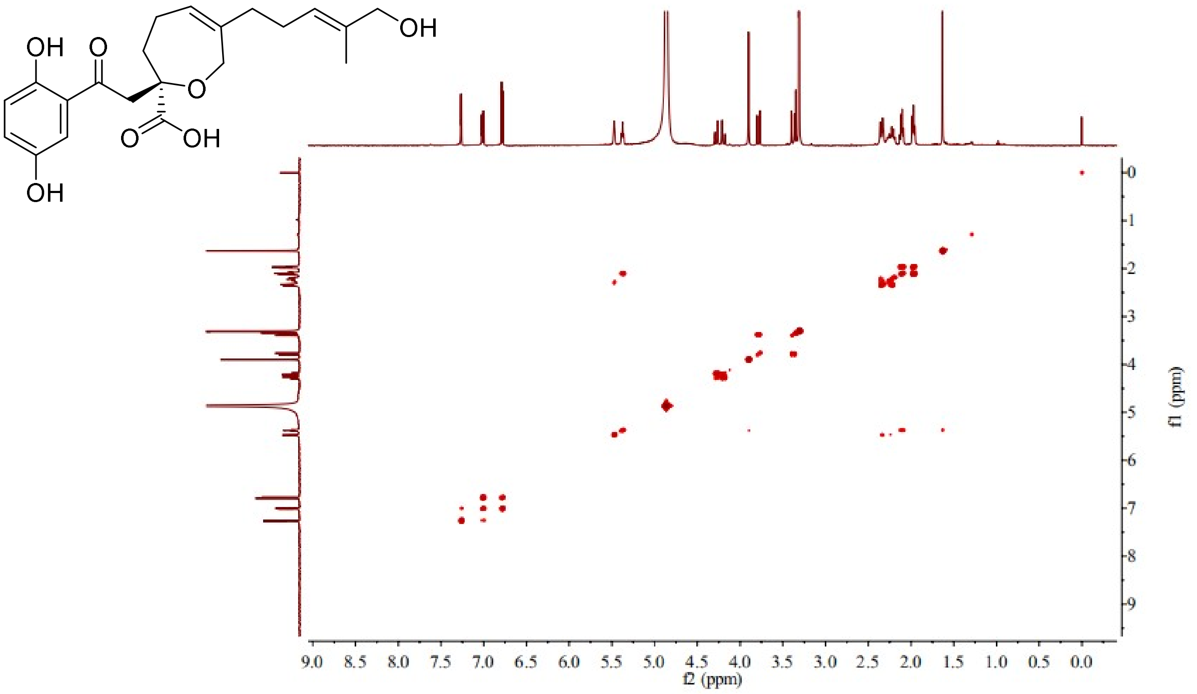

Figure S3. ^{1}H-^{1}H COSY spectrum of **1** in methanol-d_4.

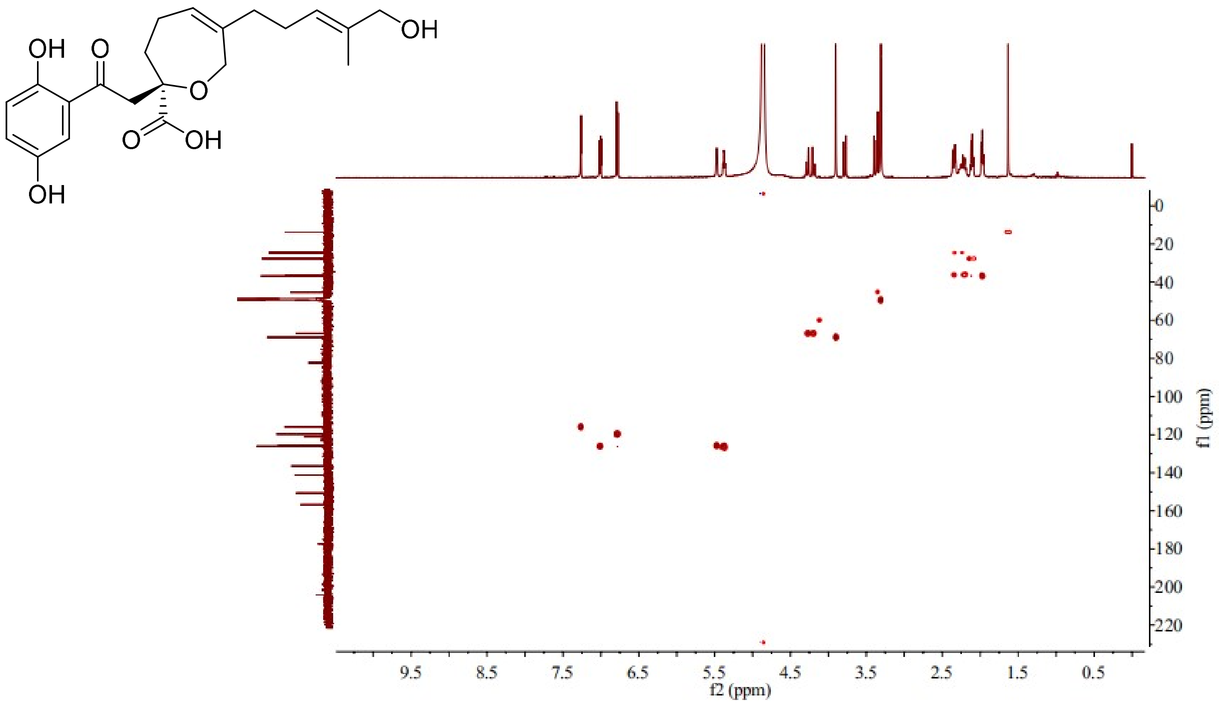

Figure S4. HSQC spectrum of **1** in methanol-d_4.

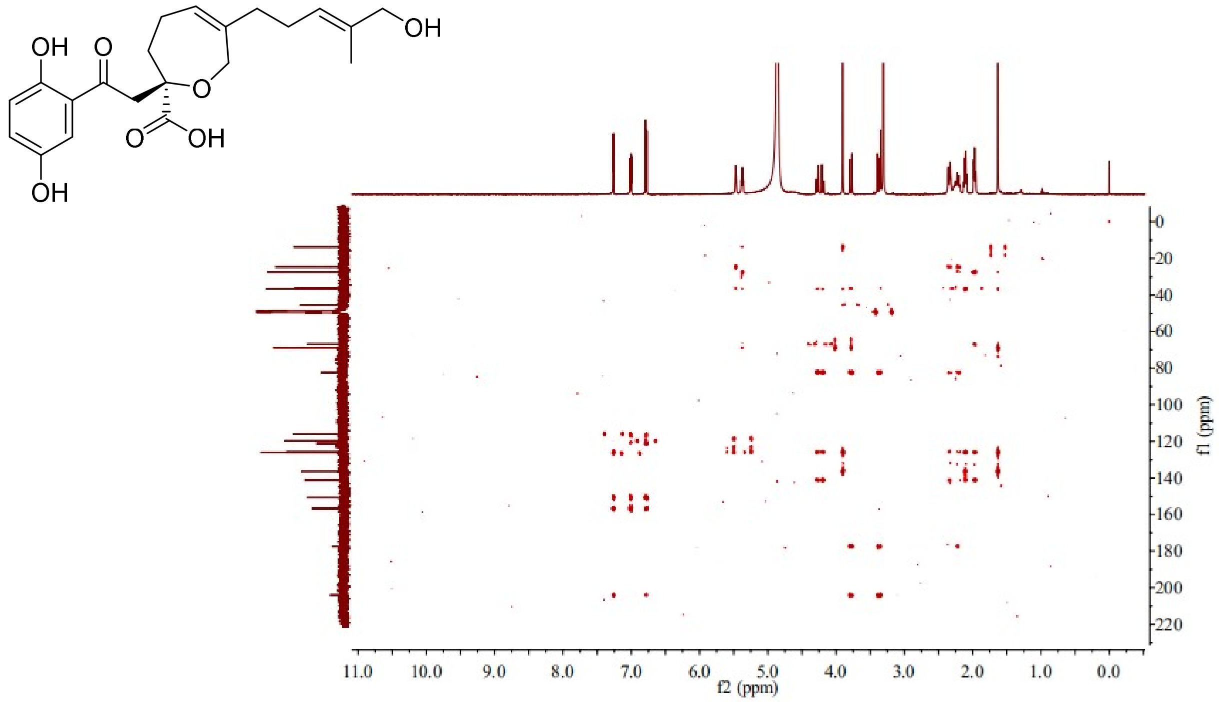

Figure S5. HMBC spectrum of **1** in methanol-d_4.

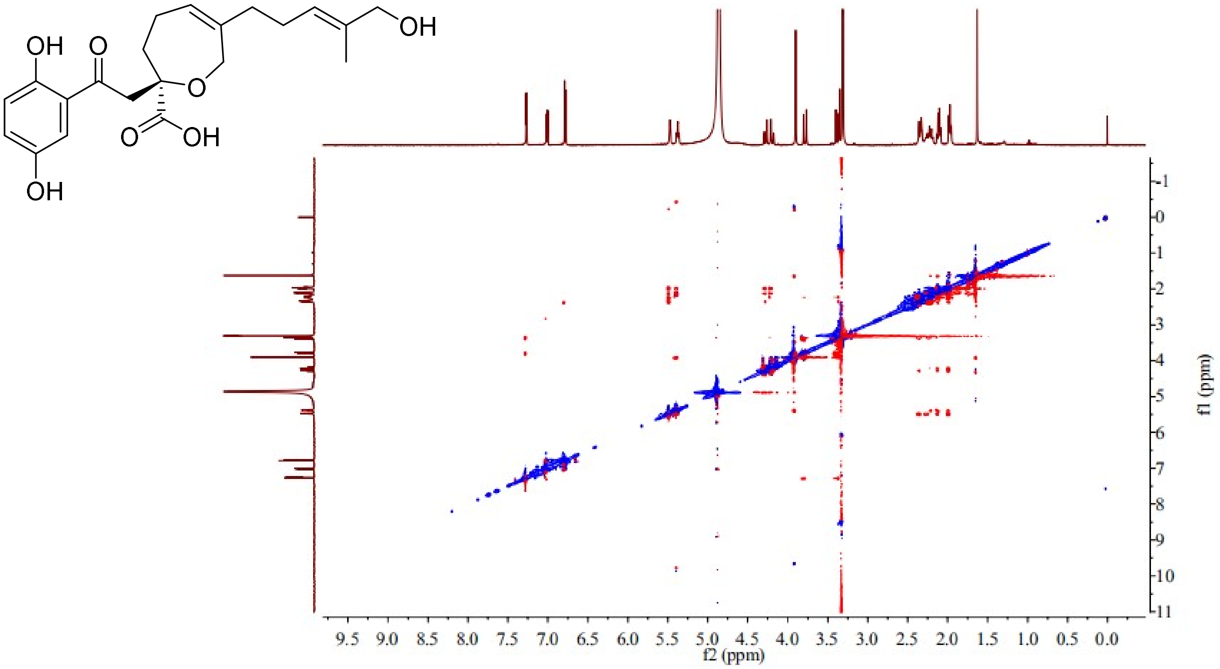

Figure S6. ROESY spectrum of **1** in methanol-d_4.

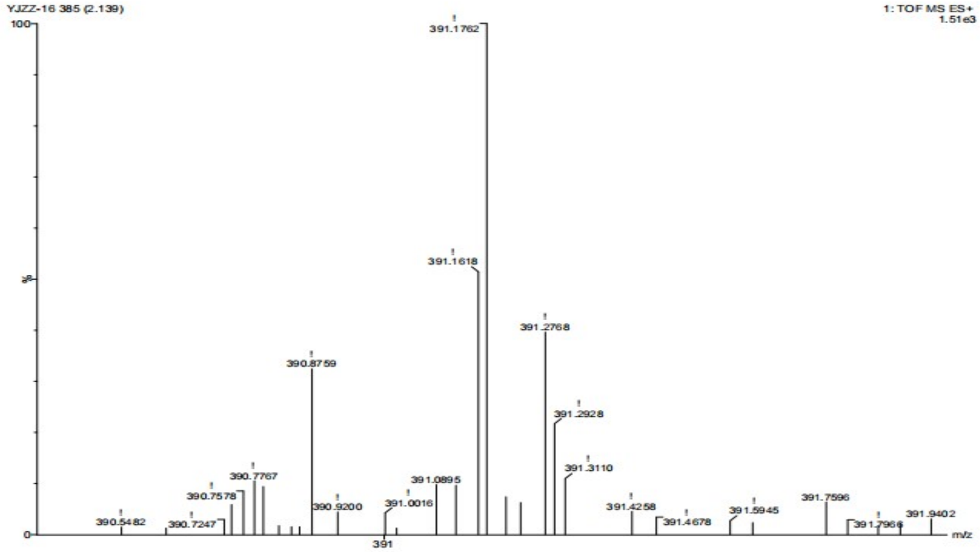

Single Mass Analysis
Tolerance = 10.0 PPM / DBE: min = -1.5, max = 50.0
Element prediction: Off
Number of isotope peaks used for i-FIT = 3

Monoisotopic Mass, Even Electron Ions
93 formula(e) evaluated with 1 results within limits (up to 50 closest results for each mass)
Elements Used:
C: 21-21 H: 0-50 N: 0-4 O: 1-10 Na: 0-1
YJZZ-16 385 (2.139)
1: TOF MS ES+

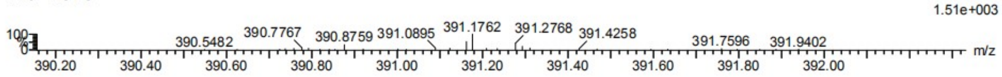

Minimum:				-1.5				
Maximum:		20.0	10.0	50.0				
Mass	Calc. Mass	mDa	PPM	DBE	i-FIT	Norm	Conf(%)	Formula
391.1762	391.1757	0.5	1.3	8.5	147.2	n/a	n/a	C21 H27 O7

Figure S7. HRESIMS of **1**.

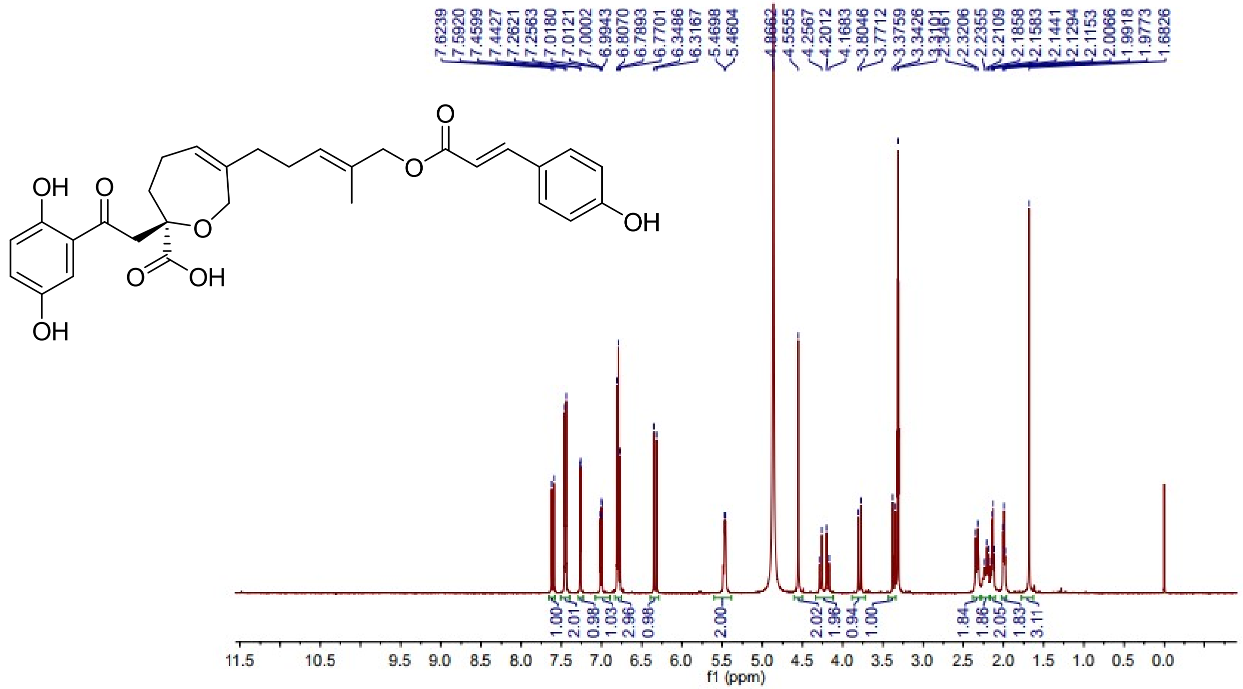

Figure S8. ^{1}H NMR spectrum of **2** in methanol-d_4.

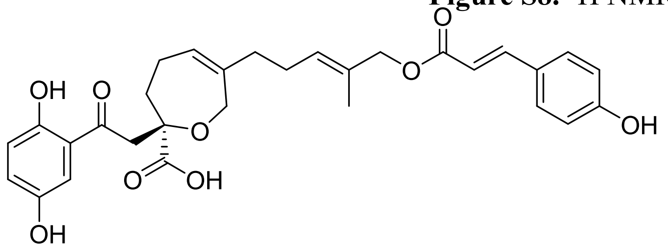

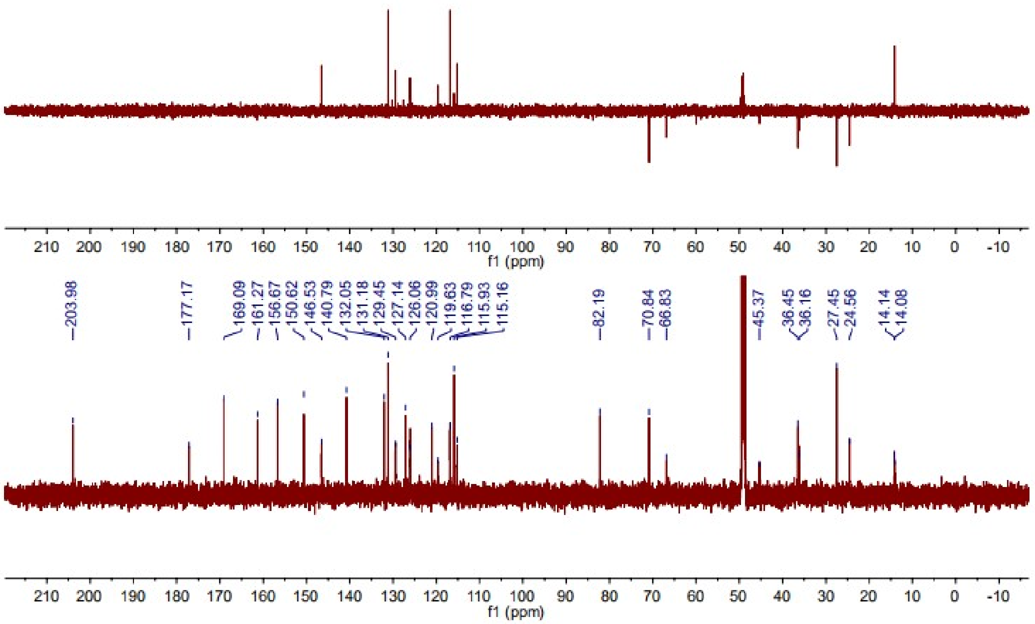

Figure S9. ^{13}C NMR and DEPT spectra of **2** in methanol-d_4.

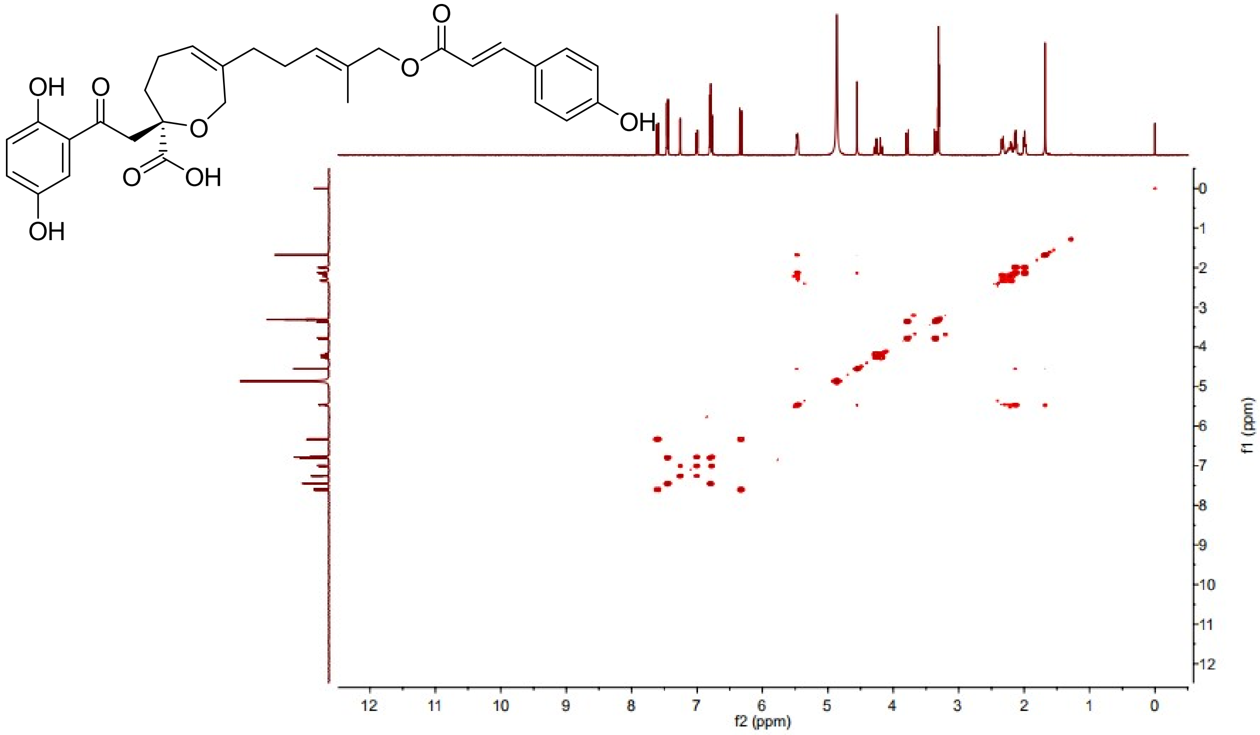

Figure S10. ^{1}H–^{1}H COSY spectrum of **2** in methanol-d_4.

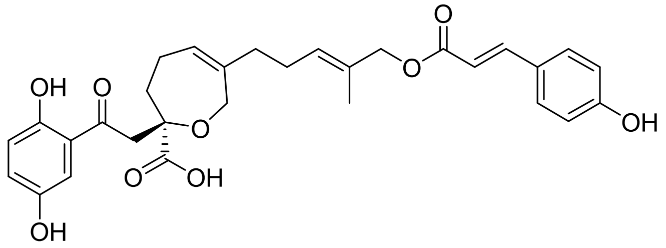

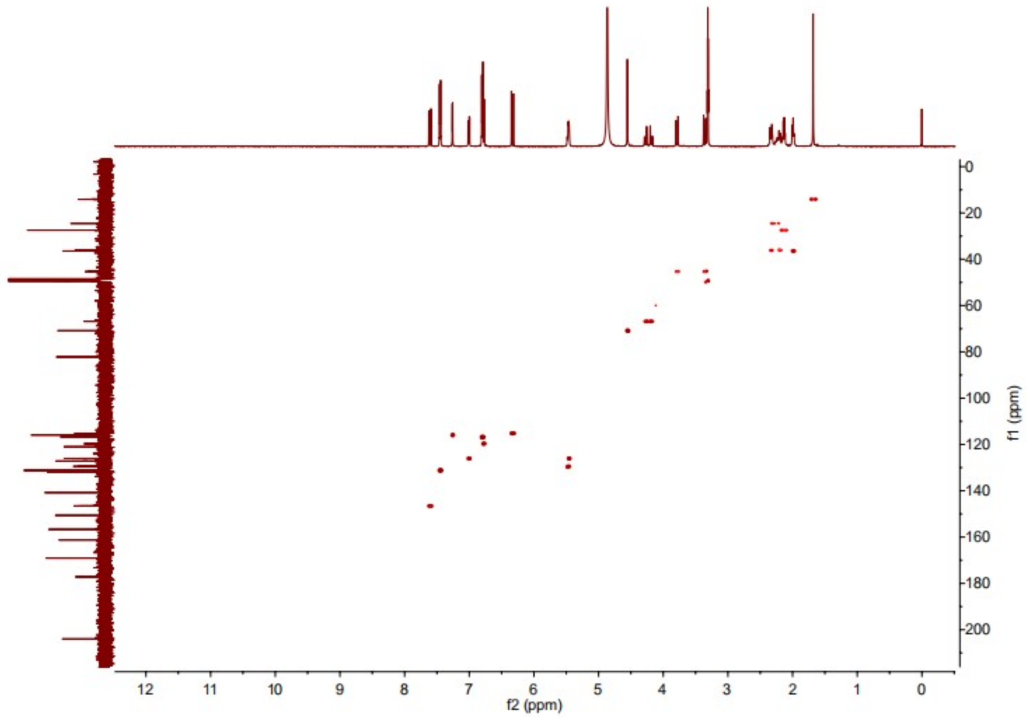

Figure S11. HSQC spectrum of **2** in methanol-d_4.

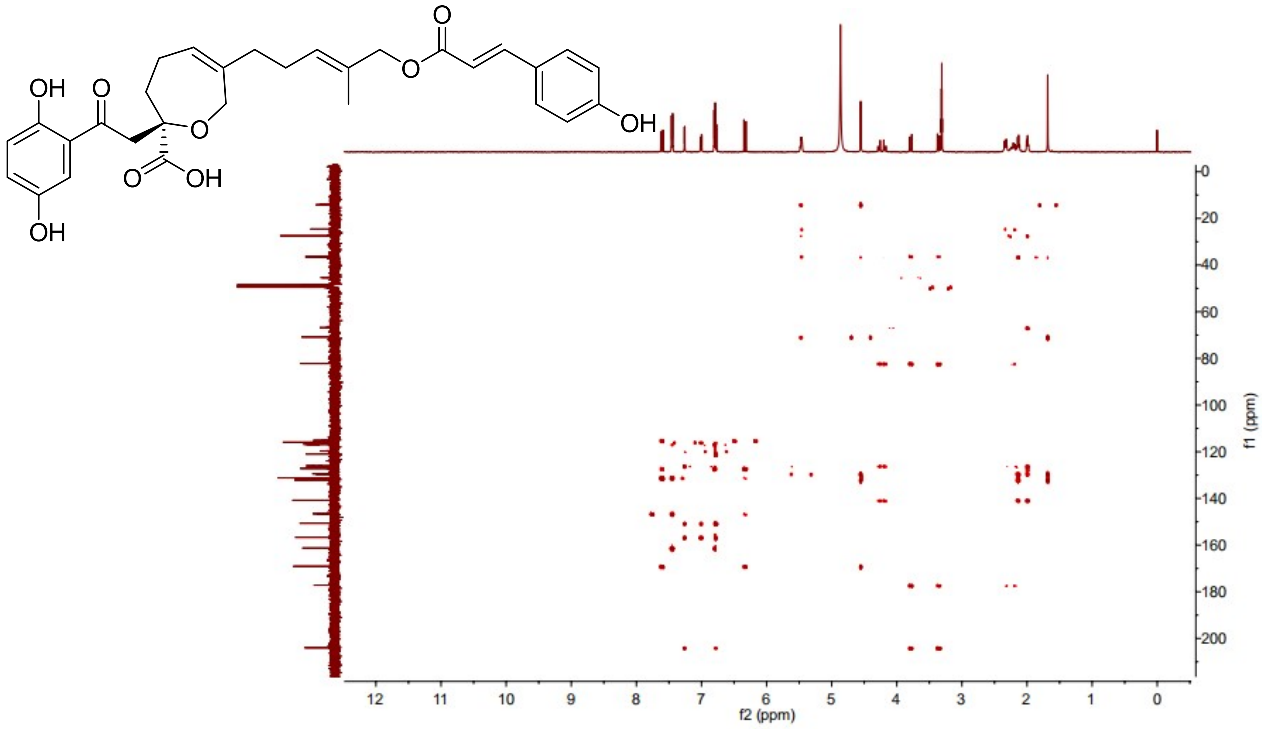

Figure S12. HMBC spectrum of **2** in methanol-d_4.

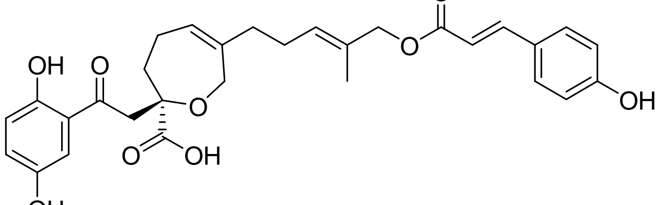

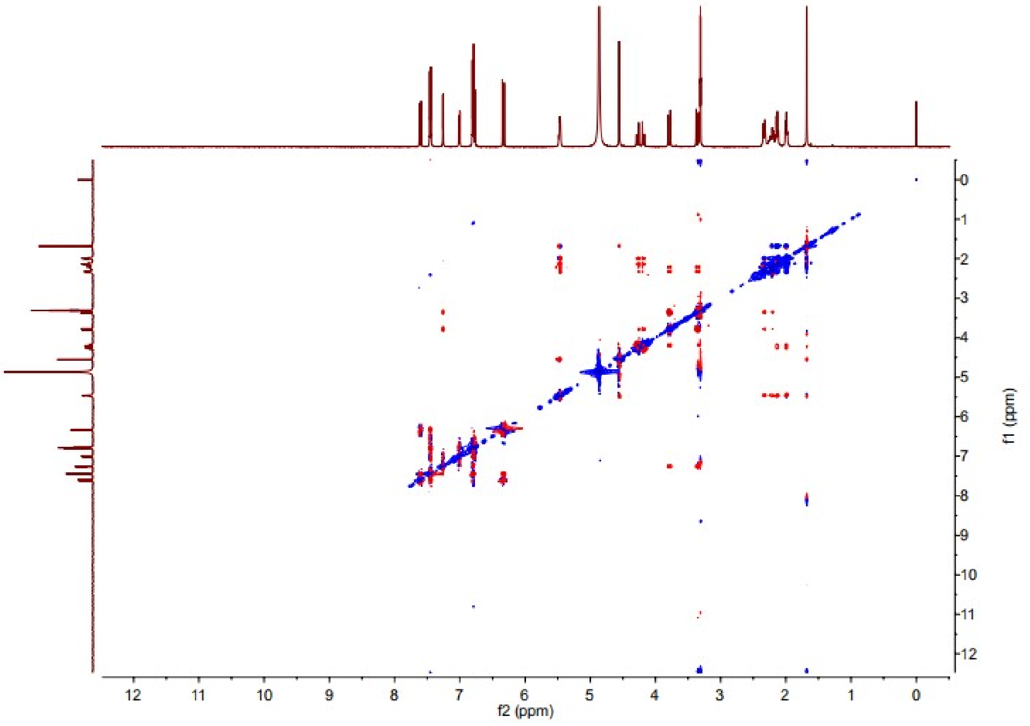

Figure S13. ROESY spectrum of **2** in methanol-d_4.

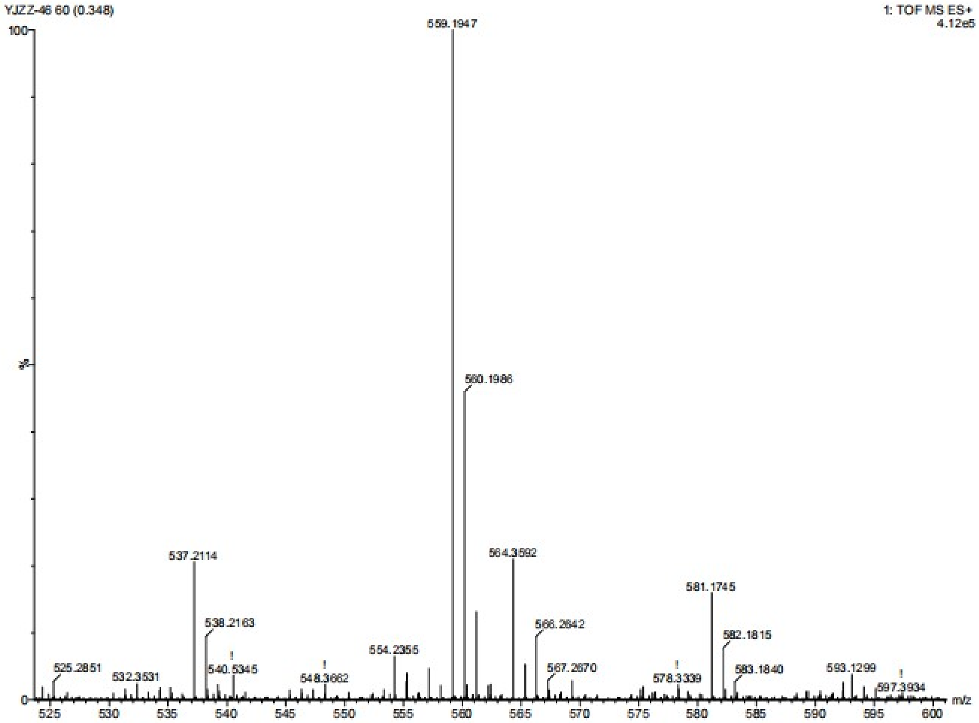

Single Mass Analysis
Tolerance = 10.0 PPM / DBE: min = -1.5, max = 50.0
Element prediction: Off
Number of isotope peaks used for i-FIT = 3

Monoisotopic Mass, Even Electron Ions
100 formula(e) evaluated with 1 results within limits (up to 50 closest results for each mass)
Elements Used:
C: 30-30 H: 0-50 N: 0-5 O: 1-10 Na: 0-1
YJZZ-46 60 (0.348)
1: TOF MS ES+

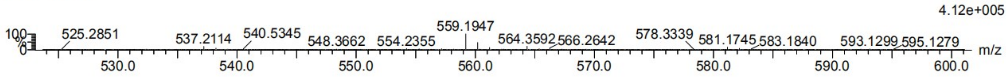

Minimum:				-1.5				
Maximum:		20.0	10.0	50.0				
Mass	Calc. Mass	mDa	PPM	DBE	i-FIT	Norm	Conf(%)	Formula
559.1947	559.1944	0.3	0.5	14.5	763.1	n/a	n/a	C30 H32 O9 Na

Figure S14. HRESIMS of **2**.

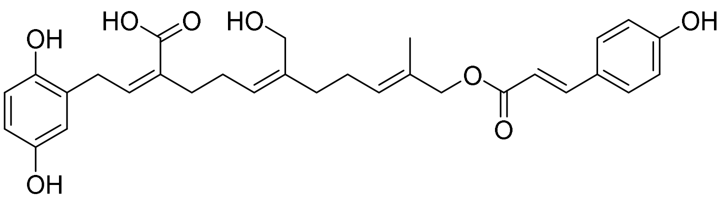

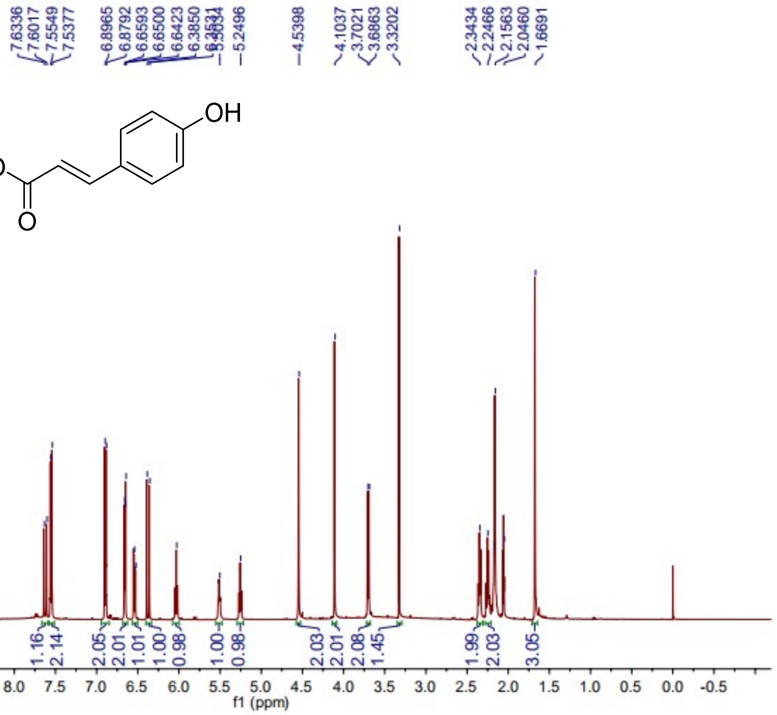

Figure S15. ^{1}H NMR spectrum of **3** in acetone-d_6.

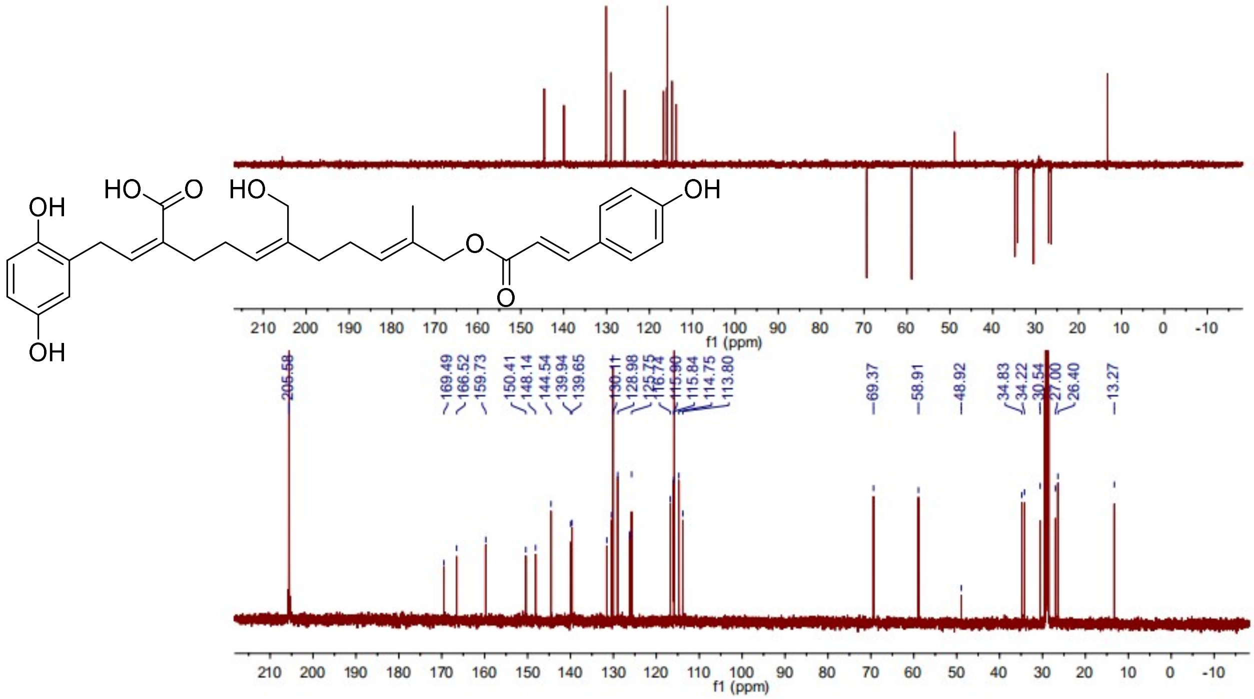

Figure S16. ^{13}C NMR and DEPT spectra of **3** in acetone-d_6.

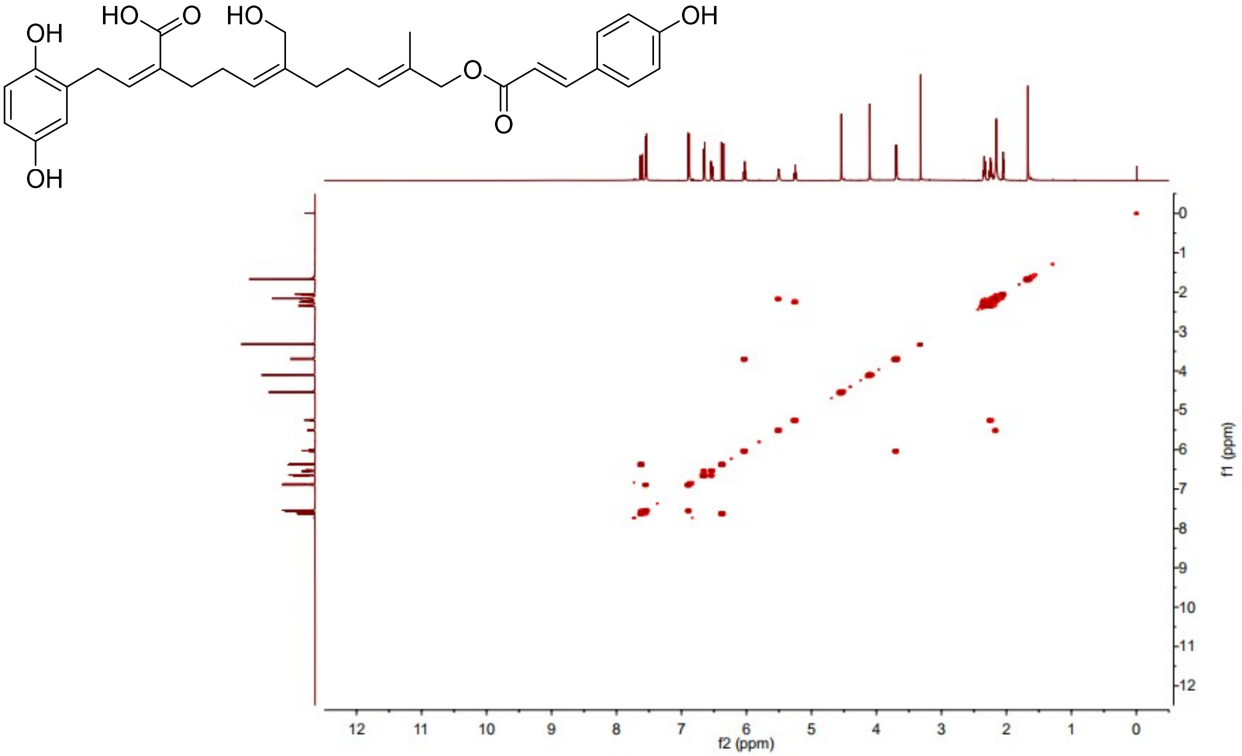

Figure S17. $^{1}H-^{1}H$ COSY spectra of **3** in acetone-d_6.

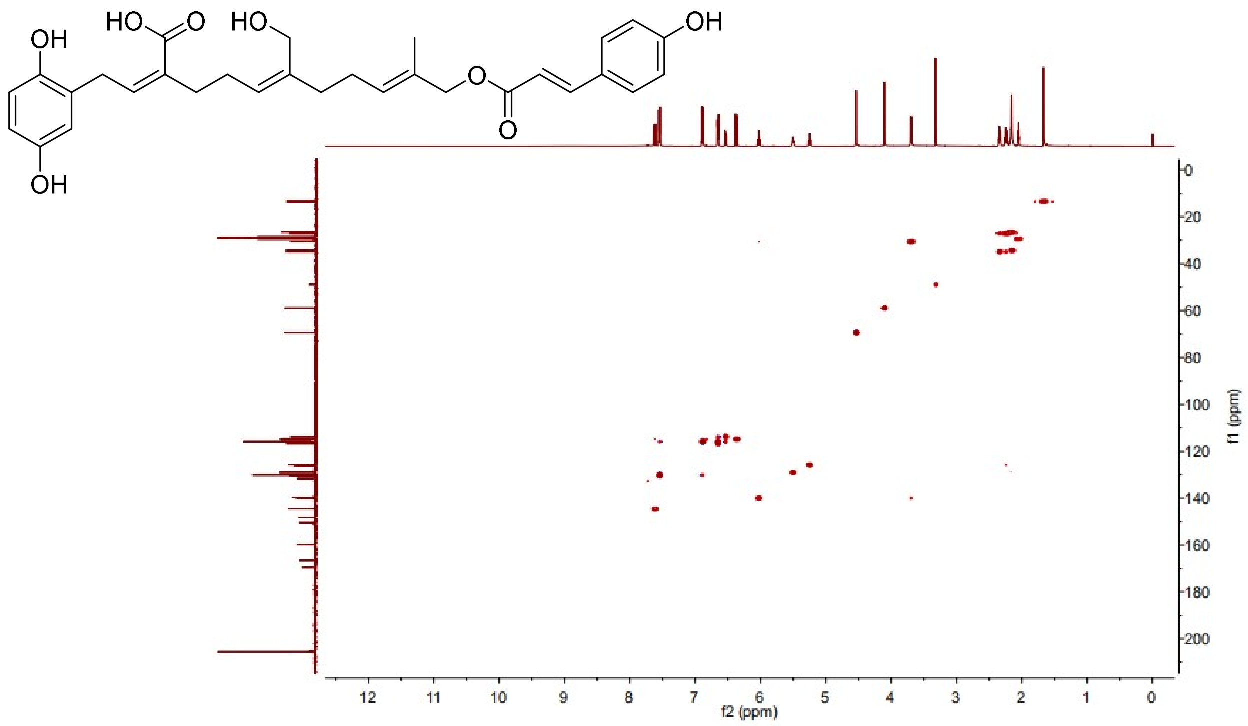

Figure S18. HSQC spectrum of **3** in acetone-d_6.

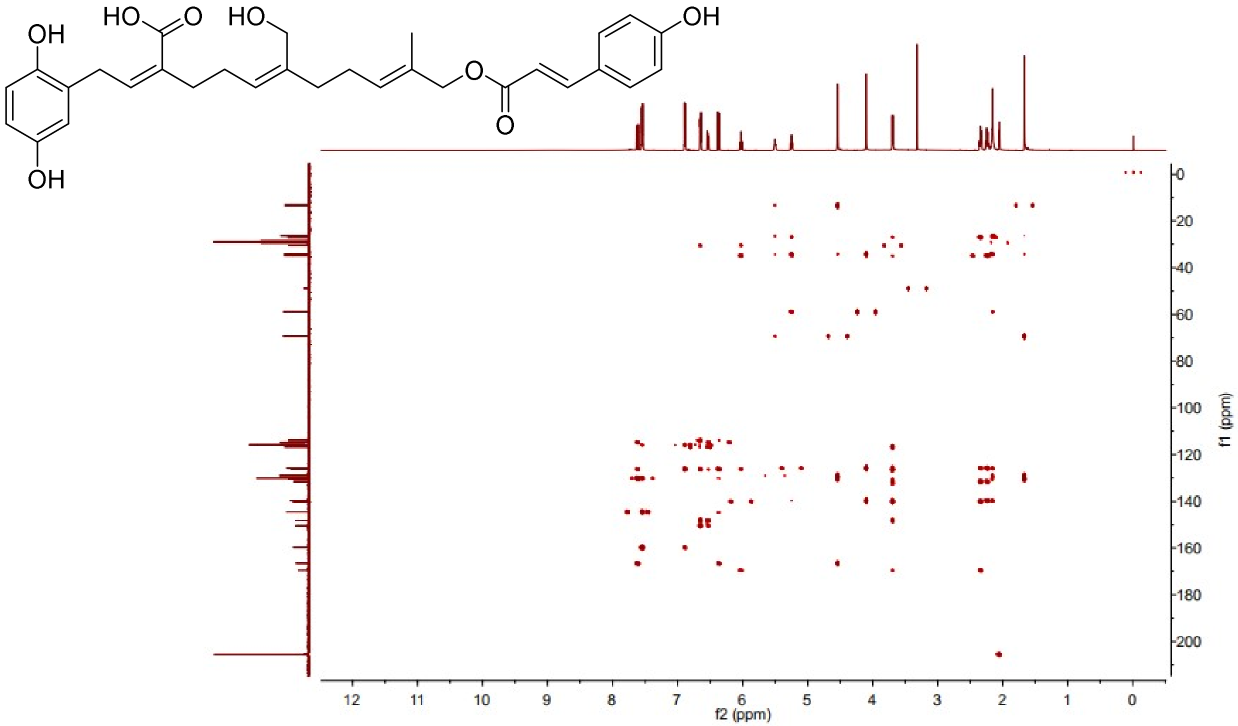

Figure S19. HMBC spectrum of **3** in acetone-d_6.

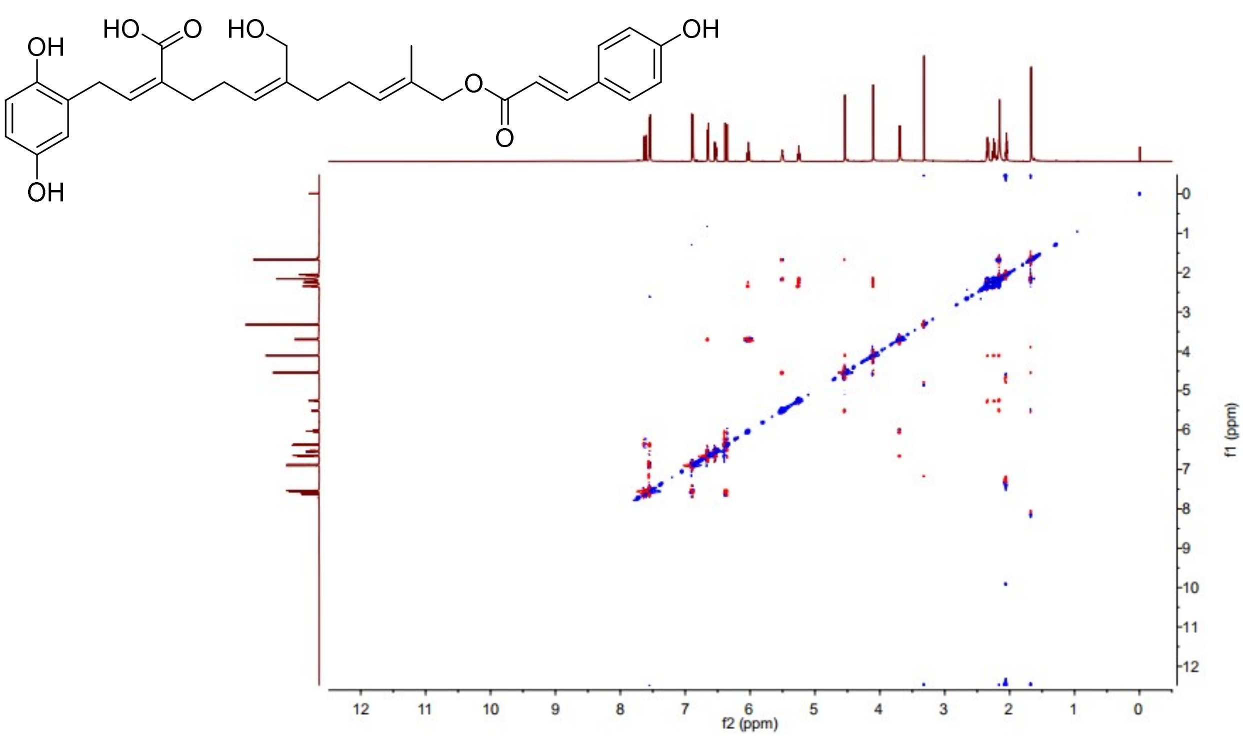

Figure S20. ROESY spectrum of **3** in acetone-d_6.

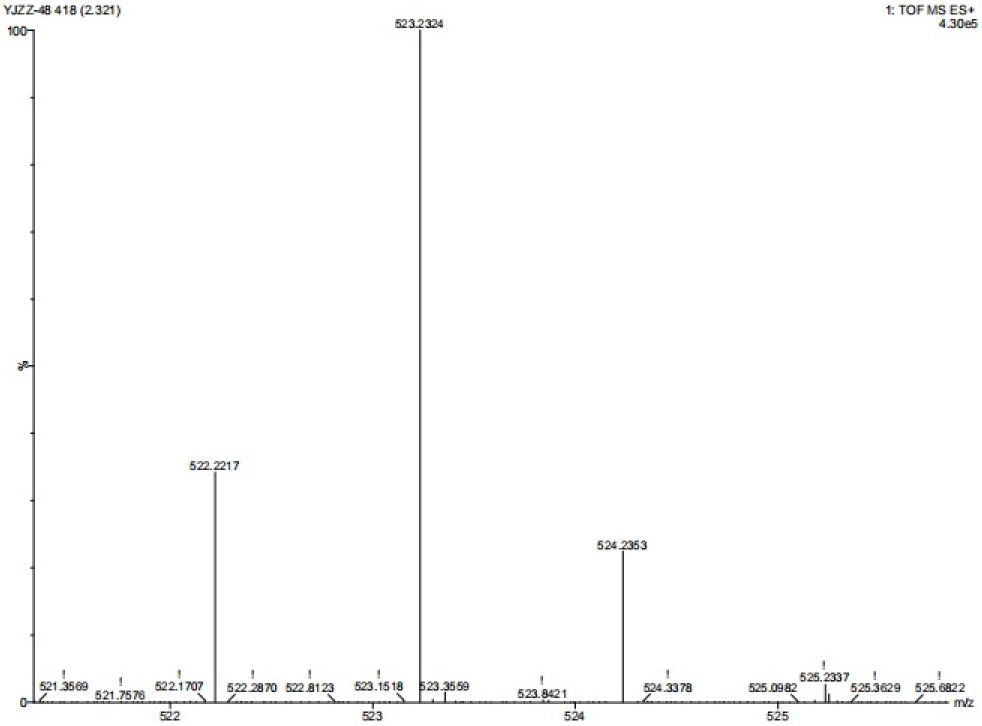

Single Mass Analysis
Tolerance = 10.0 PPM / DBE: min = -1.5, max = 50.0
Element prediction: Off
Number of isotope peaks used for i-FIT = 3

Monoisotopic Mass, Even Electron Ions
81 formula(e) evaluated with 1 results within limits (up to 50 closest results for each mass)
Elements Used:
C: 30-30 H: 0-50 N: 0-4 O: 1-10 Na: 0-1
YJZZ-48 418 (2.321)
1: TOF MS ES+

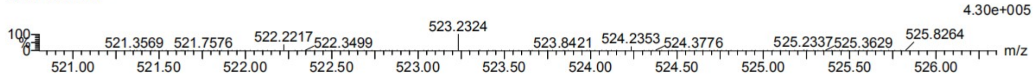

Minimum:				-1.5				
Maximum:		20.0	10.0	50.0				
Mass	Calc. Mass	mDa	PPM	DBE	i-FIT	Norm	Conf(%)	Formula
523.2324	523.2332	-0.8	-1.5	13.5	730.8	n/a	n/a	C30 H35 O8

Figure S21. HRESIMS of **3**.

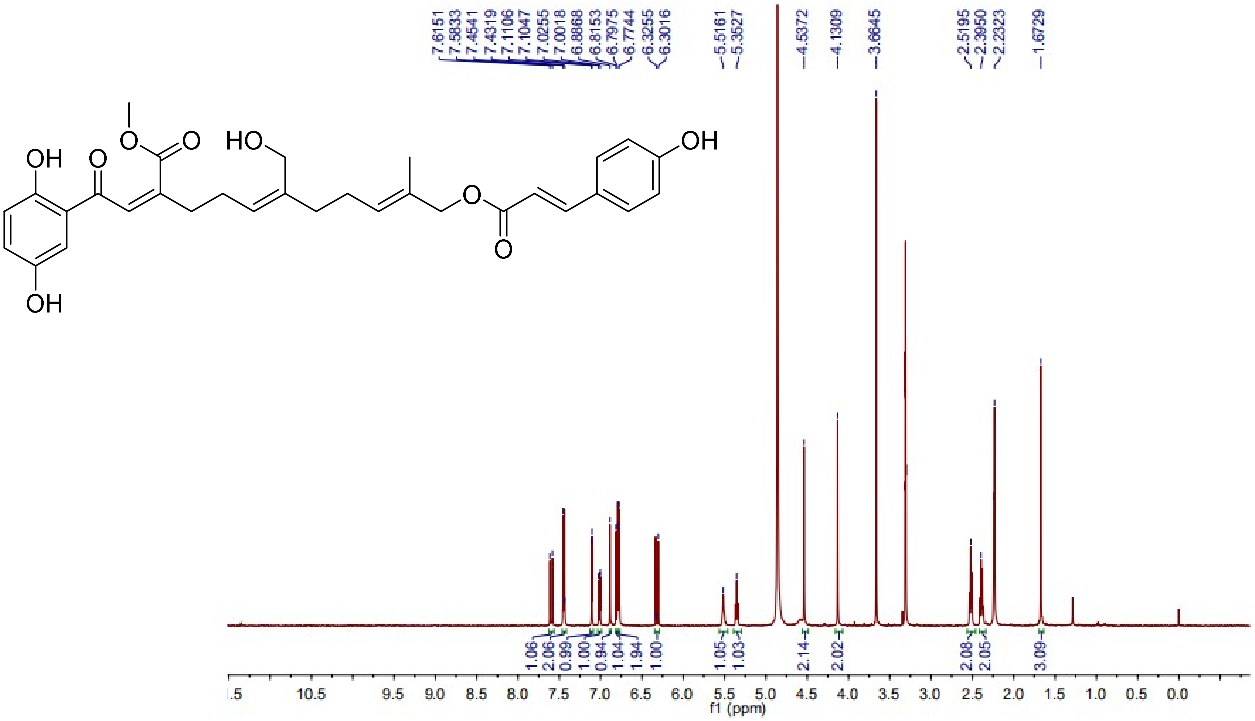

Figure S22. ^{1}H NMR spectrum of **4** in methanol-d_4.

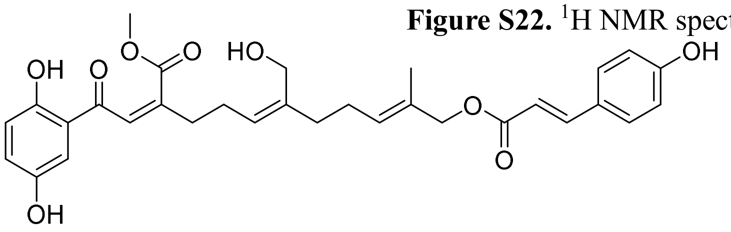

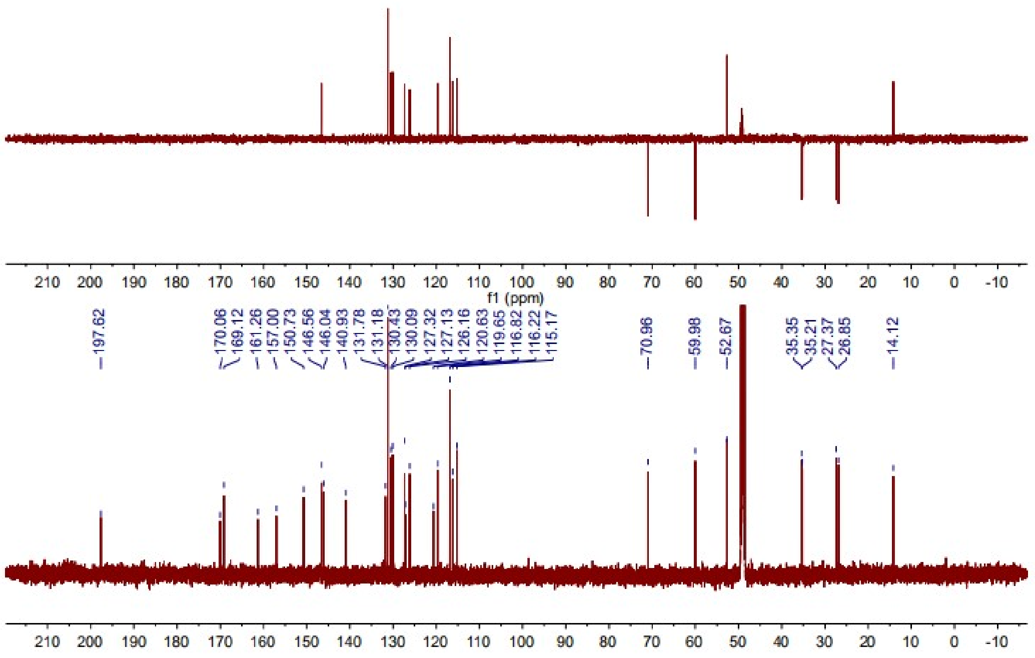

Figure S23. ^{13}C NMR and DEPT spectra of **4** in methanol-d_4.

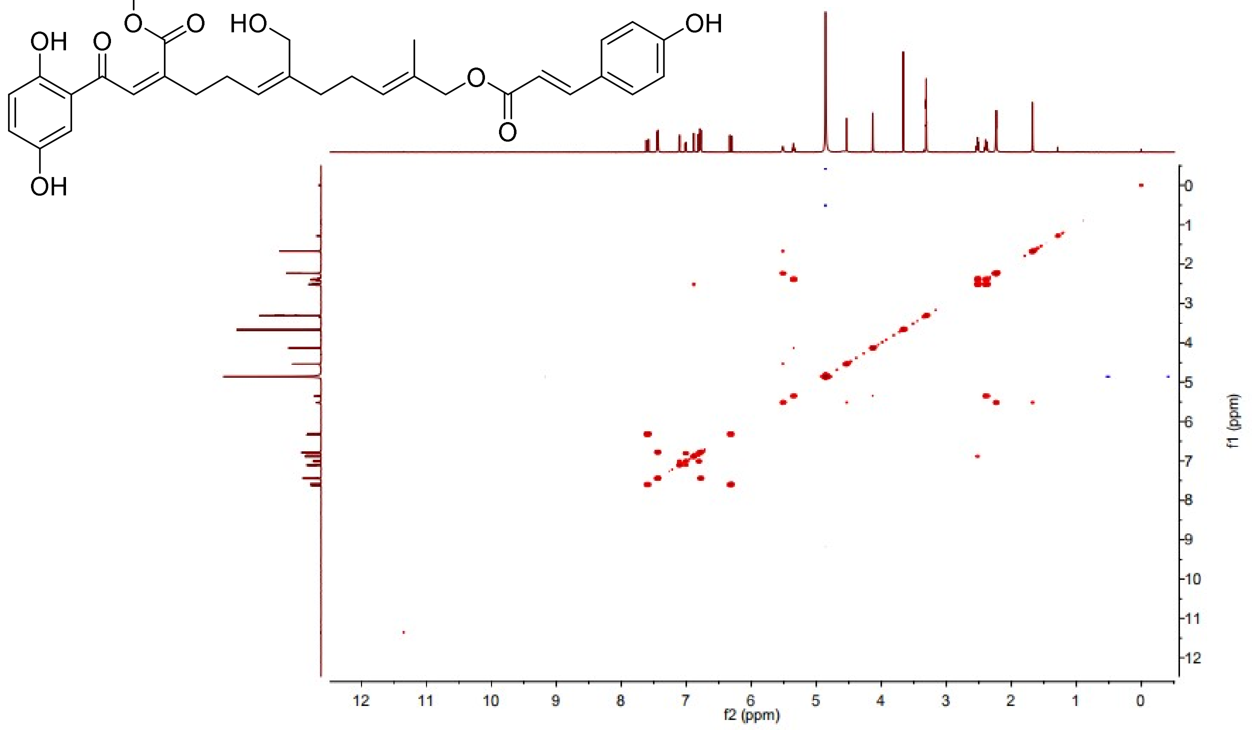

Figure S24. ^{1}H–^{1}H COSY spectrum of **4** in methanol-d_4.

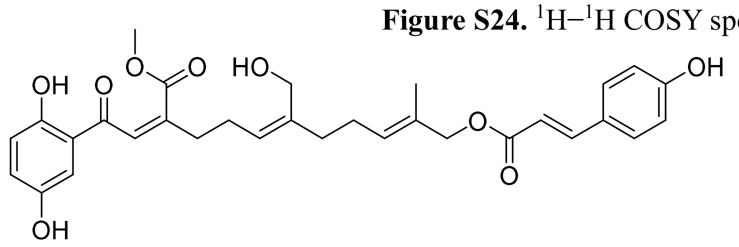

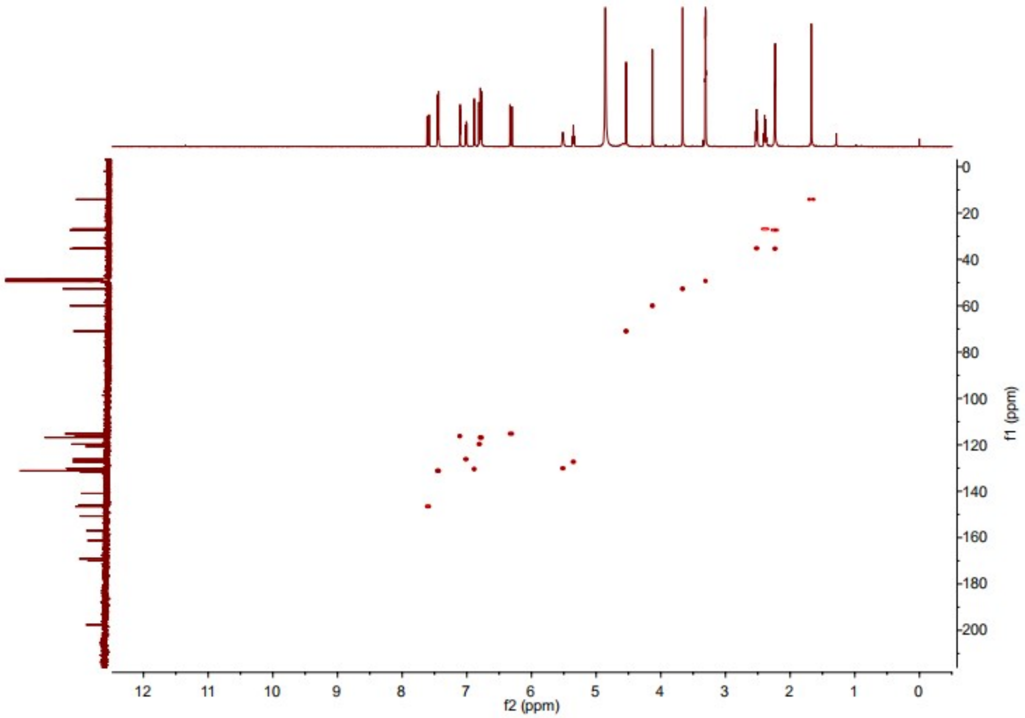

Figure S25. HSQC spectrum of **4** in methanol-d_4.

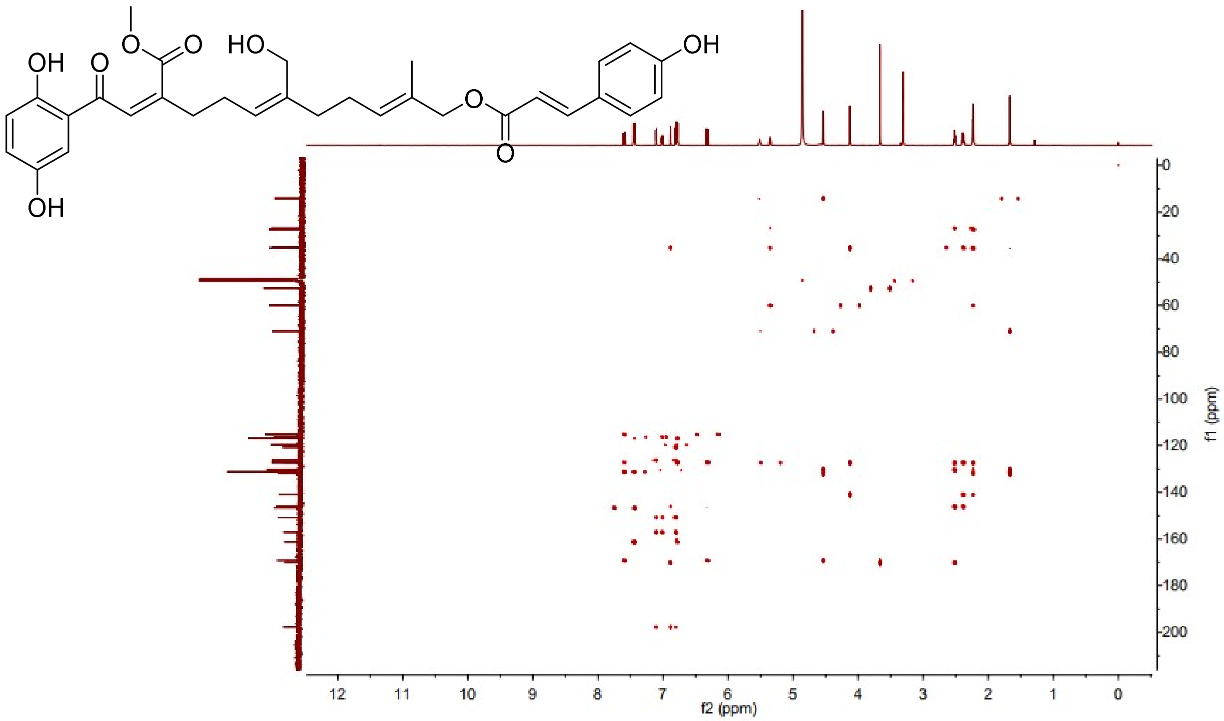

Figure S26. HMBC spectrum of **4** in methanol-d_4.

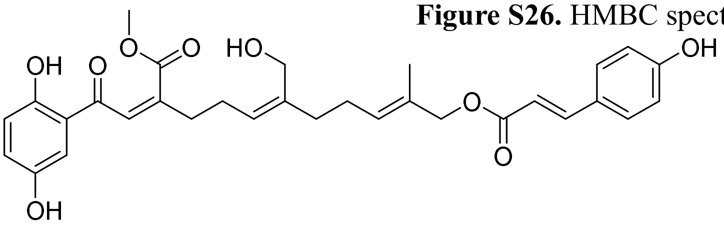

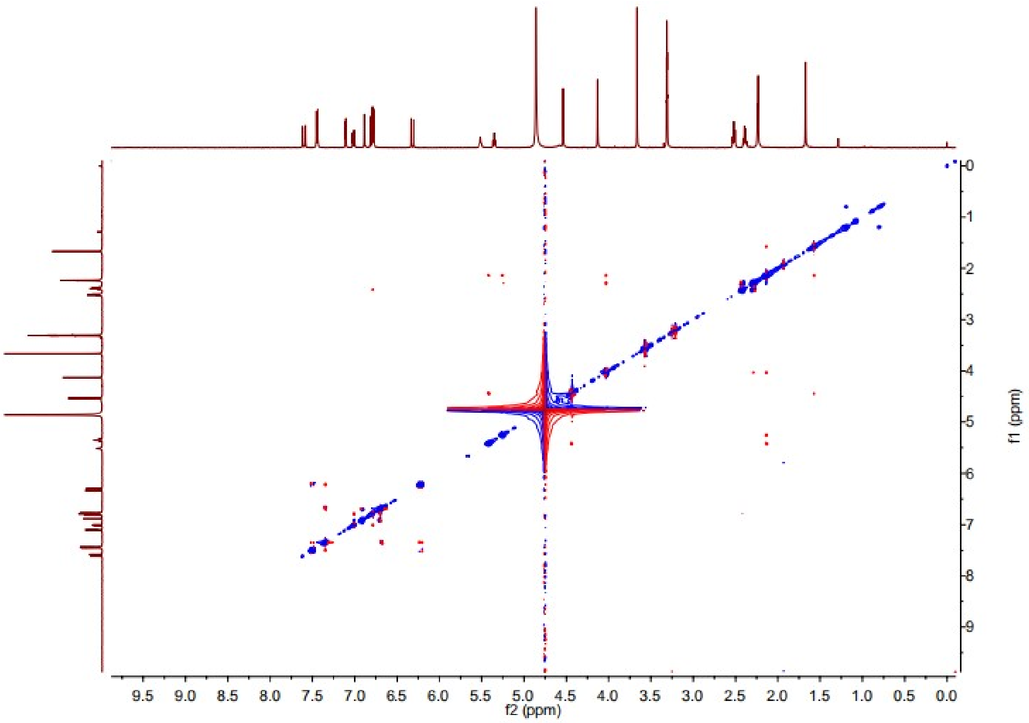

Figure S27. ROESY spectrum of **4** in methanol-*d*4.

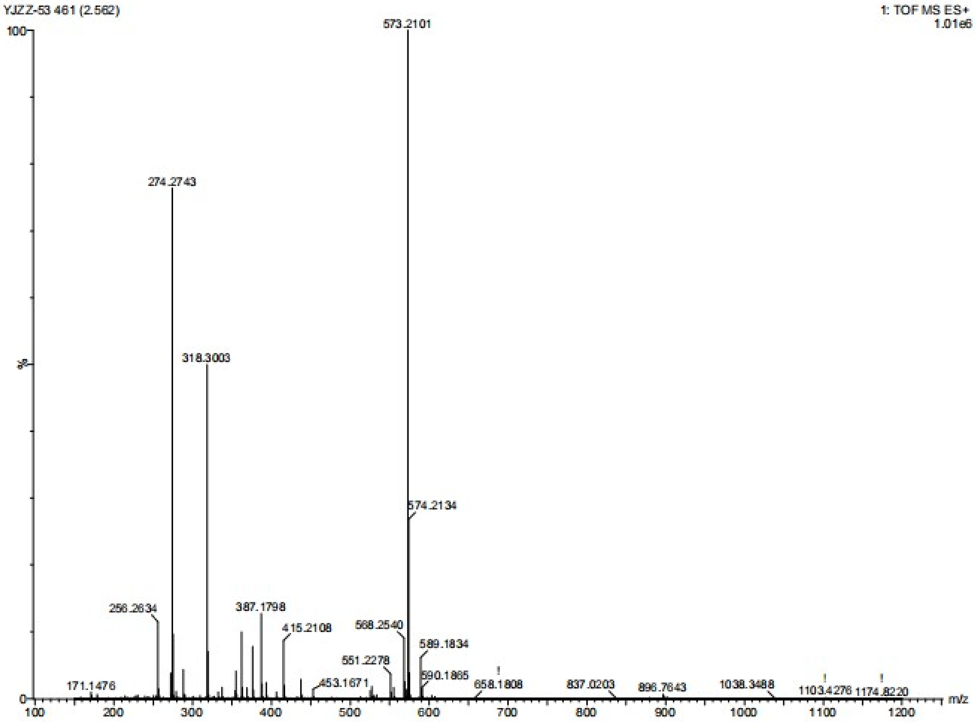

Single Mass Analysis
Tolerance = 10.0 PPM / DBE: min = -1.5, max = 50.0
Element prediction: Off
Number of isotope peaks used for i-FIT = 3

Monoisotopic Mass, Even Electron Ions
83 formula(e) evaluated with 1 results within limits (up to 50 closest results for each mass)
Elements Used:
C: 31-31 H: 0-50 N: 0-4 O: 1-10 Na: 0-1
YJZZ-53 461 (2.562)
1: TOF MS ES+

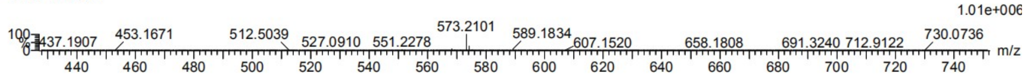

Minimum:				-1.5				
Maximum:		20.0	10.0	50.0				
Mass	Calc. Mass	mDa	PPM	DBE	i-FIT	Norm	Conf(%)	Formula
573.2101	573.2101	0.0	0.0	14.5	652.5	n/a	n/a	C31 H34 O9 Na

Figure S28. HRESIMS of **4**.

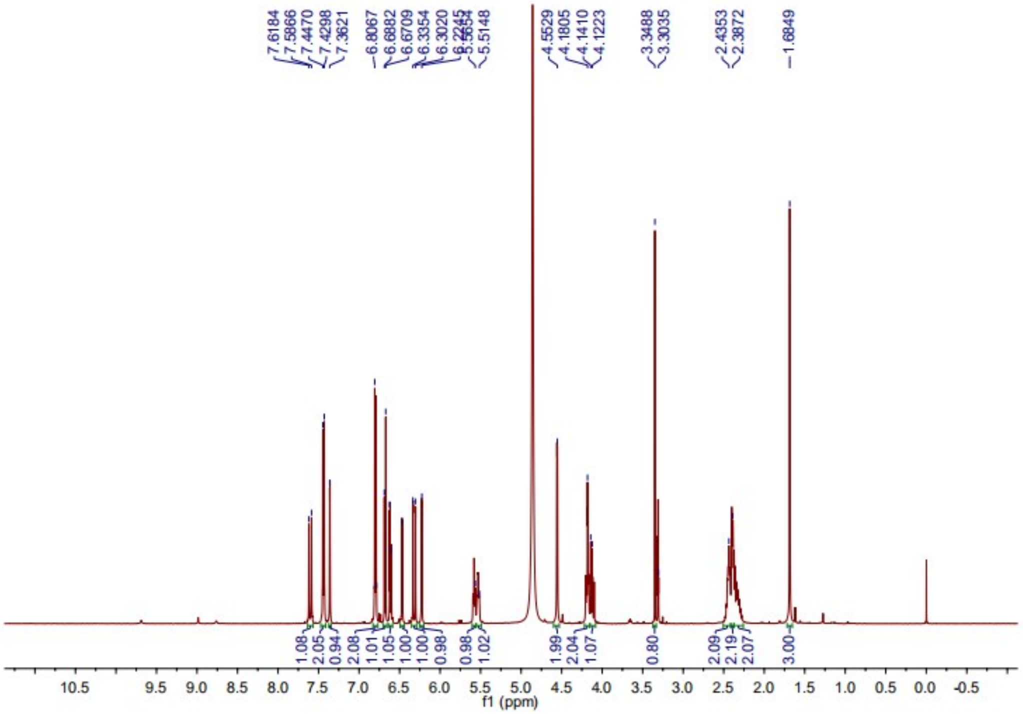

Figure S29. ^{1}H NMR spectrum of **Fr.7.3.6.6.2.5.3 (the mixture of 5 and 6)** in methanol-d_4.

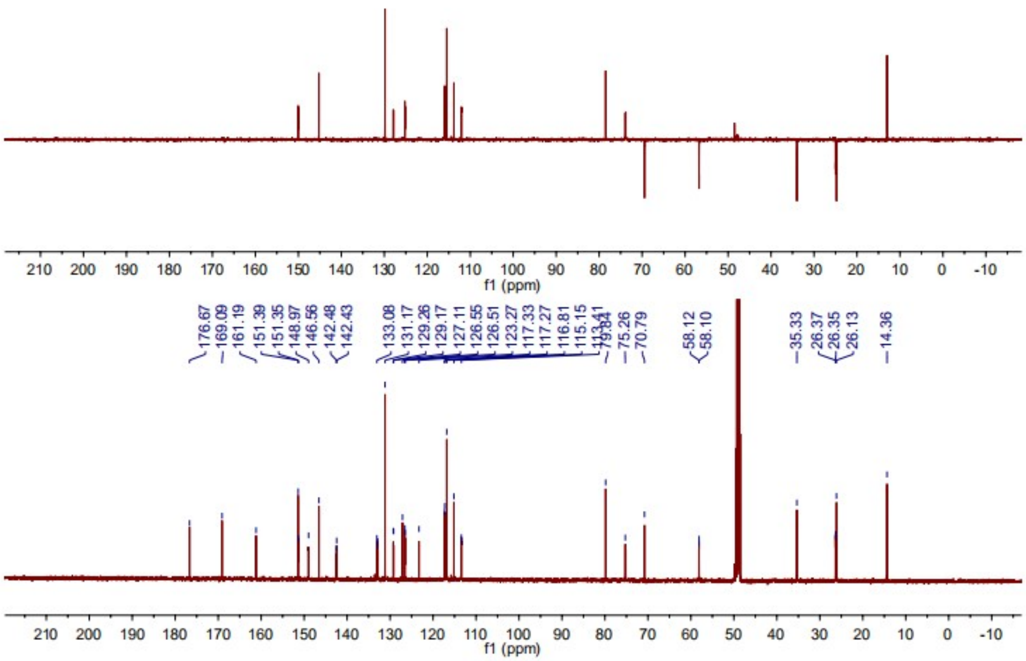

Figure S30. ^{13}C NMR and DEPT spectra of **Fr.7.3.6.6.2.5.3 (the mixture of 5 and 6)** in methanol-d_4.

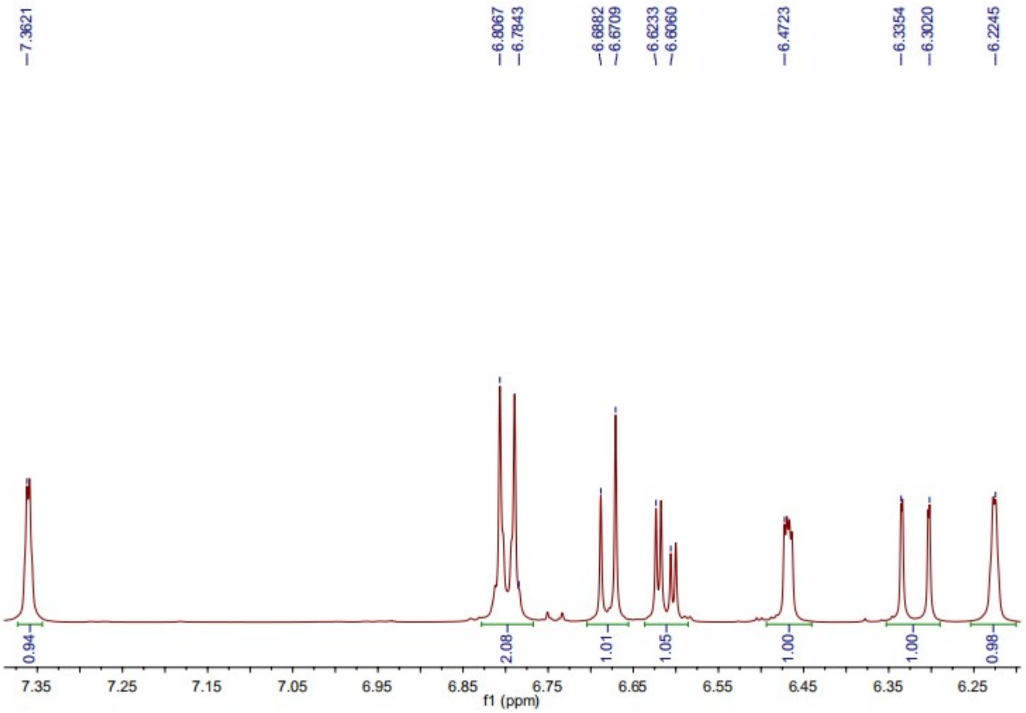

Figure S31. Enlarged ^{1}H NMR spectrum of **Fr.7.3.6.6.2.5.3 (the mixture of 5 and 6)** in methanol-d_4 which indicates that compounds **5** and **6** are not enantiomers.

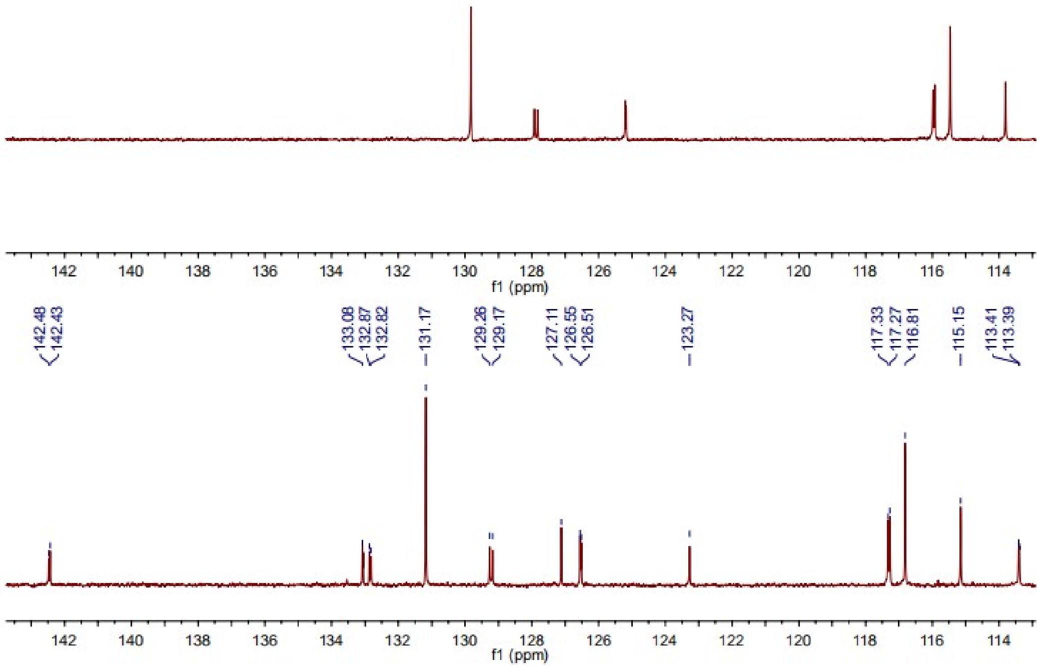

Figure S32. Enlarged ^{13}C NMR and DEPT spectra of **Fr.7.3.6.6.2.5.3 (the mixture of 5 and 6)** in methanol-d_4 which indicates that compounds **5** and **6** are not enantiomers..

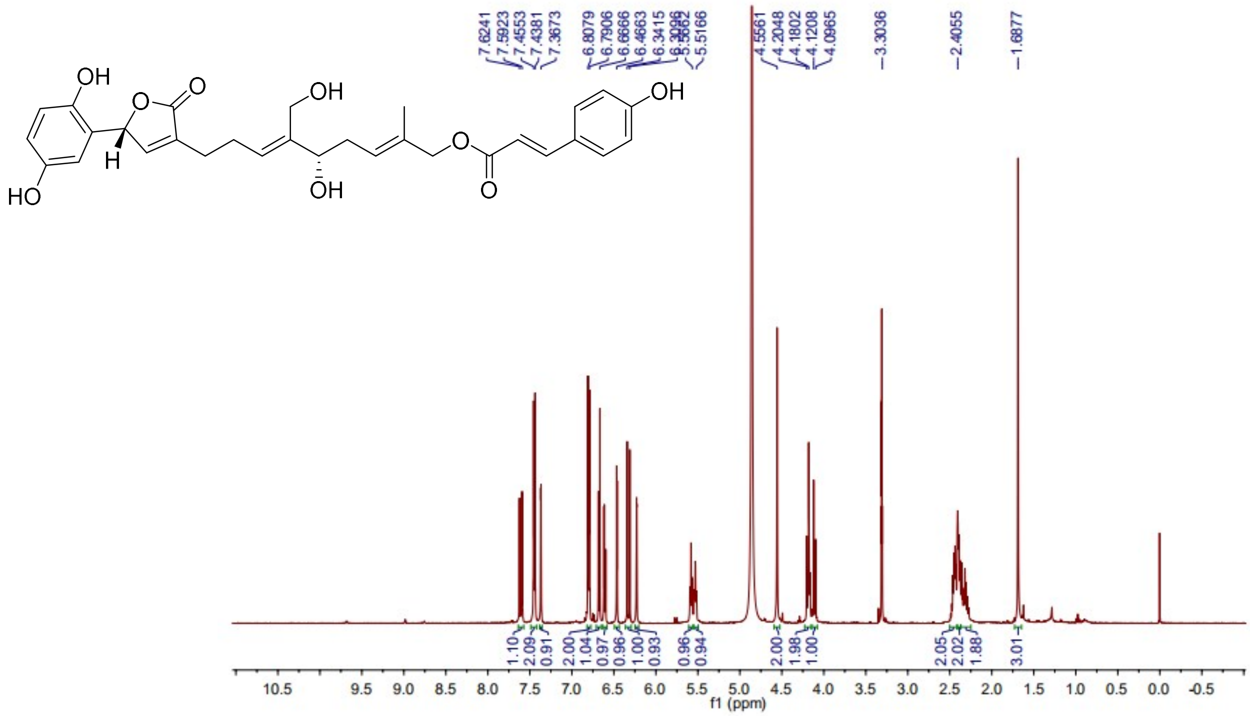

Figure S33.^{1}H NMR spectrum of **5** in methanol-d_4.

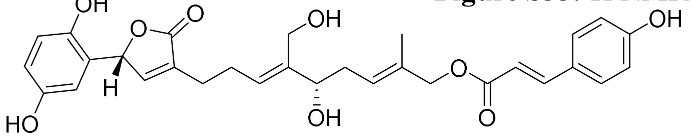

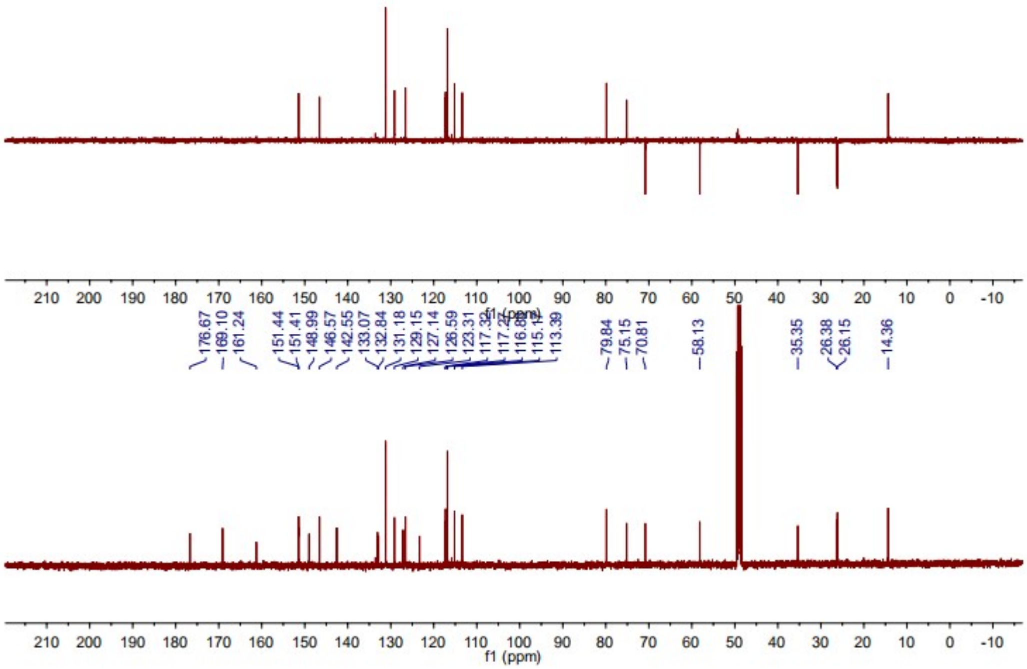

Figure S34. ^{13}C NMR and DEPT spectra of **5** in methanol-d_4.

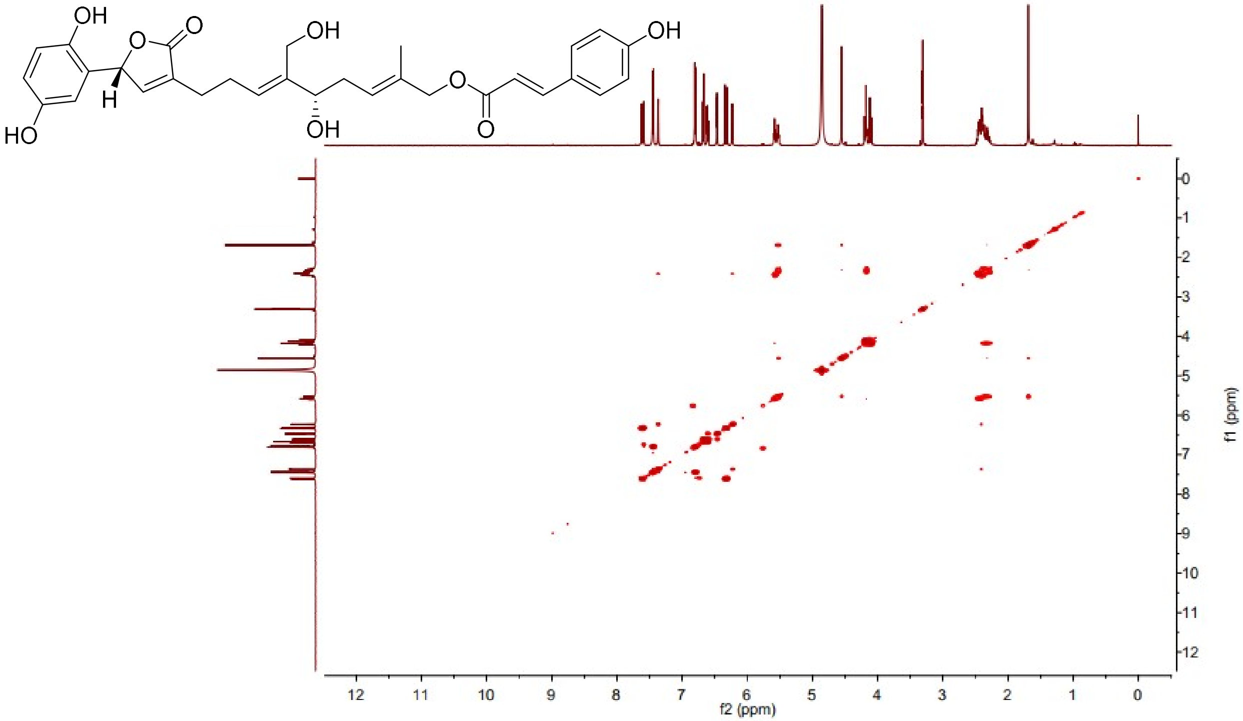

Figure S35. ^{1}H-^{1}H COSY spectrum of **5** in methanol-d_4.

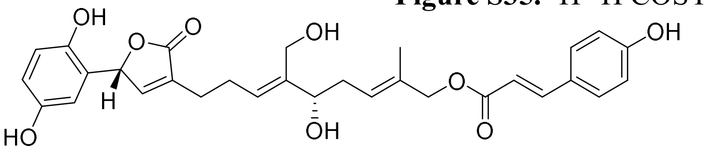

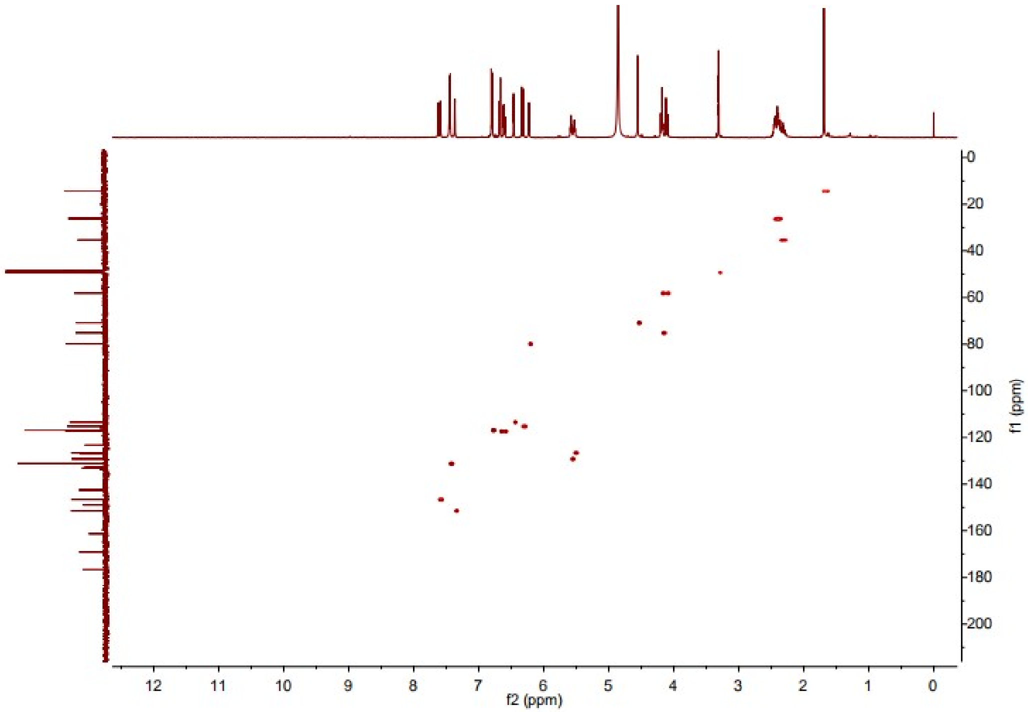

Figure S36. HSQC spectrum of **5** in methanol-*d*4.

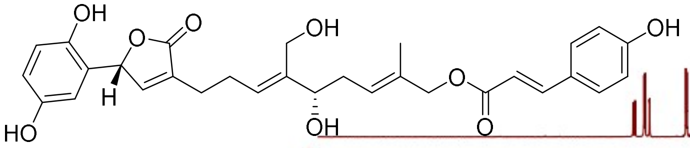

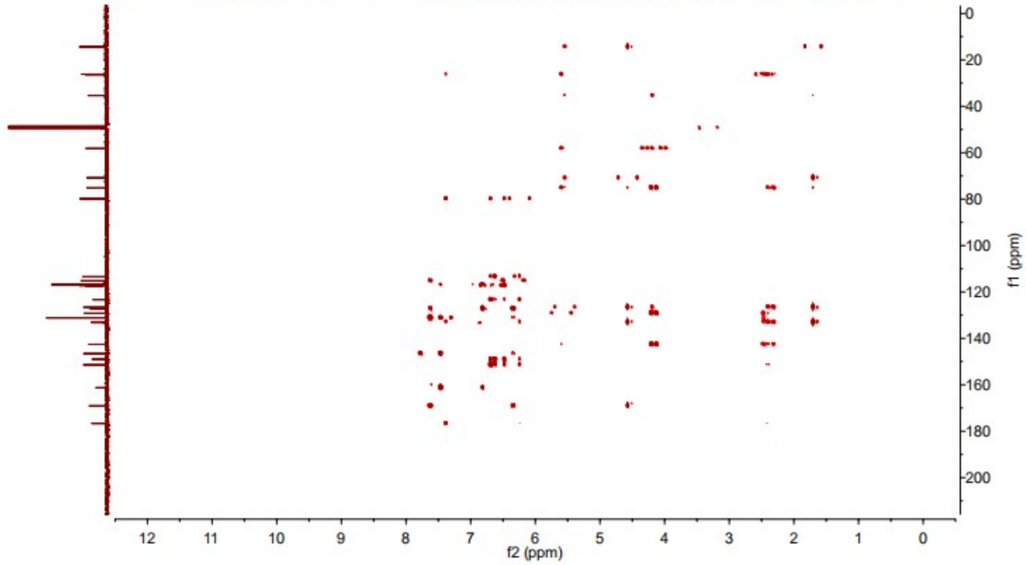

Figure S37. HMBC spectrum of **5** in methanol-*d*4.

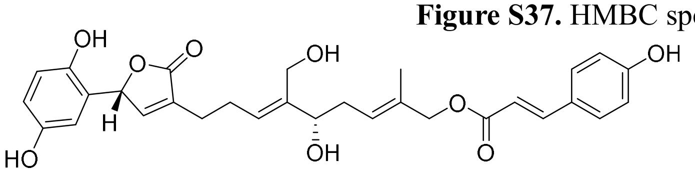

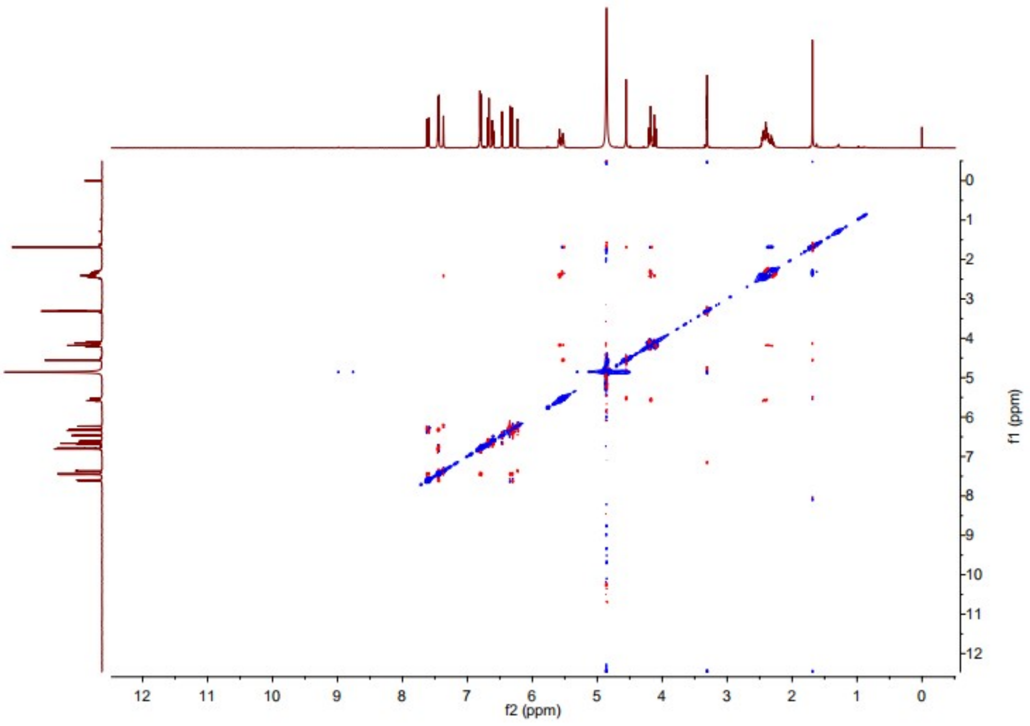

Figure S38. ROESY spectrum of **5** in methanol-d_4.

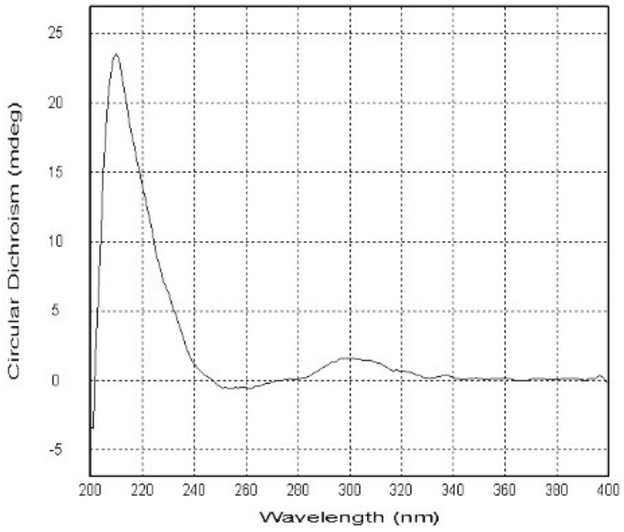

Figure S39. ECD spectrum of **5**.

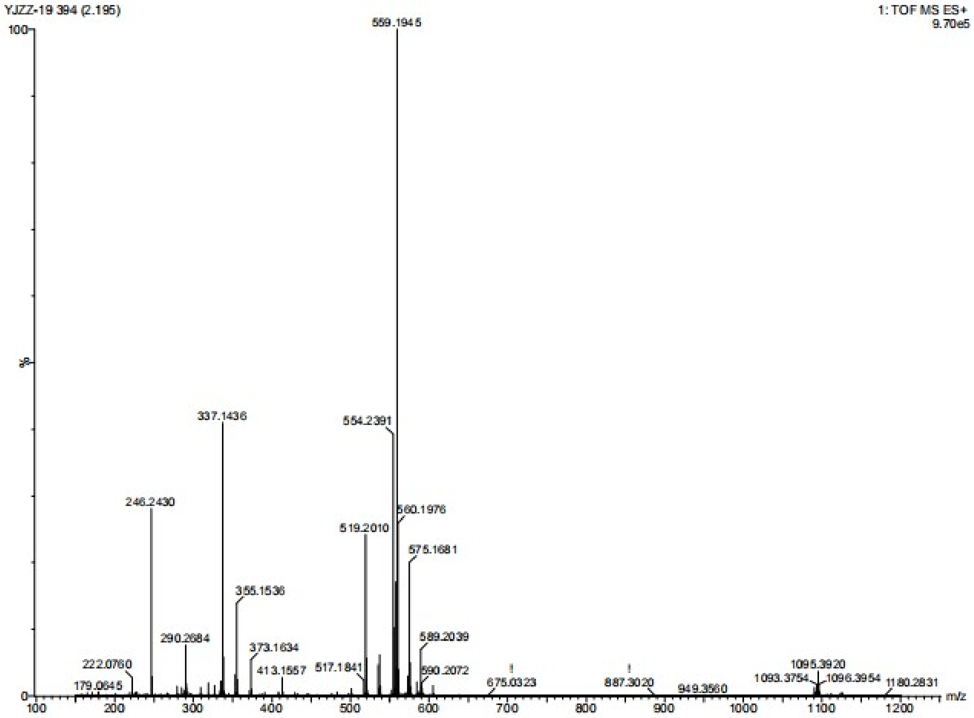

Single Mass Analysis
Tolerance = 10.0 PPM / DBE: min = -1.5, max = 50.0
Element prediction: Off
Number of isotope peaks used for i-FIT = 3

Monoisotopic Mass, Even Electron Ions
84 formula(e) evaluated with 1 results within limits (up to 50 closest results for each mass)
Elements Used:
C: 30-30 H: 0-50 N: 0-4 O: 1-10 Na: 0-1

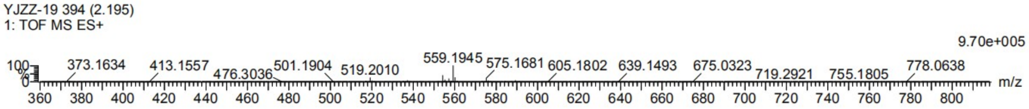

Minimum:				-1.5				
Maximum:		20.0	10.0	50.0				
Mass	Calc. Mass	mDa	PPM	DBE	i-FIT	Norm	Conf(%)	Formula
559.1945	559.1944	0.1	0.2	14.5	604.0	n/a	n/a	C30 H32 O9 Na

Figure S40. HRESIMS of **5**.

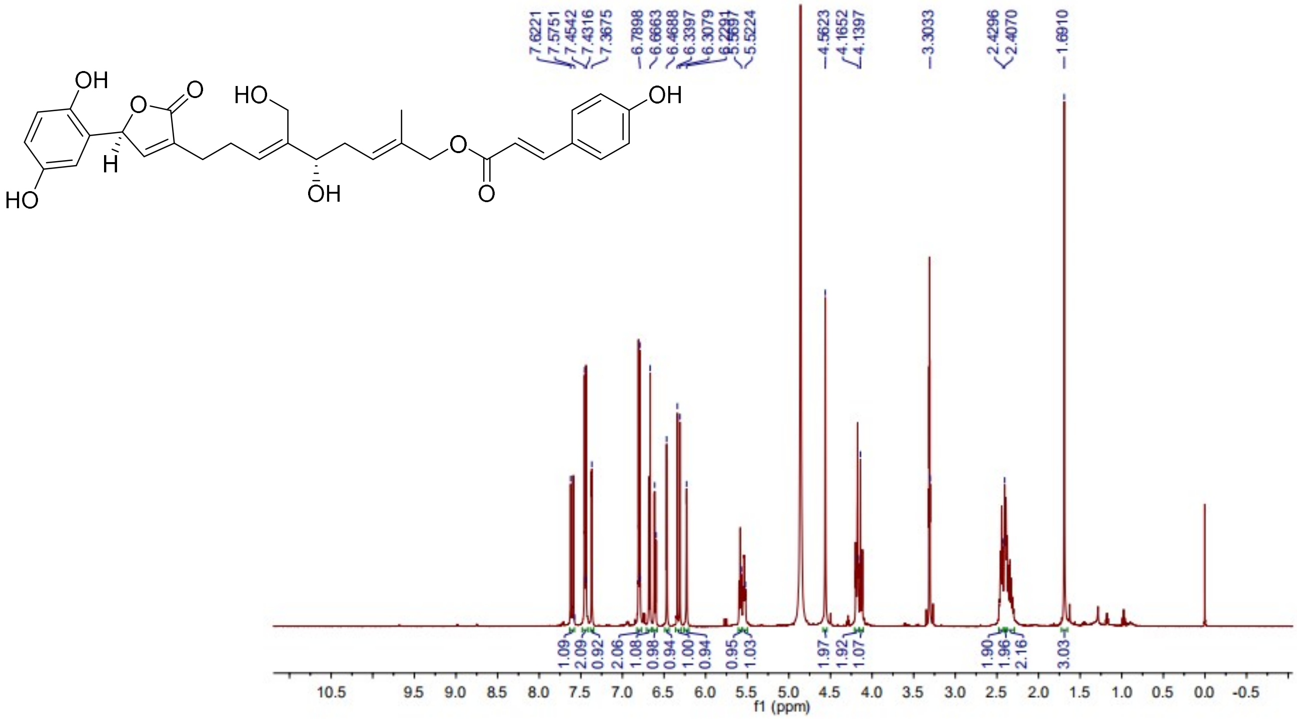

Figure S41. ^{1}H NMR spectrum of **6** in methanol-d_4.

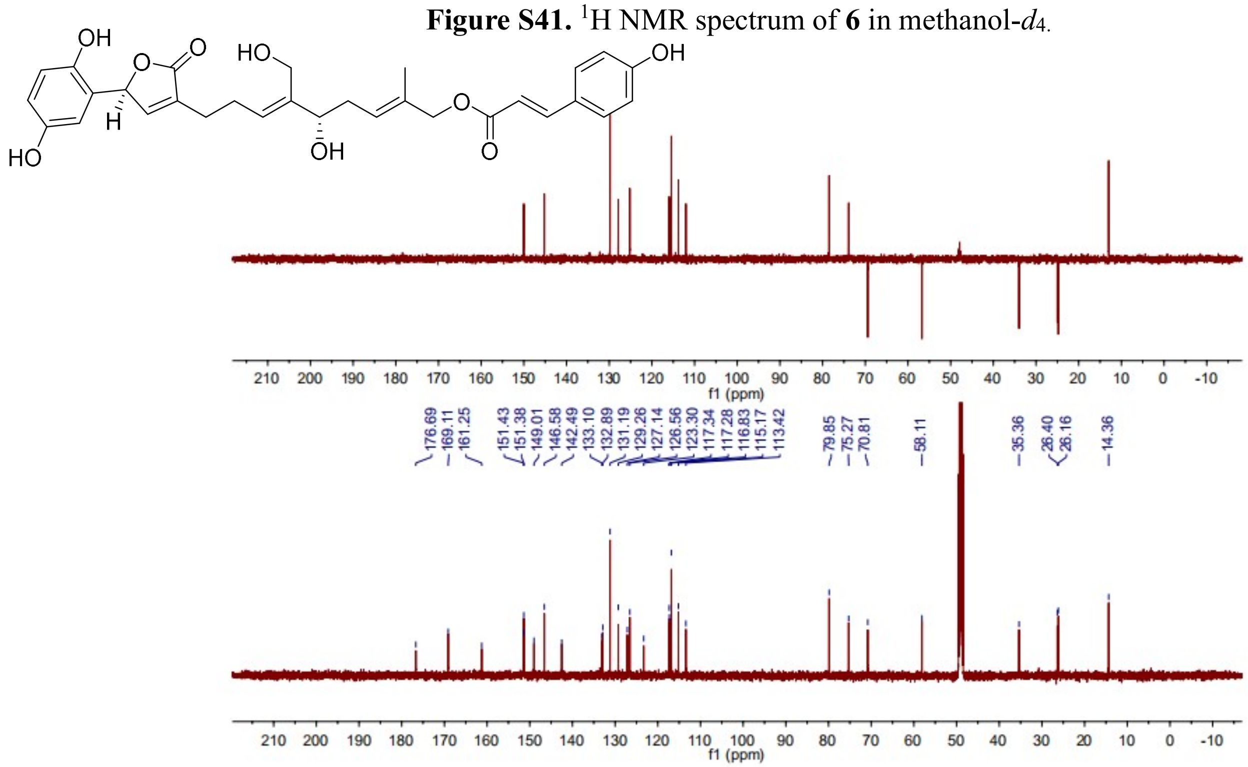

Figure S42. ^{13}C NMR and DEPT spectra of **6** in methanol-d_4.

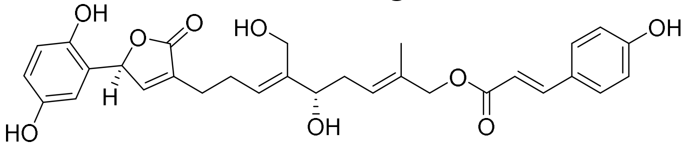

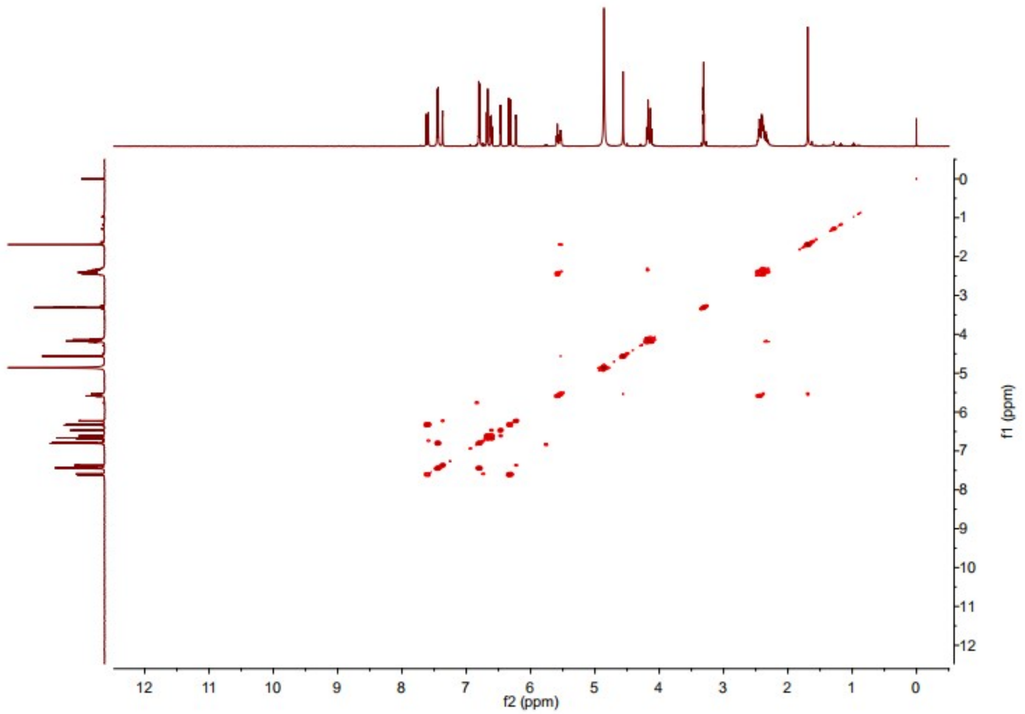

Figure S43. $^{1}H-^{1}H$ COSY spectrum of **6** in methanol-d_4.

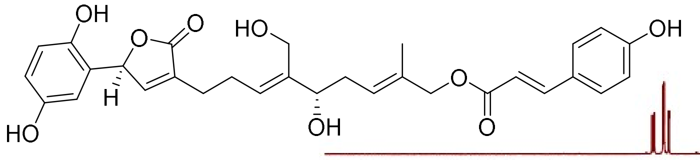

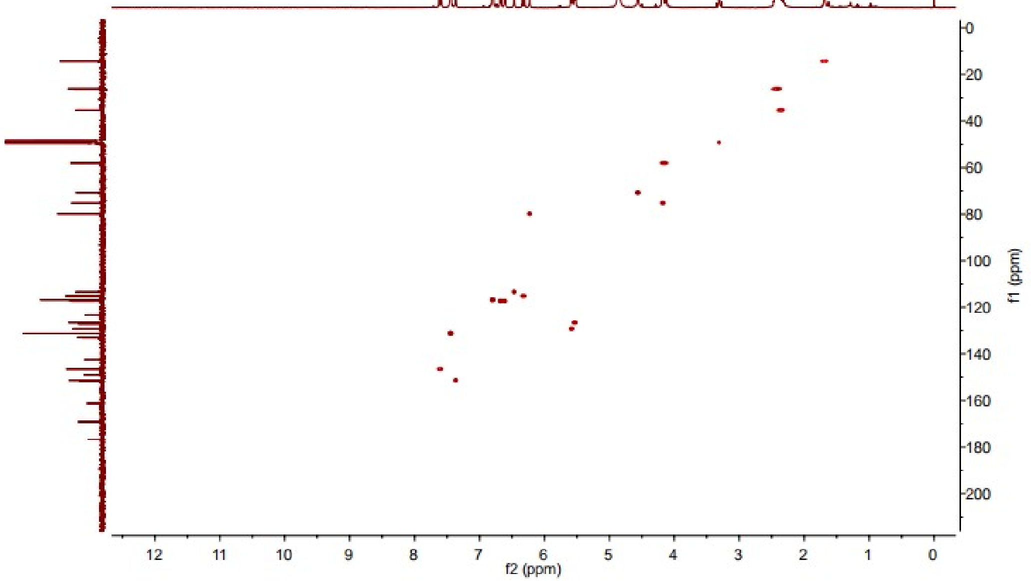

Figure S44. HSQC spectrum of **6** in methanol-d_4.

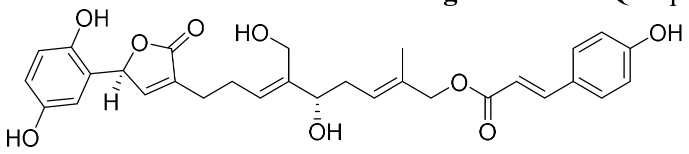

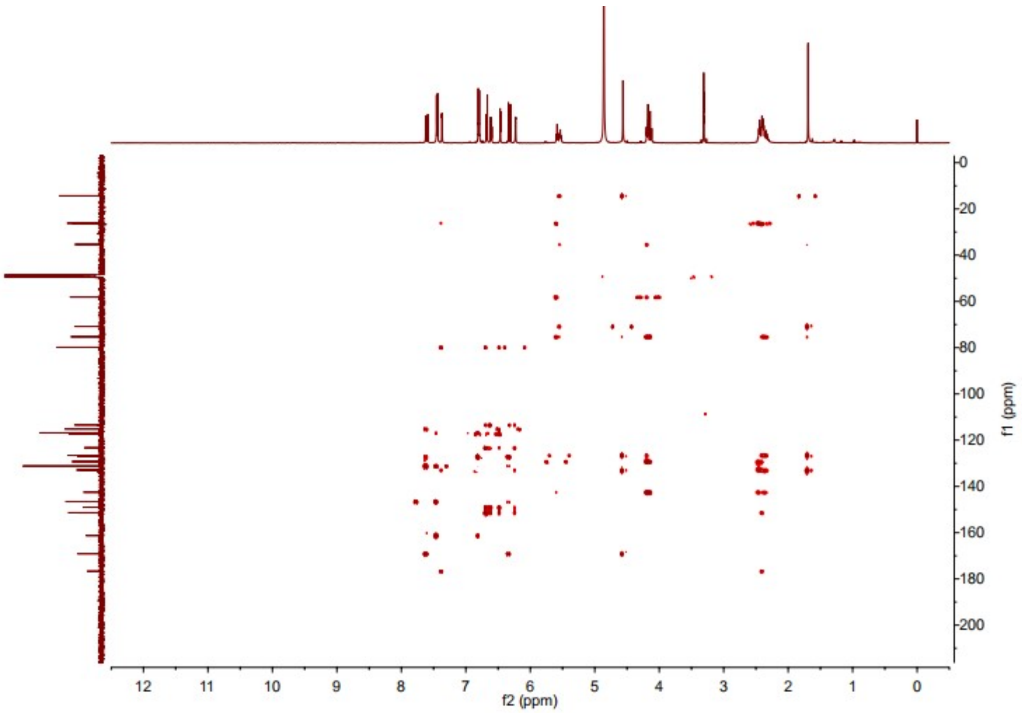

Figure S45. HMBC spectrum of **6** in methanol-d_4.

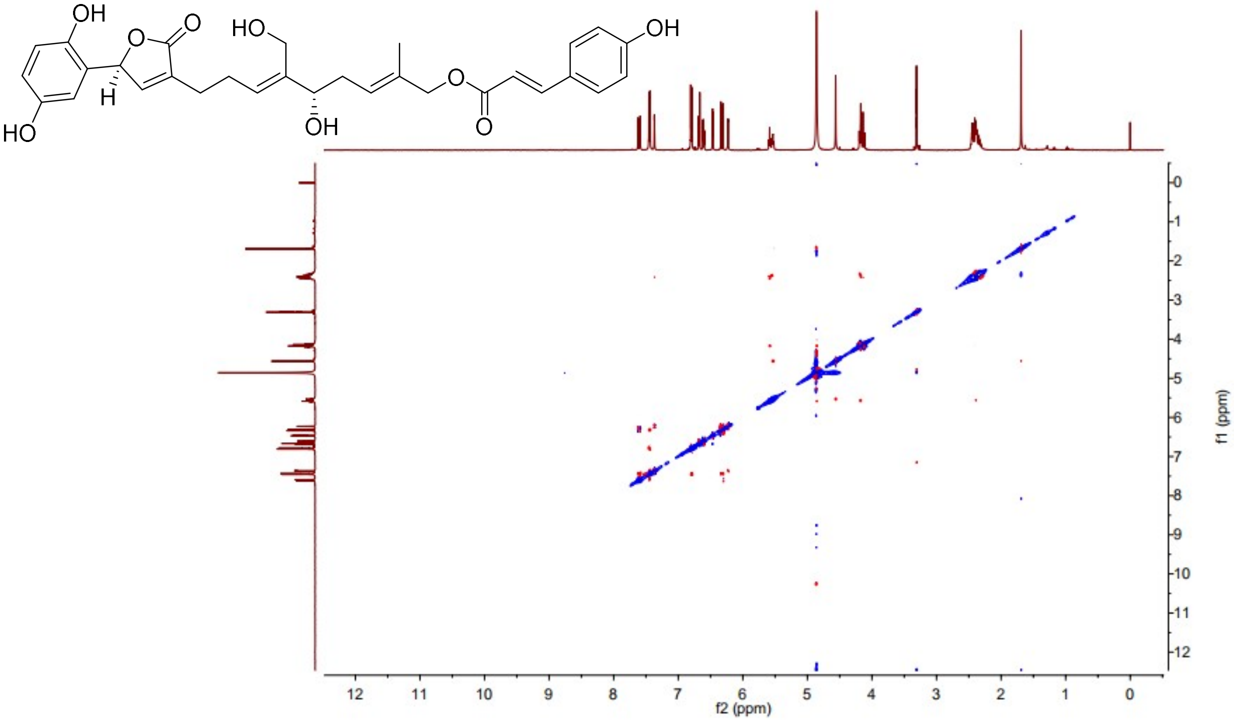

Figure S46. ROESY spectrum of **6** in methanol-d_4.

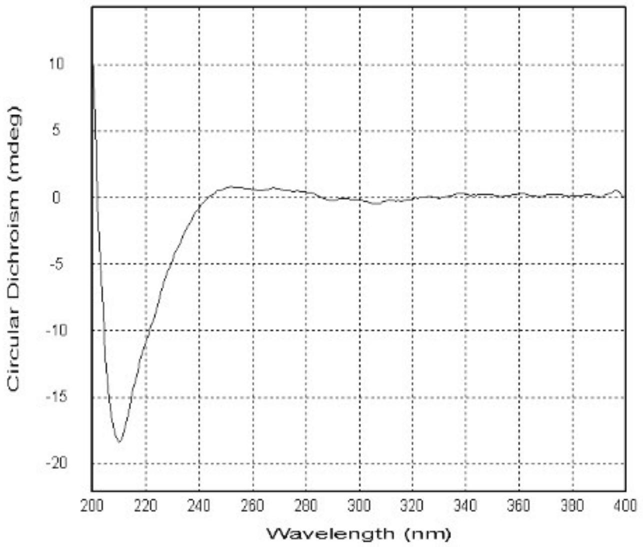

Figure S47. ECD spectrum of **6**.

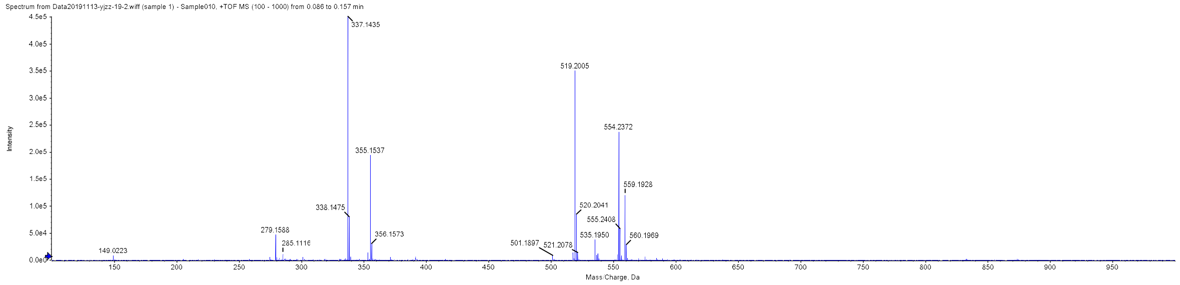

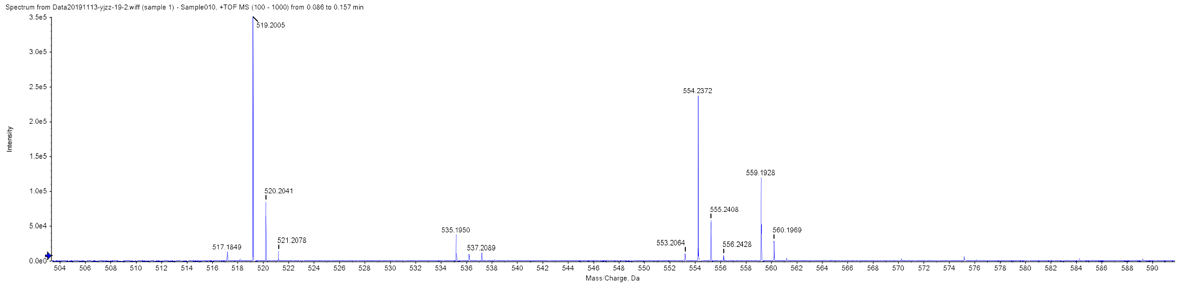

$[M+NH_4]^+$ m/z 554.2371

Hit	Formula	m/z	RDB	ppm
1	$C_{30}H_{32}O_9NH_4$	554.2385	14.0	-2.3

Elements from ~ to $C_{30}H_{32}O_9$

Mass tolerance 5 ppm

Figure S48. HRESIMS of **6**.

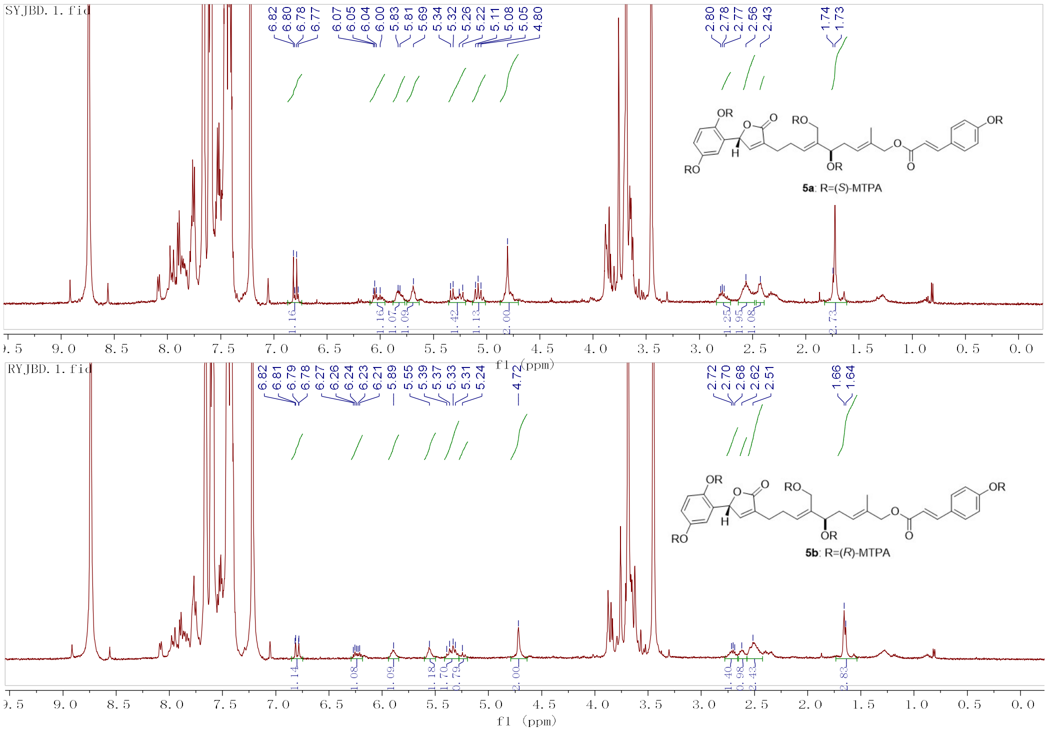

Figure S49. ^{1}H NMR spectrum of **5a** and **5b** in pyridine-d_5.

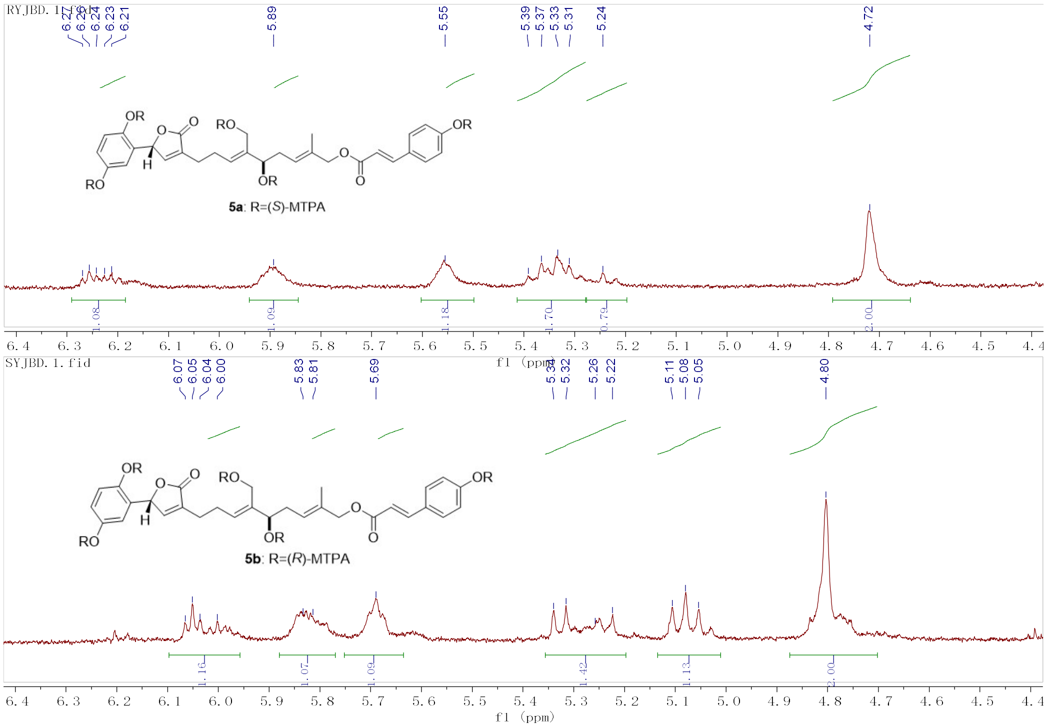

Figure S50. Enlarged ^{1}H NMR spectrum of **5a** and **5b** in pyridine-d_5.

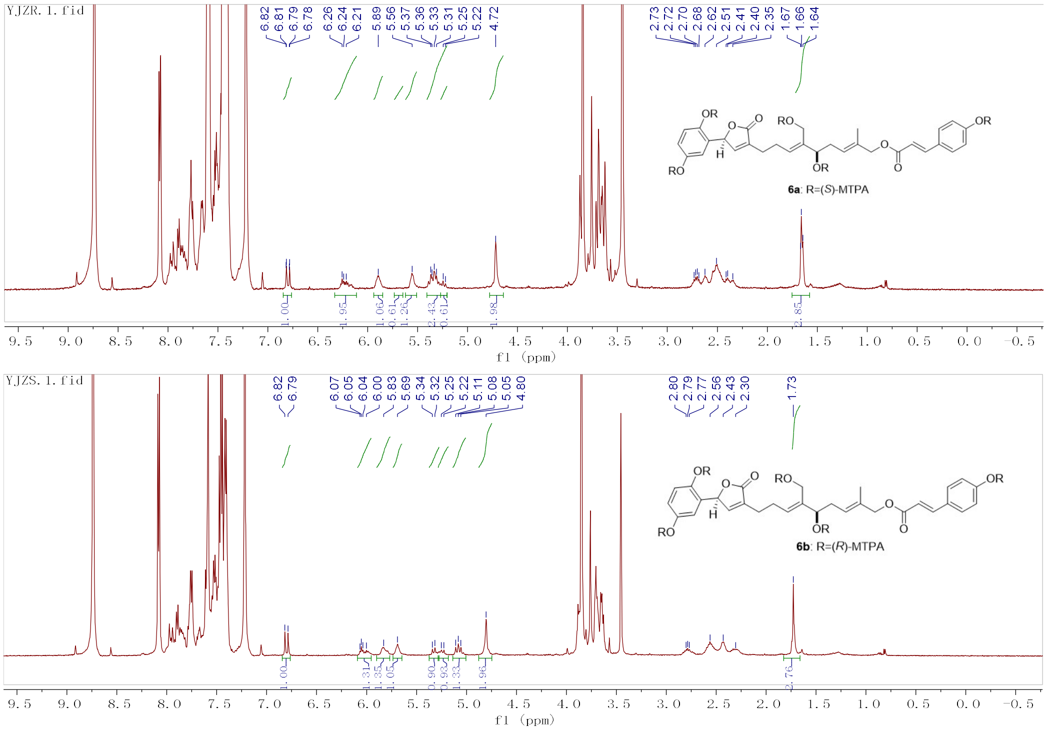

Figure S51. ^{1}H NMR spectrum of **6a** and **6b** in pyridine-d_5.

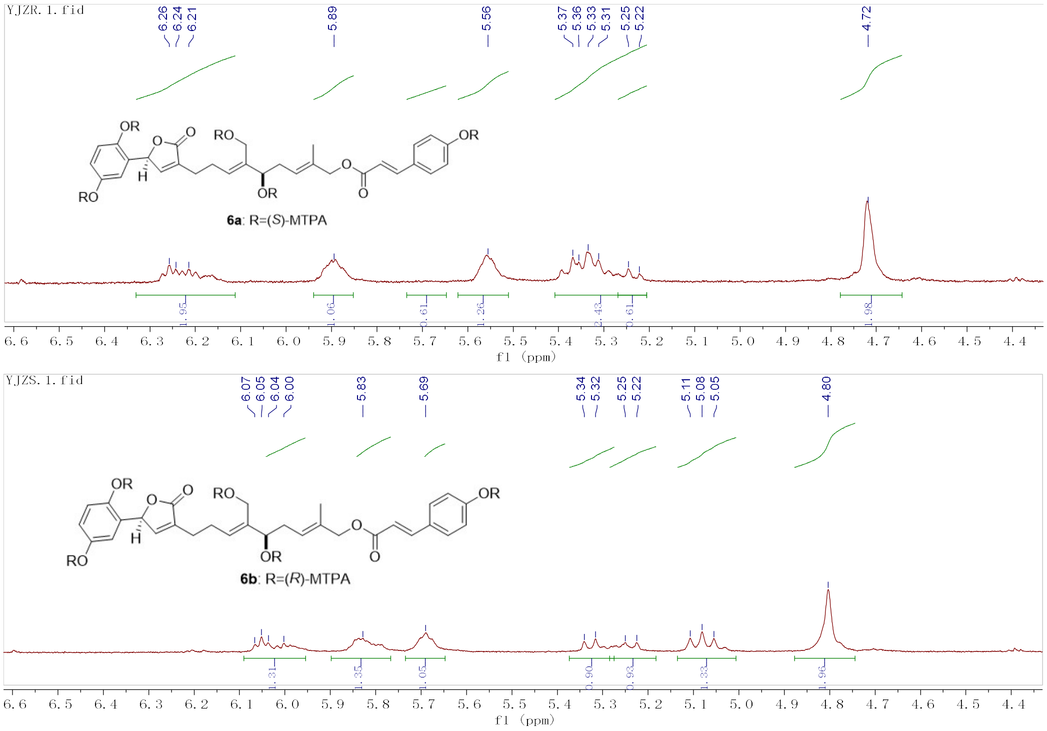

Figure S52. Enlarged ^{1}H NMR spectrum of **6a** and **6b** in pyridine-d_5.

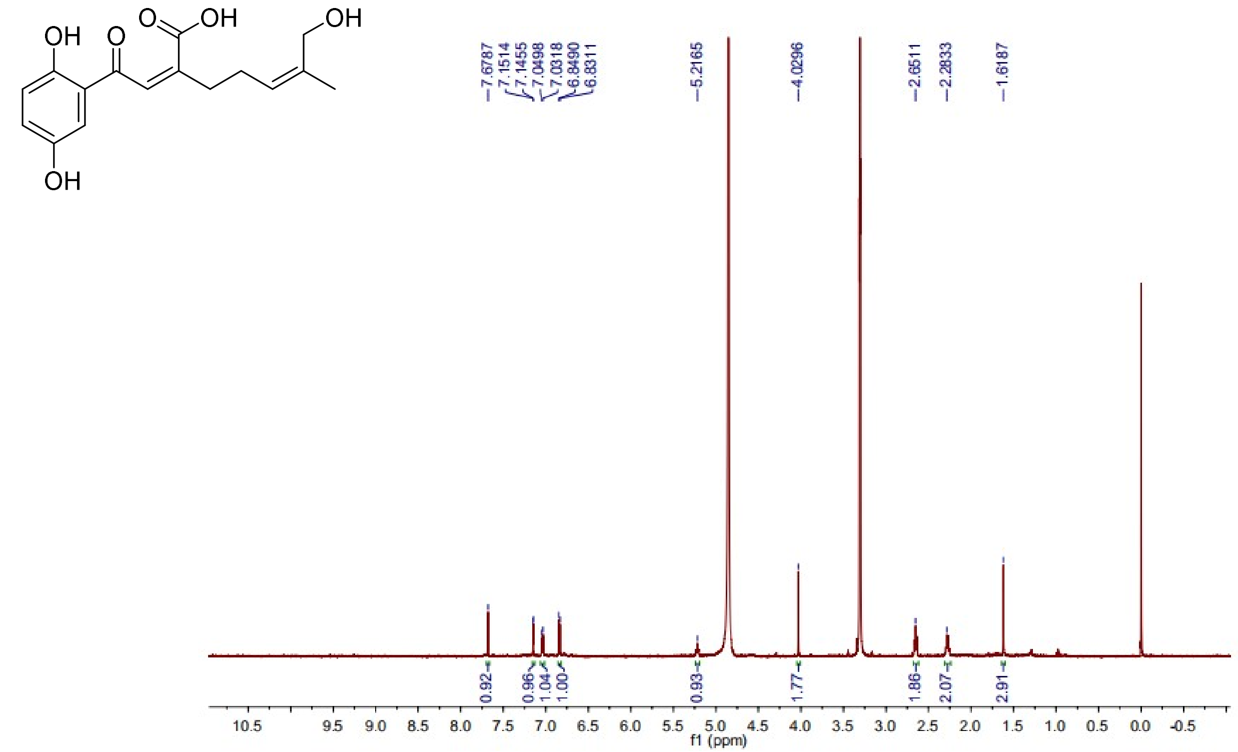

Figure S53. ^{1}H NMR spectrum of **7** in methanol-d_4.

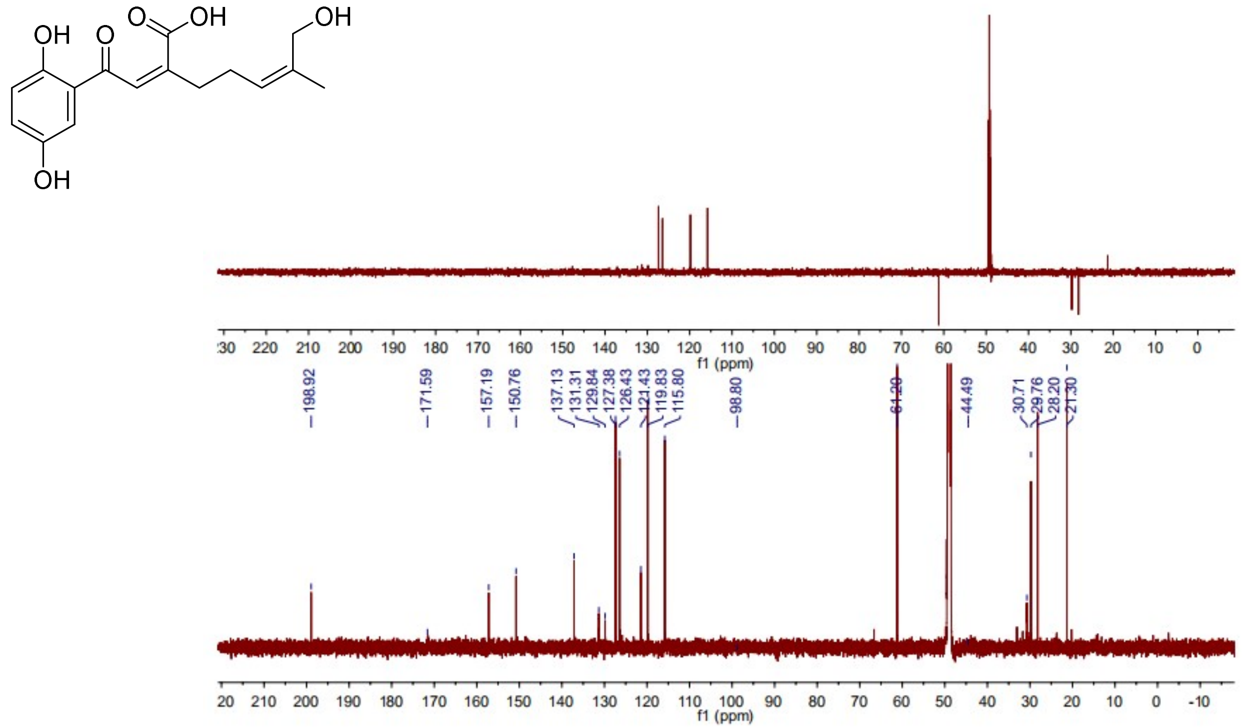

Figure S54. ^{13}C NMR and DEPT spectra of **7** in methanol-d_4.

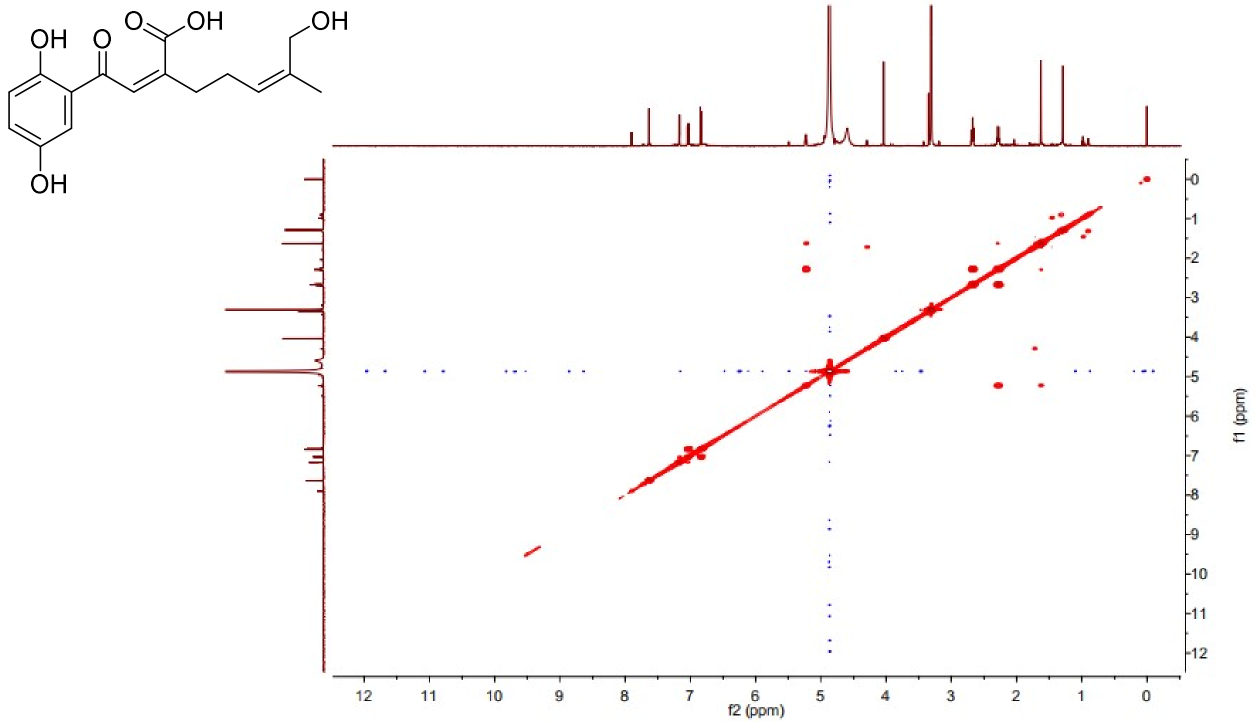

Figure S55. ^{1}H-^{1}H COSY spectrum of **7** in methanol-d_4.

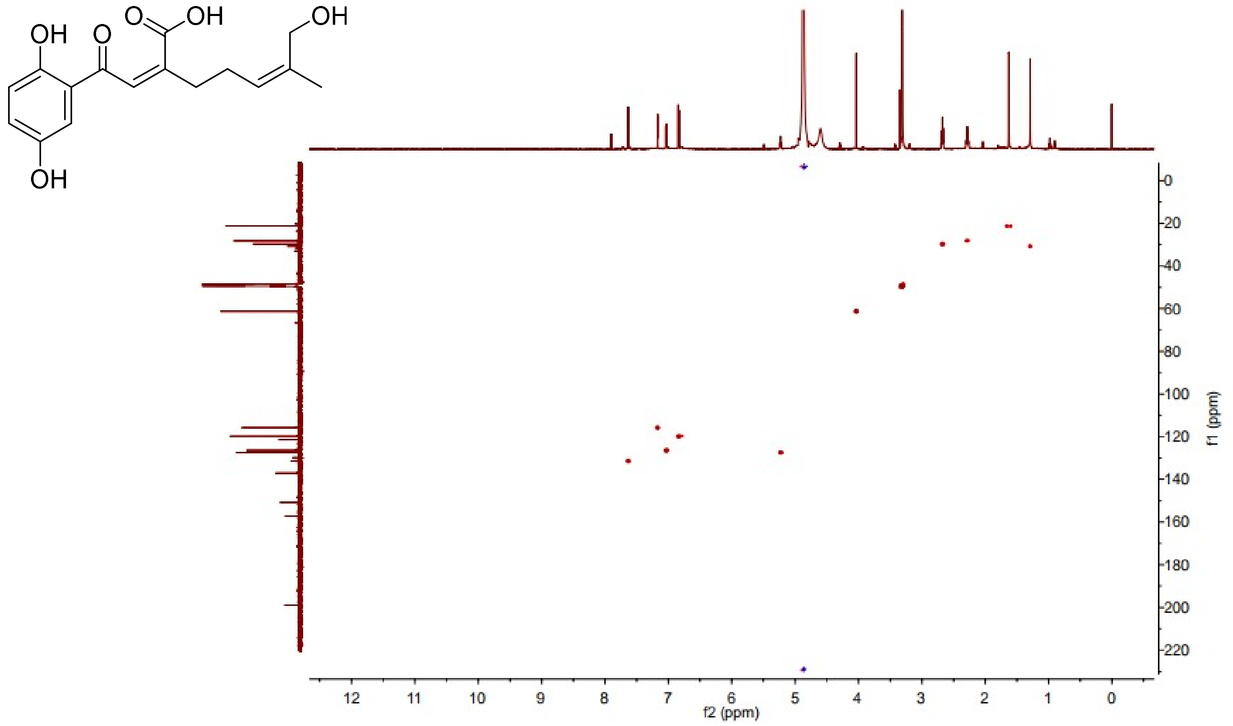

Figure S56. HSQC spectrum of **7** in methanol-d_4.

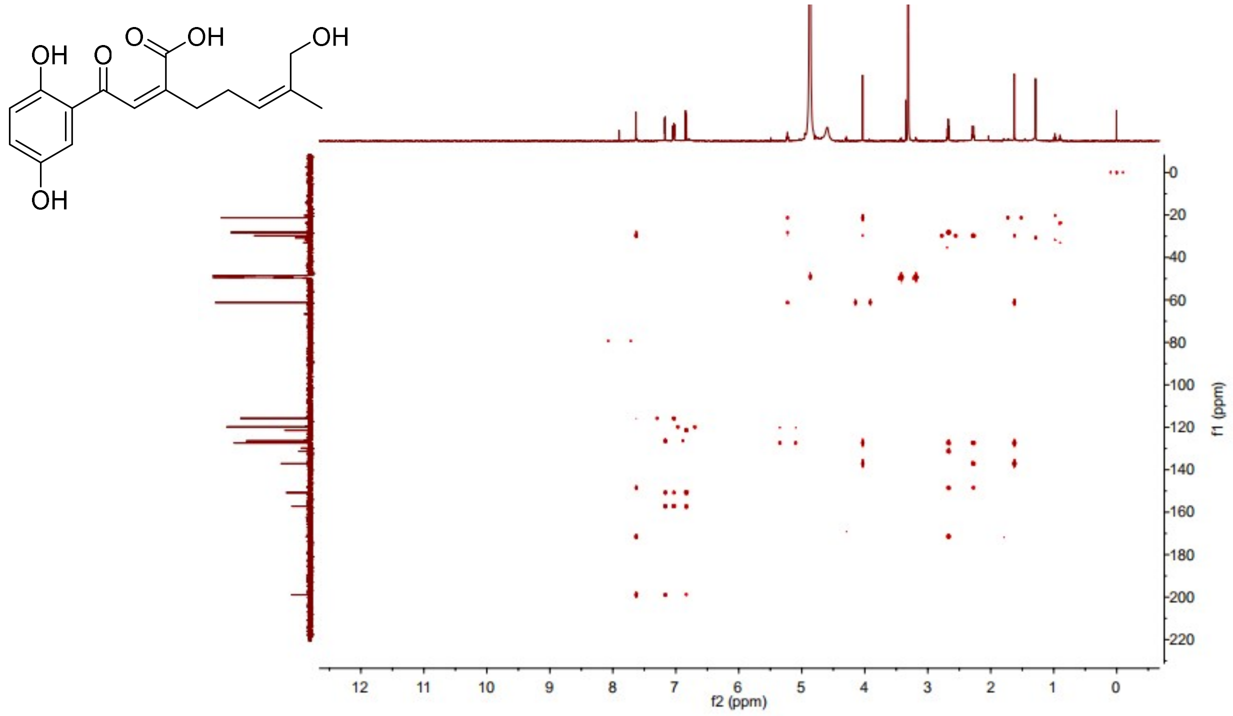

Figure 57. HMBC spectrum of **7** in methanol-d_4.

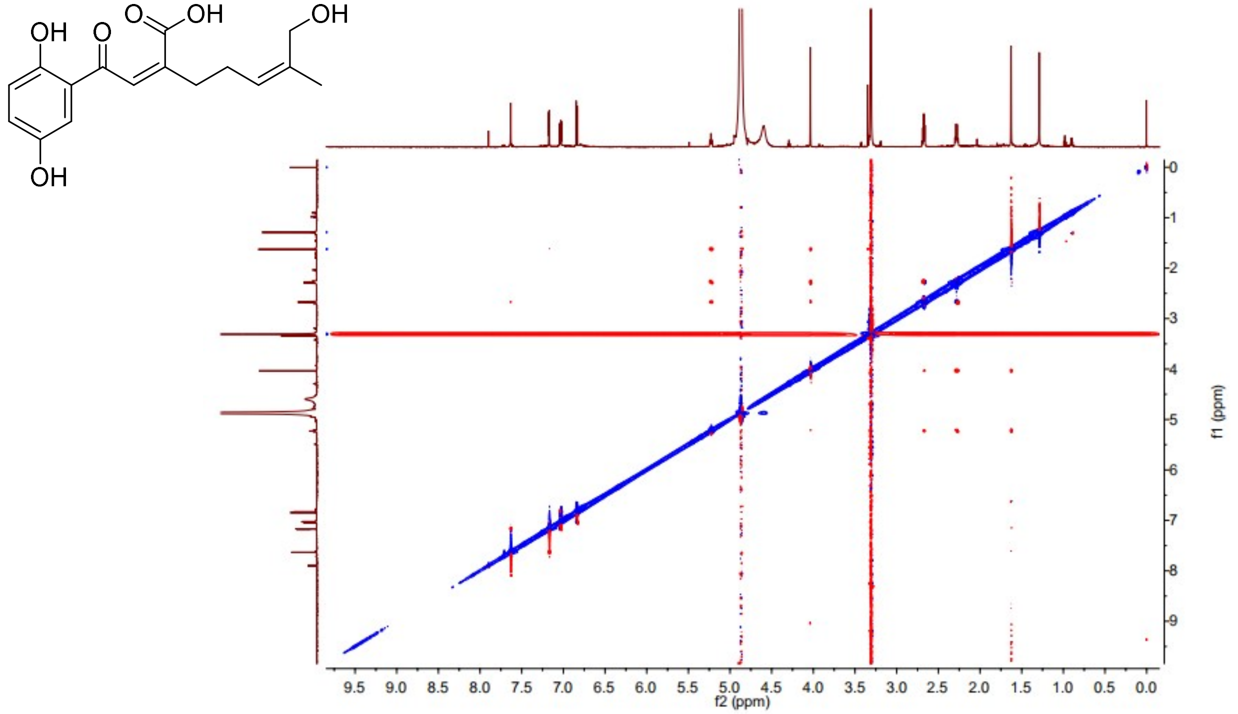

Figure S58. ROESY spectrum of **7** in methanol-d_4.

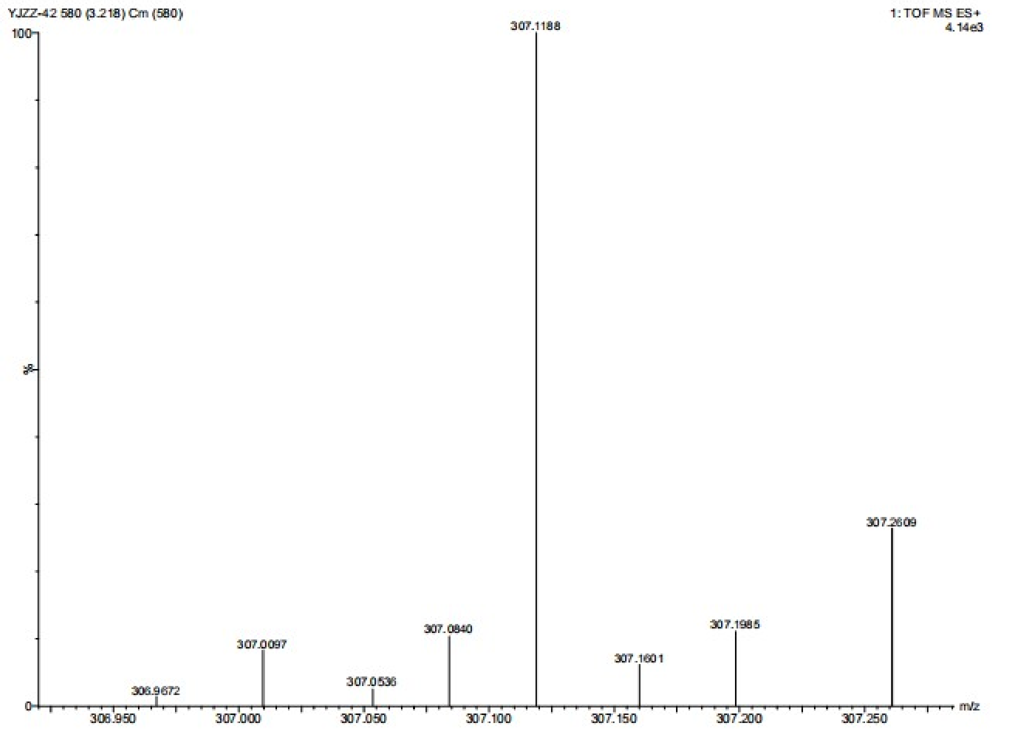

Single Mass Analysis
Tolerance = 10.0 PPM / DBE: min = -1.5, max = 50.0
Element prediction: Off
Number of isotope peaks used for i-FIT = 3

Monoisotopic Mass, Even Electron Ions
97 formula(e) evaluated with 1 results within limits (up to 50 closest results for each mass)
Elements Used:
C: 16-16 H: 0-50 N: 0-4 O: 1-10 Na: 0-1
YJZZ-42 580 (3.218) Cm (580)
1: TOF MS ES+

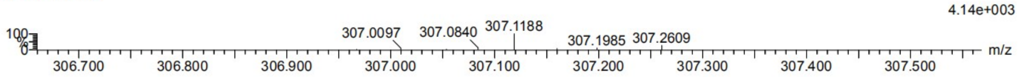

Minimum:				-1.5				
Maximum:		20.0	10.0	50.0				
Mass	Calc. Mass	mDa	PPM	DBE	i-FIT	Norm	Conf(%)	Formula
307.1188	307.1182	0.6	2.0	7.5	64.4	n/a	n/a	C16 H19 O6

Figure S59. HRESIMS of **7**.

ECD calculation for compound **1**

Conformation search using molecular mechanics calculations was performed in CONFLEX version 7.0 with MMFF force field with an energy window for acceptable conformers (ewindow) of 5 kcal/mol above the ground state, a maximum number of conformations per molecule (maxconfs) of 100, and an RMSD cutoff (rmsd) of 0.5Å.

Then the predominant conformers were optimized at B3LYP/6-311+g(2d,p) level in Gaussian 09 [1]. The optimized conformation geometries and thermodynamic parameters of all selected conformations were provided. The optimized conformers of **1** were used for the ECD calculation, which were performed with Gaussian 09 (B3LYP/6-311+g(2d,p)). The solvent effects were taken into account by the polarizable-conductor calculation model (PCM, methanol as the solvent). Percentages for each conformation are shown in Table S1.

Selected conformation of 1 and their percentage

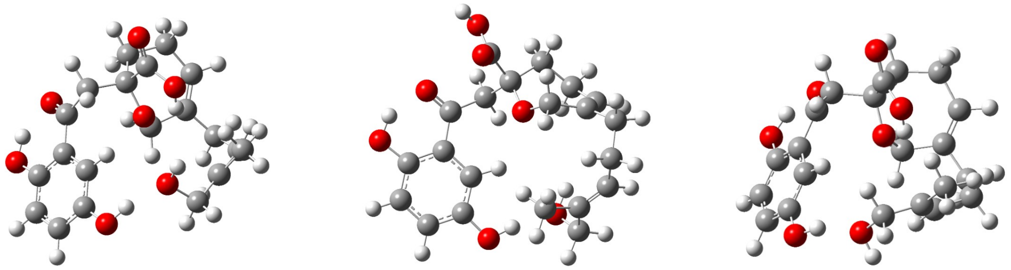

1-1 (59.0%) **1-2** (40.5%) **1-3** (0.5%)

Figure S60. The lowest energy conformers of **1** (the relative populations are in parentheses).

Table S1. Extracted heats and weighting factors of the optimized conformers of **1** at B3LYP/6-31+G(d) level

		B3LYP/6-31+G(d)	
	Conformer	Extracted heats	Boltzmann-calculated contribution (%)
**1**	1	-1342.75854813	59.0%
	2	-1342.75819456	40.5%
	3	-1342.75389876	0.5%

Table S2. The Cartesian coordinates of the lowest energy conformers for **1**

**1-1**	X axis(Å)	Y axis(Å)	Z axis(Å)	**1-2**	X axis(Å)	Y axis(Å)	Z axis(Å)
C	3.8902	2.1004	-0.3912	C	-2.9474	3.3058	-0.1748
C	2.7175	2.0312	0.349	C	-1.7478	2.8322	0.3427
C	2.1052	0.8082	0.6126	C	-1.5249	1.4658	0.5153
C	2.6561	-0.3754	0.1031	C	-2.5285	0.5491	0.1753
C	3.8281	-0.2962	-0.6679	C	-3.7338	1.0366	-0.3642
C	4.4482	0.9308	-0.9034	C	-3.9441	2.404	-0.5347
C	2.0084	-1.7044	0.3314	C	-2.3629	-0.9224	0.3816
C	0.9182	-1.8673	1.3664	C	-1.2496	-1.4783	1.2528
C	-0.4945	-1.7846	0.7643	C	-0.1272	-2.1429	0.4302
O	4.4303	-1.3893	-1.2333	O	-4.7698	0.225	-0.748
O	2.3786	-2.6651	-0.3475	O	-3.1897	-1.6816	-0.133

C	-0.918	-3.0892	0.0588	C	0.8454	-2.8776	1.3834
C	-2.3584	-3.1092	-0.4688	C	1.8604	-1.9694	2.0814
C	-2.7952	-2.0096	-1.3948	C	2.9786	-1.4734	1.2078
C	-2.0815	-0.9491	-1.8196	C	2.9499	-1.2899	-0.1287
C	-2.7046	0.0854	-2.7373	C	4.2257	-0.924	-0.8753
C	-3.3216	1.2897	-1.9997	C	4.248	0.4792	-1.5075
C	-2.3107	2.1838	-1.3271	C	3.9296	1.56	-0.5103
C	-2.3623	2.7263	-0.0921	C	2.8631	2.3848	-0.5089
C	-1.2395	3.6093	0.3961	C	2.6226	3.3119	0.6503
C	-1.4395	-1.5459	1.9597	C	-0.7558	-3.1827	-0.5289
O	-1.5851	-2.3104	2.9018	O	-0.7278	-3.1625	-1.7498
O	-2.0985	-0.3608	1.8722	O	-1.3745	-4.1956	0.1154
C	-0.653	-0.7178	-1.4266	C	1.7114	-1.4468	-0.9797
O	-0.5955	-0.5728	-0.0127	O	0.5416	-1.0754	-0.2489
C	-3.517	2.5834	0.8641	C	1.8112	2.4412	-1.5845
O	-0.4784	2.913	1.382	O	1.5553	2.7628	1.4234
O	2.1744	3.198	0.8012	O	-0.8023	3.7585	0.6722
H	4.3608	3.0612	-0.5801	H	-3.1021	4.3735	-0.3027
H	1.192	0.8058	1.1958	H	-0.5649	1.1365	0.8939
H	5.3595	0.9743	-1.494	H	-4.883	2.7625	-0.9475
H	1.0748	-2.8324	1.8664	H	-0.8563	-0.6995	1.9116
H	1.0627	-1.1249	2.16	H	-1.7284	-2.2065	1.9217
H	3.8564	-2.1715	-1.0721	H	-4.4639	-0.7069	-0.673
H	-0.24	-3.3185	-0.7713	H	0.2732	-3.422	2.1466
H	-0.8139	-3.9292	0.7593	H	1.3927	-3.6549	0.8314
H	-3.0505	-3.1204	0.3824	H	2.3217	-2.5603	2.8835
H	-2.5114	-4.0624	-0.9907	H	1.3721	-1.1192	2.5695
H	-3.8294	-2.1046	-1.7285	H	3.9147	-1.3008	1.7385
H	-1.9637	0.4235	-3.4731	H	5.0909	-1.0126	-0.2041
H	-3.5	-0.3885	-3.3284	H	4.3921	-1.6694	-1.6639
H	-3.8556	1.8988	-2.7396	H	5.2519	0.6677	-1.9077
H	-4.073	0.9198	-1.2963	H	3.5785	0.4972	-2.3708
H	-1.4422	2.4133	-1.9466	H	4.6296	1.6126	0.324
H	-0.5555	3.8857	-0.413	H	3.4981	3.4146	1.2999
H	-1.6228	4.5389	0.8299	H	2.3391	4.3131	0.3115
H	-1.8272	0.0624	1.0196	H	-1.8074	-4.6978	-0.6082
H	0.0239	-1.4877	-1.8089	H	1.7525	-0.7573	-1.8274
H	-0.2868	0.2234	-1.8487	H	1.6585	-2.4583	-1.3945
H	-4.3441	1.9926	0.4661	H	2.1194	1.9639	-2.5168
H	-3.1928	2.1088	1.7944	H	0.8934	1.9553	-1.2435
H	-3.9202	3.5718	1.11	H	1.579	3.4824	-1.8336
H	-1.0802	2.7669	2.1359	H	1.8537	1.8602	1.6501
H	1.2619	3.0253	1.1232	H	0.0048	3.3118	1.0119

**1-3**	X axis(Å)	Y axis(Å)	Z axis(Å)
C	-3.968	2.2265	0.3171
C	-2.7199	2.1669	-0.2899
C	-2.1018	0.9451	-0.5503
C	-2.7287	-0.2488	-0.1647
C	-3.9768	-0.179	0.4796
C	-4.5992	1.0479	0.706
C	-2.096	-1.5858	-0.3887
C	-0.9575	-1.7568	-1.3684
C	0.4208	-1.7863	-0.6895
O	-4.657	-1.2828	0.923
O	-2.5311	-2.5524	0.242
C	0.6743	-3.0619	0.1434
C	2.0752	-3.1693	0.7661
C	2.5752	-2.0333	1.6167
C	1.947	-0.8773	1.9053
C	2.6193	0.1945	2.743
C	3.3791	1.2597	1.9244
C	2.5068	2.0594	0.9866
C	2.7696	2.421	-0.2881
C	1.7791	3.1451	-1.1779
C	1.4477	-1.7657	-1.8406
O	1.5189	-2.5997	-2.7311
O	2.2863	-0.701	-1.7703
C	0.5651	-0.5665	1.4155
O	0.6038	-0.5301	-0.0058
C	4.0782	2.1512	-0.9905
O	0.5471	3.4096	-0.5138
O	-2.1168	3.3465	-0.6128
H	-4.4404	3.1874	0.5007
H	-1.1325	0.9508	-1.0356
H	-5.5693	1.0833	1.1946
H	-1.141	-2.6878	-1.9214
H	-1.0106	-0.9663	-2.1262
H	-4.0745	-2.0669	0.8085
H	-0.065	-3.1586	0.9462
H	0.5271	-3.9445	-0.4939
H	2.8079	-3.3288	-0.0348
H	2.1009	-4.0789	1.3796
H	3.5812	-2.1881	2.0089
H	1.8754	0.6758	3.391
H	3.3357	-0.2765	3.4296
H	3.8441	1.9602	2.6287

H	4.1916	0.7586	1.3901
H	1.5485	2.3433	1.4189
H	2.1922	4.0983	-1.5242
H	1.5513	2.529	-2.0538
H	2.0392	-0.1796	-0.9678
H	-0.1932	-1.248	1.8123
H	0.2528	0.4288	1.746
H	4.8363	1.6973	-0.3496
H	3.9218	1.4879	-1.8465
H	4.5016	3.0914	-1.3611
H	0.7598	4.0915	0.1524
H	-1.1536	3.1986	-0.7444

References

(1) M. J. Frisch, G. W. Trucks, H. B. Schlegel, G. E. Scuseria, M. A. Robb, J. R. Cheeseman, G. Scalmani, V. Barone, B. Mennucci, G. A. Petersson, H. Nakatsuji, M. Caricato, X. Li, H. P. Hratchian, A. F. Izmaylov, J. Bloino, G. Zheng, J. L. Sonnenberg, M. Hada, M. Ehara, K. Toyota, R. Fukuda, J. Hasegawa, M. Ishida, T. Nakajima, Y. Honda, O. Kitao, H. Nakai, T. Vreven, J. A. Montgomery, J. E. Peralta, F. Ogliaro, M. Bearpark, J. J. Heyd, E. Brothers, K. N. Kudin, V. N. Staroverov, R. Kobayashi, J. Normand, K. Raghavachari, A. Rendell, J. C. Burant, S. S. Iyengar, J. Tomasi, M. Cossi, N. Rega, J. M. Millam, M. Klene, J. E. Knox, J. B. Cross, V. Bakken, C. Adamo, J. Jaramillo, R. Gomperts, R. E. Stratmann, O. Yazyev, A. J. Austin, R. Cammi, C. Pomelli, J. W. Ochterski, R. L. Martin, K. Morokuma, V. G. Zakrzewski, G. A. Voth, P. Salvador, J. J. Dannenberg, S. Dapprich, A. D. Daniels, O. Farkas, J. B. Foresman, J. V. Ortiz, J. Cioslowski, D. J. Fox, Gaussian, Inc., Wallingford CT, 2009.

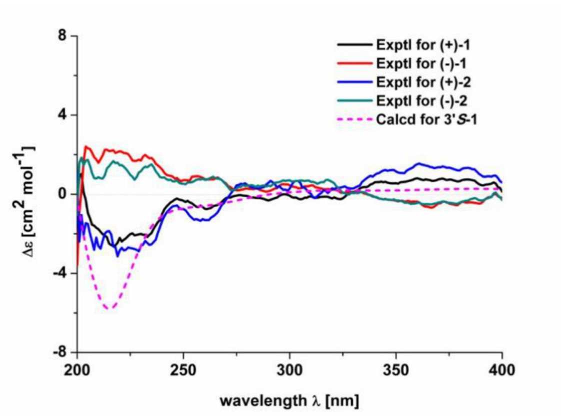

Figure S61. The calculated and experimental ECD spectra of **1** and **2**.

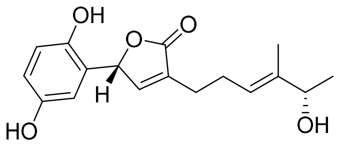

5c

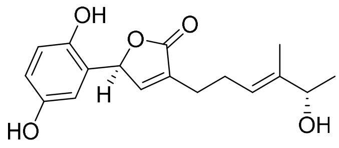

6c

Figure S62. The calculated model compounds **5c** and **6c.**

ECD calculation for compounds **5** and **6**

MMFF conformational search generated low-energy conformers within a 10 kcal/mol energy window were performed with Discovery Studio Client 4.0,[1] which given the 4 stable conformer of (7'*R*, 10'*S*)-**5c** as **5c1, 5c2, 5c3** and **5c4** and 4 stable conformers of (6'*S*,7'*R*)-**6c** as **6c1, 6c2, 6c3** and **6c4.** The ECD calculations was using TD-DFT-B3LYP/6-31G (d,p) of theory on B3LYP/6-31G(d,p) optimized geometries through the CPCM model (in MeOH). The calculated ECD curve of **5c** and **6c** was generated using SpecDis 1.61[2] with σ=0.20 ev, UV shift 1 nm, σ=0.20 ev, UV shift 1 nm, respectively. All the above calculations were carried out with the Gaussian 09 package of programs.[3]

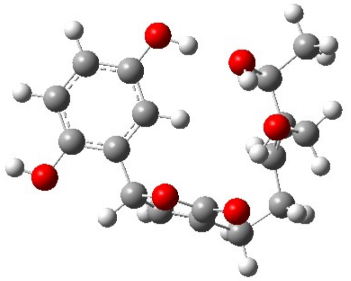

5c1(98.39%)

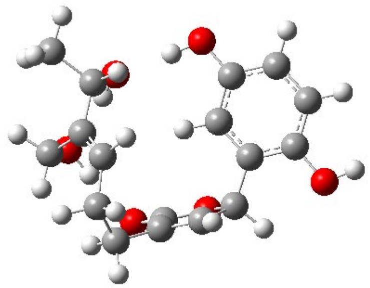

5c2(0.3%)

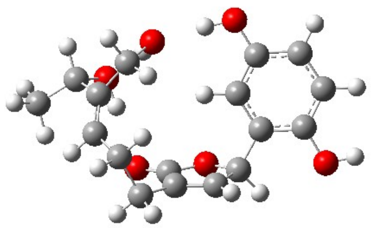

5c3 (0.97%)

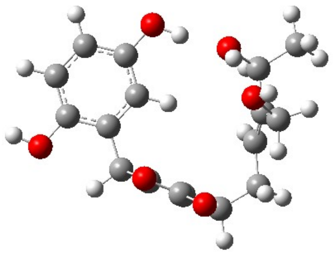

5c4 (0.35%)

Table S3. Standard orientation of **5c** in MeOH

**5c1**

Center Number	Atomic Number	Atomic Type	X	Y	Z
1	8	0	1.463270	2.376373	0.452175
2	8	0	2.523892	0.397950	2.024134
3	8	0	-0.886608	-1.733195	1.362310
4	8	0	1.299971	-2.177481	1.637155
5	6	0	3.416499	0.204019	0.903210
6	6	0	0.327956	-2.123806	0.897723
7	8	0	-1.108327	3.200498	0.034201
8	8	0	-4.568049	-1.132547	-0.169072
9	6	0	3.435699	2.870128	-0.876584
10	6	0	2.274341	1.897687	-0.654264
11	6	0	2.705544	0.458564	-0.410302
12	6	0	2.402958	-0.497374	-1.303817
13	6	0	2.678572	-1.973933	-1.211731
14	6	0	1.397167	-2.828427	-1.405144
15	6	0	0.233952	-2.401330	-0.555006
16	6	0	-1.033990	-2.152892	-0.913573
17	6	0	-1.837293	-1.679874	0.264495
18	6	0	-3.735960	-0.041187	-0.111043
19	6	0	-2.370382	-0.267978	0.104667
20	6	0	-1.488830	0.815461	0.147463
21	6	0	-1.943620	2.127979	-0.007230
22	6	0	-3.313446	2.347147	-0.214454
23	6	0	-4.196869	1.271771	-0.266919
24	1	0	1.663836	1.813883	1.230266
25	1	0	1.975099	-0.407279	2.077704
26	1	0	4.226419	0.925588	1.045990
27	1	0	3.859745	-0.793042	0.943252

28	1	0	-0.182242	2.896661	0.196806
29	1	0	-5.483751	-0.833741	-0.253324
30	1	0	4.104596	2.904067	-0.011568
31	1	0	4.018605	2.578205	-1.755823
32	1	0	3.044434	3.878633	-1.039551
33	1	0	1.627974	1.921604	-1.538760
34	1	0	1.867654	-0.190043	-2.204347
35	1	0	3.387057	-2.269435	-1.996482
36	1	0	3.135129	-2.232431	-0.255853
37	1	0	1.081683	-2.795097	-2.452877
38	1	0	1.648979	-3.873352	-1.185782
39	1	0	-1.460092	-2.226161	-1.907136
40	1	0	-2.657084	-2.363576	0.503525
41	1	0	-0.432484	0.642294	0.316310
42	1	0	-3.675940	3.363011	-0.334479
43	1	0	-5.256592	1.451689	-0.430039

**5c2**					
1	8	0	-1.586353	2.173962	-0.788937
2	8	0	-2.976466	-0.038454	-1.692774
3	8	0	1.109028	-1.809363	-1.453589
4	8	0	-1.052103	-2.163480	-1.972010
5	6	0	-3.513097	-0.136445	-0.354826
6	6	0	-0.168167	-2.119941	-1.128187
7	8	0	0.949866	3.152618	-0.405453
8	8	0	4.541748	-0.982364	0.489517
9	6	0	-3.458277	2.860217	0.605101
10	6	0	-2.242002	1.938041	0.484123
11	6	0	-2.561521	0.459935	0.659549

12	6	0	-1.969037	-0.235479	1.642586
13	6	0	-2.122680	-1.701732	2.001448
14	6	0	-1.582403	-2.720622	0.959867
15	6	0	-0.269146	-2.351265	0.331212
16	6	0	0.947504	-2.119498	0.844992
17	6	0	1.897875	-1.685077	-0.237589
18	6	0	3.677376	0.061223	0.266620
19	6	0	2.357366	-0.244249	-0.087937
20	6	0	1.443745	0.791115	-0.303362
21	6	0	1.822355	2.130093	-0.189051
22	6	0	3.147444	2.429577	0.158916
23	6	0	4.061177	1.403088	0.386003
24	1	0	-1.953574	1.519373	-1.418333
25	1	0	-2.342708	-0.772269	-1.813087
26	1	0	-4.453077	0.423789	-0.375103
27	1	0	-3.762271	-1.173732	-0.114818
28	1	0	0.063015	2.786792	-0.634511
29	1	0	5.430503	-0.635506	0.647774
30	1	0	-4.197203	2.656537	-0.175550
31	1	0	-3.941150	2.738962	1.580096
32	1	0	-3.140187	3.901688	0.503146
33	1	0	-1.509917	2.225830	1.246189
34	1	0	-1.281080	0.317153	2.281543
35	1	0	-1.600869	-1.866878	2.948570
36	1	0	-3.174001	-1.948892	2.190026
37	1	0	-1.498734	-3.698041	1.447813
38	1	0	-2.302728	-2.845998	0.146560
39	1	0	1.249645	-2.168723	1.884353
40	1	0	2.759917	-2.350930	-0.329072

41	1	0	0.419559	0.562015	-0.573607
42	1	0	3.452198	3.467167	0.249939
43	1	0	5.085813	1.644412	0.657255

**5c3**					
Center Number	Atomic Number	Atomic Type	X	Y	Z
1	8	0	-2.564922	1.248193	-1.711573
2	8	0	-1.073628	2.428219	0.225483
3	8	0	0.669645	-1.515283	-1.461200
4	8	0	-1.575400	-1.643555	-1.531139
5	6	0	-1.886161	1.763360	1.216606
6	6	0	-0.541791	-1.775546	-0.897174
7	8	0	1.593753	3.129978	0.456980
8	8	0	4.553703	-1.483619	-0.418895
9	6	0	-4.785579	0.448116	-1.066547
10	6	0	-3.544845	1.217507	-0.630188
11	6	0	-2.814569	0.730540	0.616099
12	6	0	-2.940689	-0.533050	1.045172
13	6	0	-2.149107	-1.284063	2.077301
14	6	0	-1.457958	-2.533569	1.457760
15	6	0	-0.339762	-2.203708	0.511415
16	6	0	0.982239	-2.191794	0.735283
17	6	0	1.722414	-1.703223	-0.477105
18	6	0	3.846850	-0.324991	-0.209217
19	6	0	2.447957	-0.392196	-0.235209
20	6	0	1.699082	0.763887	-0.008013
21	6	0	2.311964	1.994555	0.235445

22	6	0	3.712628	2.057135	0.254879
23	6	0	4.467531	0.906742	0.035382
24	1	0	-2.240861	0.332757	-1.814397
25	1	0	-1.373765	2.122489	-0.657586
26	1	0	-1.222379	1.324884	1.966523
27	1	0	-2.488632	2.533221	1.721531
28	1	0	0.632953	2.916550	0.404248
29	1	0	5.499346	-1.282491	-0.441669
30	1	0	-4.548841	-0.589777	-1.318496
31	1	0	-5.546887	0.450266	-0.280962
32	1	0	-5.205837	0.921754	-1.957622
33	1	0	-3.816914	2.271068	-0.495800
34	1	0	-3.680103	-1.155107	0.545102
35	1	0	-1.397278	-0.658977	2.566728
36	1	0	-2.824999	-1.644711	2.863747
37	1	0	-1.060406	-3.150811	2.268763
38	1	0	-2.211588	-3.133082	0.933858
39	1	0	1.493149	-2.444724	1.656894
40	1	0	2.414944	-2.452007	-0.871698
41	1	0	0.617202	0.717892	-0.034557
42	1	0	4.201547	3.007564	0.443205
43	1	0	5.552911	0.963564	0.053606

**5c4**					
Center Number	Atomic Number	Atomic Type	X	Y	Z
1	8	0	1.813466	2.489076	0.096612
2	8	0	3.331271	1.196439	1.875156
3	8	0	-1.418958	-1.923839	1.129132

4	8	0	0.509513	-2.914867	1.747792
5	6	0	2.769094	0.015587	1.272890
6	6	0	-0.209122	-2.493648	0.861931
7	8	0	-0.779053	3.261197	-0.055529
8	8	0	-4.729868	-0.632373	-0.068460
9	6	0	4.003588	2.252498	-0.960790
10	6	0	2.611359	1.642926	-0.757422
11	6	0	2.650346	0.210246	-0.230680
12	6	0	2.544604	-0.816134	-1.087021
13	6	0	2.560255	-2.287268	-0.772252
14	6	0	1.270507	-3.015487	-1.235097
15	6	0	0.018695	-2.481846	-0.606118
16	6	0	-1.061734	-1.926544	-1.169169
17	6	0	-2.061303	-1.527773	-0.121344
18	6	0	-3.762297	0.342277	-0.062824
19	6	0	-2.417860	-0.056217	-0.096775
20	6	0	-1.409954	0.917378	-0.093757
21	6	0	-1.719786	2.280644	-0.054134
22	6	0	-3.069108	2.665144	-0.023552
23	6	0	-4.076028	1.706202	-0.030466
24	1	0	2.211407	2.404666	0.985062
25	1	0	3.251745	1.104710	2.834167
26	1	0	3.396754	-0.851705	1.505066
27	1	0	1.776954	-0.178421	1.702237
28	1	0	0.132037	2.883295	-0.024687
29	1	0	-5.601753	-0.216983	-0.020866
30	1	0	4.560382	2.272648	-0.020989
31	1	0	4.571133	1.662789	-1.687050
32	1	0	3.907932	3.274254	-1.340991

33	1	0	2.089831	1.639864	-1.719339
34	1	0	2.411164	-0.575413	-2.143110
35	1	0	3.402418	-2.761756	-1.293438
36	1	0	2.699804	-2.469877	0.295226
37	1	0	1.176123	-2.947975	-2.323747
38	1	0	1.374251	-4.079040	-0.987354
39	1	0	-1.237658	-1.758733	-2.225234
40	1	0	-2.977835	-2.118957	-0.203489
41	1	0	-0.370151	0.606038	-0.126664
42	1	0	-3.315203	3.721845	0.003415
43	1	0	-5.117847	2.015784	-0.006371

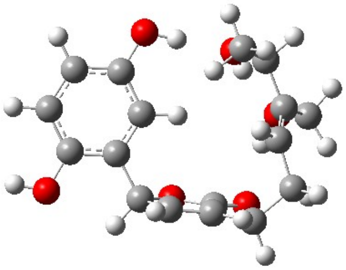

6c1(89.7%)

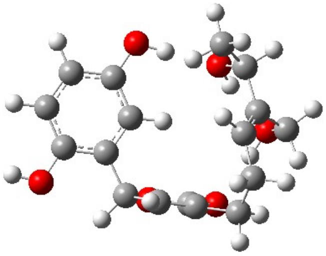

6c2(0.14%)

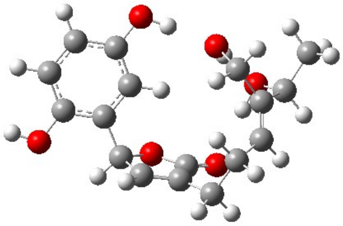

6c3(8.91%)

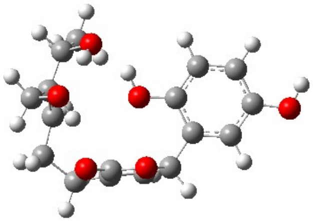

6c4(1.24%)

Table S4. Standard orientation of **6c** in MeOH

**6c1**					
Center Number	Atomic Number	Atomic Type	X	Y	Z
1	8	0	1.772856	2.609826	-0.301924

2	8	0	2.716381	0.843595	-2.067351
3	8	0	-0.990032	-1.917865	-1.367405
4	8	0	1.134669	-2.476564	-1.787236
5	6	0	3.573065	0.387420	-0.991600
6	6	0	0.256663	-2.270801	-0.961568
7	8	0	-1.203020	3.130931	-0.728761
8	8	0	-4.458436	-1.168296	0.623528
9	6	0	2.127371	2.386598	2.058493
10	6	0	2.686061	2.056042	0.684476
11	6	0	2.941213	0.586289	0.375990
12	6	0	2.600971	-0.403758	1.213115
13	6	0	2.785822	-1.890476	1.033982
14	6	0	1.508011	-2.733412	1.318478
15	6	0	0.283557	-2.371196	0.521061
16	6	0	-0.957899	-2.085969	0.951858
17	6	0	-1.853677	-1.741842	-0.211372
18	6	0	-3.688694	-0.076838	0.285772
19	6	0	-2.380240	-0.320321	-0.151611
20	6	0	-1.547148	0.745780	-0.487751
21	6	0	-1.992742	2.068450	-0.399013
22	6	0	-3.308372	2.309525	0.030714
23	6	0	-4.143048	1.245366	0.366511
24	1	0	1.973580	2.169545	-1.150454
25	1	0	2.075353	0.129717	-2.225970
26	1	0	3.871469	-0.650735	-1.147758
27	1	0	4.475546	1.005401	-1.074032
28	1	0	-0.299145	2.804387	-0.933648
29	1	0	-5.352513	-0.879140	0.844345
30	1	0	2.799311	2.038023	2.845658

31	1	0	2.020061	3.470590	2.151947
32	1	0	1.144514	1.929354	2.205203
33	1	0	3.651502	2.576399	0.563955
34	1	0	2.166364	-0.124383	2.172635
35	1	0	3.177412	-2.134652	0.047187
36	1	0	3.531015	-2.233707	1.764602
37	1	0	1.758021	-3.785767	1.131418
38	1	0	1.246376	-2.665164	2.378645
39	1	0	-1.308917	-2.054671	1.977414
40	1	0	-2.692809	-2.437205	-0.312583
41	1	0	-0.536329	0.554644	-0.825291
42	1	0	-3.665485	3.331701	0.095756
43	1	0	-5.160158	1.437499	0.698958

**6c2**					
Center Number	Atomic Number	Atomic Type	X	Y	Z
1	8	0	1.397622	2.313716	-0.929583
2	8	0	2.949487	0.207657	-1.830803
3	8	0	-0.862138	-2.021131	-1.322374
4	8	0	1.299325	-2.163809	-1.932937
5	6	0	3.576958	0.246131	-0.530232
6	6	0	0.456337	-2.150884	-1.046943
7	8	0	-1.265195	3.009838	-0.637712
8	8	0	-4.331677	-1.458478	0.559584
9	6	0	1.506548	2.986340	1.368169
10	6	0	2.238449	2.254029	0.253644
11	6	0	2.648107	0.809528	0.525426
12	6	0	2.198682	0.117160	1.582664

13	6	0	2.495824	-1.318209	1.979267
14	6	0	2.030083	-2.423187	0.993972
15	6	0	0.654694	-2.238462	0.418697
16	6	0	-0.553639	-2.093243	0.981869
17	6	0	-1.601919	-1.878469	-0.075641
18	6	0	-3.596668	-0.336881	0.264826
19	6	0	-2.238707	-0.500167	-0.037096
20	6	0	-1.456483	0.621612	-0.326469
21	6	0	-2.004868	1.905593	-0.341179
22	6	0	-3.366480	2.061684	-0.043527
23	6	0	-4.149470	0.950389	0.258738
24	1	0	1.764974	1.660191	-1.559594
25	1	0	2.412917	-0.606680	-1.881781
26	1	0	3.957408	-0.741946	-0.257828
27	1	0	4.442714	0.908702	-0.645150
28	1	0	-0.327658	2.747685	-0.791586
29	1	0	-5.259030	-1.212373	0.680173
30	1	0	2.131218	3.051240	2.262685
31	1	0	1.272198	4.002207	1.038606
32	1	0	0.567288	2.491557	1.629259
33	1	0	3.160238	2.814367	0.028532
34	1	0	1.536436	0.637278	2.270018
35	1	0	3.571191	-1.457653	2.143954
36	1	0	2.020956	-1.497850	2.948236
37	1	0	2.721973	-2.496412	0.150578
38	1	0	2.084123	-3.386750	1.513539
39	1	0	-0.797465	-2.081448	2.037526
40	1	0	-2.374377	-2.651601	-0.057379
41	1	0	-0.403393	0.502960	-0.554113

42	1	0	-3.801881	3.055713	-0.050948
43	1	0	-5.203915	1.080686	0.488769

**6c3**					
Center Number	Atomic Number	Atomic Type	X	Y	Z
1	8	0	2.711414	0.876710	-1.768912
2	8	0	1.357717	2.181727	0.221610
3	8	0	-0.849214	-1.484968	-1.523068
4	8	0	1.366323	-1.856722	-1.649402
5	6	0	2.084638	1.396267	1.192723
6	6	0	0.336854	-1.897219	-0.996849
7	8	0	-1.213522	3.173592	0.526009
8	8	0	-4.680977	-1.071632	-0.348522
9	6	0	4.505275	1.949703	-0.599501
10	6	0	3.676022	0.674841	-0.695516
11	6	0	2.895113	0.292760	0.552447
12	6	0	2.890784	-0.993739	0.931678
13	6	0	2.048996	-1.699858	1.954573
14	6	0	1.210020	-2.839586	1.306719
15	6	0	0.116499	-2.351597	0.400663
16	6	0	-1.191030	-2.200772	0.656693
17	6	0	-1.896052	-1.589941	-0.520988
18	6	0	-3.847783	-0.000349	-0.138970
19	6	0	-2.466108	-0.215845	-0.219671
20	6	0	-1.589446	0.846565	0.006927
21	6	0	-2.057337	2.128231	0.303442
22	6	0	-3.441441	2.339979	0.375932
23	6	0	-4.323412	1.283521	0.157267

24	1	0	2.253068	0.024039	-1.892983
25	1	0	1.617015	1.865316	-0.669877
26	1	0	2.753993	2.078514	1.735088
27	1	0	1.369958	0.998361	1.918426
28	1	0	-0.283943	2.858384	0.440674
29	1	0	-5.600110	-0.771737	-0.330578
30	1	0	5.262502	1.849873	0.183487
31	1	0	5.012146	2.134856	-1.550494
32	1	0	3.878625	2.816110	-0.371291
33	1	0	4.334846	-0.158859	-0.968287
34	1	0	3.548805	-1.663916	0.377096
35	1	0	2.700277	-2.163950	2.706758
36	1	0	1.382968	-1.017409	2.490068
37	1	0	1.881378	-3.497561	0.742652
38	1	0	0.762754	-3.440645	2.103848
39	1	0	-1.708563	-2.428596	1.581159
40	1	0	-2.675579	-2.243143	-0.923197
41	1	0	-0.520617	0.685513	-0.062484
42	1	0	-3.817826	3.331756	0.605108
43	1	0	-5.395193	1.455670	0.217702

**6c4**					
Center Number	Atomic Number	Atomic Type	X	Y	Z
1	8	0	-1.383954	2.616106	0.609685
2	8	0	-2.575385	0.722434	2.210809
3	8	0	0.629572	-1.876410	1.196151
4	8	0	-1.577249	-1.921498	1.606658

5	6	0	-3.561413	0.659542	1.156543
6	6	0	-0.661027	-1.988308	0.798733
7	8	0	5.886918	0.267734	-0.046601
8	8	0	0.369645	0.695375	0.001973
9	6	0	-1.915940	2.770303	-1.720212
10	6	0	-2.449373	2.364929	-0.355197
11	6	0	-2.949358	0.932319	-0.203121
12	6	0	-2.803447	0.000228	-1.156930
13	6	0	-3.163171	-1.460244	-1.083719
14	6	0	-1.979102	-2.390316	-1.455245
15	6	0	-0.714038	-2.172365	-0.671694
16	6	0	0.547290	-2.082874	-1.113964
17	6	0	1.500785	-1.873749	0.030463
18	6	0	1.723872	0.662311	0.008861
19	6	0	2.336095	-0.609465	-0.000728
20	6	0	3.728373	-0.703160	-0.016389
21	6	0	4.526229	0.444950	-0.026592
22	6	0	3.918405	1.702503	-0.005611
23	6	0	2.526103	1.807656	0.011404
24	1	0	-1.639733	2.126543	1.421420
25	1	0	-2.052609	-0.098569	2.136009
26	1	0	-4.083912	-0.299219	1.189370
27	1	0	-4.292434	1.440183	1.396138
28	1	0	6.320410	1.132091	-0.037052
29	1	0	-0.034599	1.576157	0.191092
30	1	0	-1.066582	2.147459	-2.014285
31	1	0	-2.694492	2.690830	-2.483419
32	1	0	-1.578979	3.809921	-1.680626
33	1	0	-3.282138	3.037620	-0.095622

34	1	0	-2.351454	0.299576	-2.101673
35	1	0	-3.528889	-1.732838	-0.093582
36	1	0	-3.970778	-1.675980	-1.796112
37	1	0	-2.313951	-3.426531	-1.316521
38	1	0	-1.741521	-2.282114	-2.518937
39	1	0	0.887068	-2.140181	-2.141491
40	1	0	2.171661	-2.735073	0.132531
41	1	0	4.210947	-1.676037	-0.014906
42	1	0	4.526888	2.603525	-0.004065
43	1	0	2.057019	2.787146	0.022713

References

(1) Discovery Studio v4.0 client Copyright @2005-13 Accelrys software Inc.

(2) Bruhn T.; Schaumlöffel A.; Hemberger Y.; Bringmann G.; *SpecDis*, version 1.61, University of Wuerzburg, Germany, **2013**.

(3) Frisch, M. J.; Trucks, G. W.; Schlegel, H. B.; Scuseria, G. E.; Robb, M. A.; Cheeseman, J. R.; Scalmani, G.; Barone, V.; Mennucci, B.; Petersson, G. A.; Nakatsuji, H.; Caricato, M.; Li, X.; Hratchian, H. P.; Izmaylov, A. F.; Bloino, J.; Zheng, G.; Sonnenberg, J. L.; Hada, M.; Ehara, M.; Toyota, K.; Fukuda, R.; Hasegawa, J.; Ishida, M.; Nakajima, T.; Honda, Y.; Kitao, O.; Nakai, H.; Vreven, T.; Montgomery, J. A.; Peralta, J. E.; Ogliaro, F.; Bearpark, M.; Heyd, J. J.; Brothers, E.; Kudin, K. N.; Staroverov, V. N.; Keith, T.; Kobayashi, R.; Normand, J.; Raghavachari, K.; Rendell, A.; Burant, J. C.; Iyengar, S. S.; Tomasi, J.; Cossi, M.; Rega, N.; Millam, J. M.; Klene, M.; Knox, J. E.; Cross, J. B.; Bakken, V.; Adamo, C.; Jaramillo, J.; Gomperts, R.; Stratmann, R. E.; Yazyev, O.; Austin, A. J.; Cammi, R.; Pomelli, C.; Ochterski, J. W.; Martin, R. L.; Morokuma, K.; Zakrzewski, V. G.; Voth, G. A.; Salvador, P.; Dannenberg, J. J.; Dapprich, S.; Daniels, A. D.; Farkas, O.; Foresman, J. B.; Ortiz, J. V.; Cioslowski, J.; Fox, D. J. Gaussian 09, revision C.01. Gaussian, Inc.: Wallingford CT, **2010**.

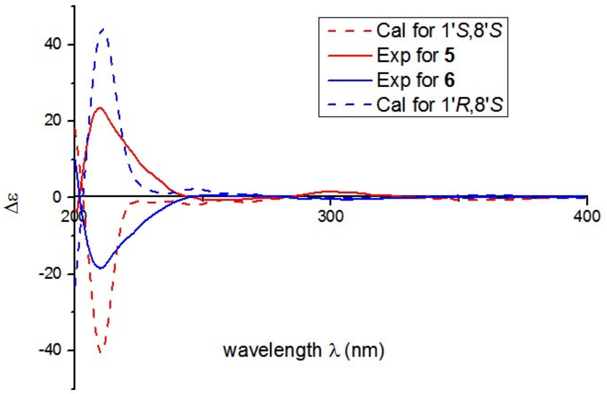

Figure S63. The calculated and the experimental ECD spectra of **5** and **6**.

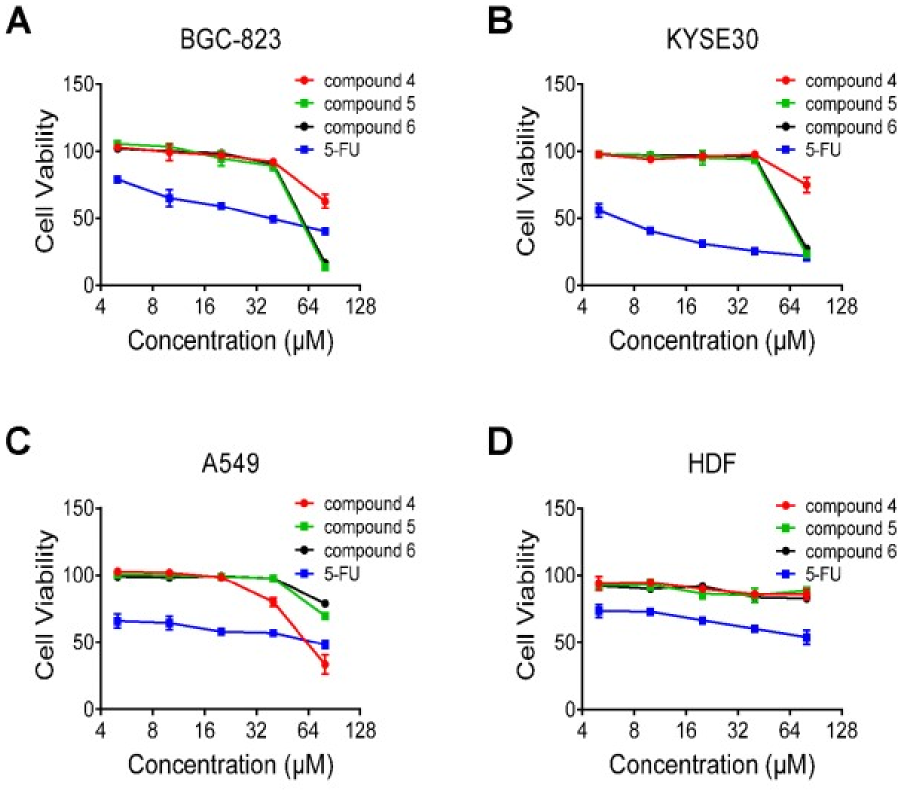

Figure S64. Cytotoxic effects of compound-4,compound-5, compound-6 and 5-FU on human cancer cells (A, B, C) and normal cells (D) Cells were treated with various concentrations of compound-4,compound-5, compound-6 and 5-FU for 48 h. Cytotoxic effect of the compounds was determined by CCK8 assay. Percentages of viable cells were calculated by comparing treated and solvent control cells. Data are the mean ± S.D. of three replicates.
